# Modularly prepared segmented fluorinated silicones and their anomalous microstructure dependent flow behavior†

**DOI:** 10.1039/d5ra03087k

**Published:** 2025-06-24

**Authors:** Analise C. Migliaccio, Nathan J. Weeks, Scott T. Iacono

**Affiliations:** a Department of Chemistry, Chemistry Research Center, Laboratories for Advanced Materials, United States Air Force Academy Colorado Springs Colorado 80840 USA scott.iacono@afacademy.af.edu

## Abstract

Octafluorocyclopentene was used as a coupling reagent by undergoing a series of efficient and regio-controlled addition–elimination reactions with eugenol and alkyldiols/bisphenols to prepare terminally unsaturated monomers. Structural characterization revealed these high molecular weight monomers can be obtained with high purity and in multi-gram quantities with minimal purification. The eugenolic-based monomers underwent Pt-catalyzed hydrosilylations with hydride-terminated polydimethylsilane oligomers to prepare a versatile pool of segmented fluorosilicones. Polymer characterization included molecular weight determination, calorimetric studies, thermo-gravimetric analysis, and rheology. Of particular interest as next-generation lubricants for in-space applications, these fluorosilicones possess adaptable structure–property relationships by nature of the segment substitution.

## Abstract 2

Octafluorocyclopentene was used as a coupling reagent by undergoing a series of efficient and regio-controlled addition–elimination reactions with eugenol and alkyldiols/bisphenols to prepare terminally unsaturated monomers.

## Graphical abstract

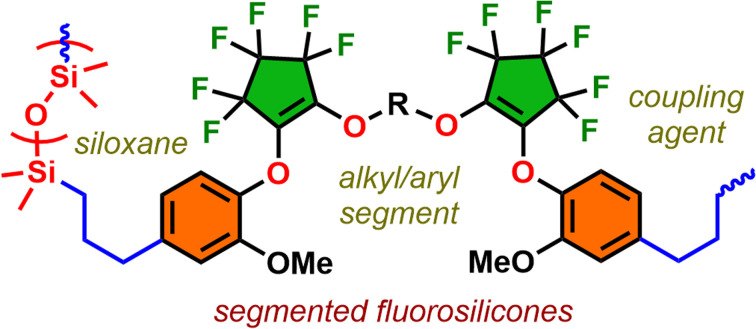

## Introduction

**Scheme 1:**
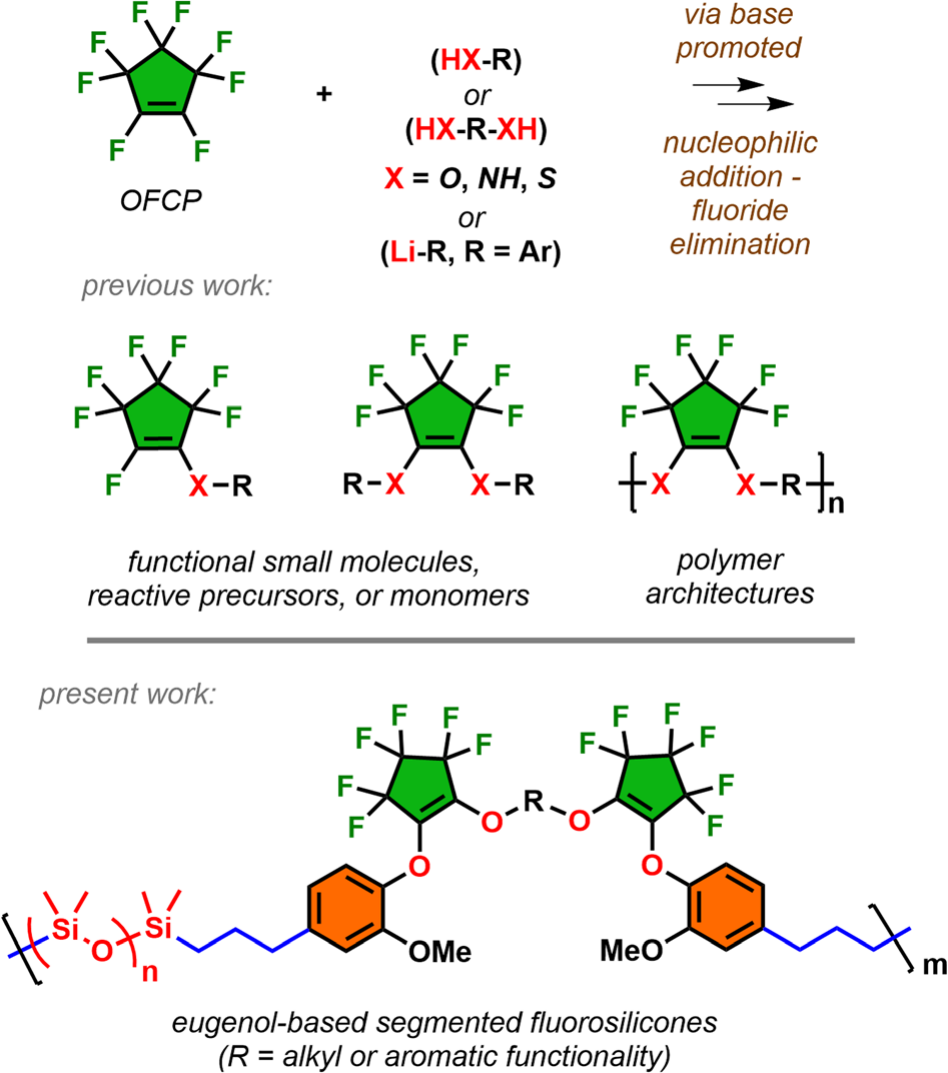
Nucleophilic addition–elimination of OFCP affords both functional molecules and polymers. The use of OFCP as a key intermediate expands into diverse fluorosilicone architectures.

Octafluorocyclopentene (OFCP), synonymously named perfluorocyclopentene, is a commercial chemical commonly used for plasma etching in the global electronics industry. As detailed in a recent mechanistic study,^1^ OFCP possesses a polyelectrophilic fluoroalkene and undergoes facile addition followed by fluoride elimination with several O–, N–, S–, C– nucleophiles under basic conditions (Scheme 1).^2–7^ In addition, the regio-selectivity of addition–elimination can occur by single or double substitution at the fluoroalkene depending on the nature of the base and stoichiometry of the impending nucleophile. This has led to numerous pursuits utilizing OFCP as a versatile intermediate for the preparation of fluorine-containing materials including macrocyclic peptides,^8^ photochoromic dyes,^9,10^ flexible hole-transporting coatings,^11^ thermosetting resins,^12^ architecturally controlled polymers,^13,14^ and functionalized silicas and aerogels.^15,16^ Although the utility of OFCP has led to diverse applications, it remains undervalued as a modular building block for emerging technological needs.

The work expands the scope of OFCP as a key intermediate for the enchainment of long chain alkyldiols and bisphenolic segments followed by addition of eugenol to serve as monomers undergoing hydrosilylations for the preparation of modified fluorosilicones (Scheme 1). Industrial use of fluorosilicones serve as higher performance materials over their hydrocarbon-based silicone analogues due to the inherent C–F bond strength in the polymer microstructure.^17,18^ This leads to improvements in resistance to contact fluids (oil, fuel, solvents) in addition to expanding high and low operating temperatures. Applications include lubricants in aerospace applications and protective anti-fouling coatings, and release agents.^19–21^ Due to installment of alkyl/phenolic segments, these eugenol-based segmented fluorosilicones demonstrated unique rheological properties attractive for in-space high performance fluids. The work also details operationally simple synthesis of monomers in scalable quantities and encompasses comprehensive polymer characterization in addition to structure-thermal/rheological property relationships.

## Results & discussion

### Monomer synthesis and characterization of model system

**Scheme 2:**
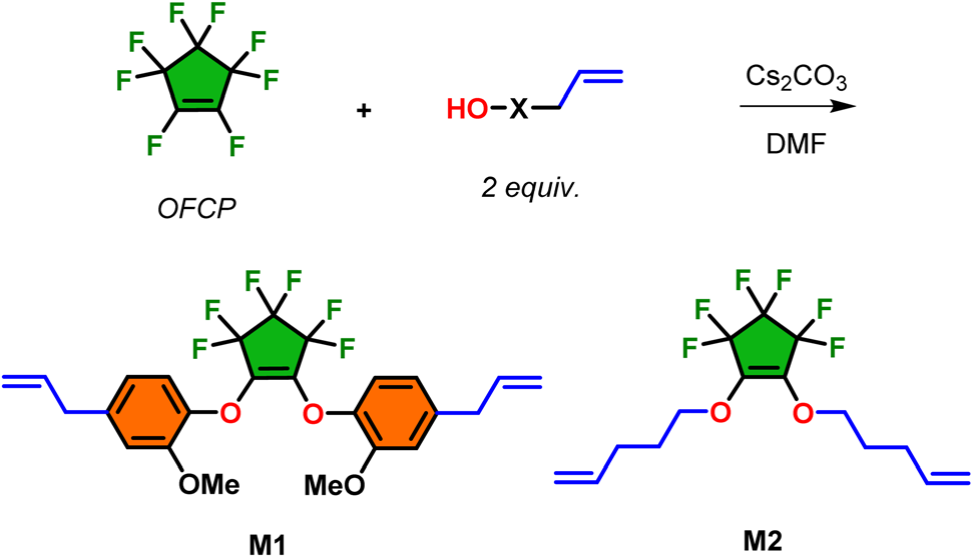
Base-promoted synthesis of monomers M1 and M2 by nucleophilic addition–elimination of either eugenol or 4-penten-1-ol with OFCP.

Monomers M1 and M2 were prepared in nearly quantitative isolated yield (>95%) adapting a previously published procedure by the addition of two equivalents of either eugenol or 4-penten-1-ol, respectively, to octafluorocyclopentene (OFCP) in presence of cesium carbonate in DMF followed by cesium fluoride elimination (Scheme 2). This facile, base-promoted nucleophilic addition–elimination occurs in two discrete steps affording regio-specific formation of the diaryl/alkyl ether functionalities through the electrophilic 1,6-fluoroalkene of OFCP. These reactions were monitored by ^19^F NMR where the consumption after 2–4 h at room temperature of the sp^2^-hybridzed fluorine signal of the OFCP alkene (C=C*F*) at −156 ppm in CDCl_3_ is observed. The 2,2,3,3,4,4-substituted geminal fluorines are retained on the cyclopentene as a 2 : 1 integration ratio due to ring symmetry further elucidates the compound structure. Work-up and purification simply involved removal of carbonate salts under vacuum, followed by purification using ethyl acetate/hexanes mixture over silica. While previous work reports M1 as a yellow oil, the adapted procedure afforded X-ray diffractable crystalline solids upon cooling the colorless oil to −78 °C followed warming to room temperature. Monomers M1 and M2 are introduced to this study to serve as model system for preparing unsegmented polymer formulations demonstrating the influence of the eugenol moiety in rheology studies detailed later in this work.

### Monomer synthesis and characterization of segmented system

**Scheme 3:**
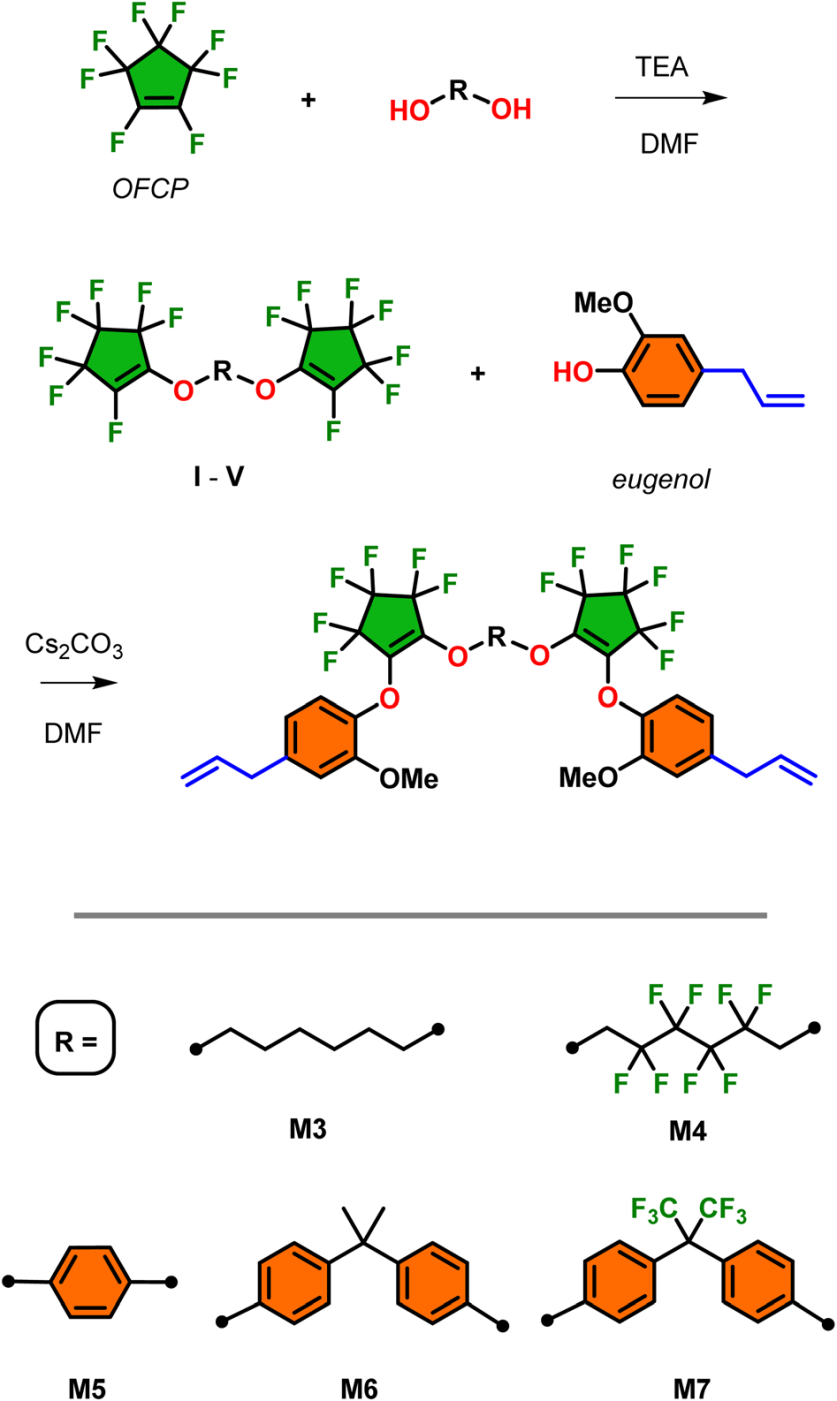
Synthesis of intermediates I–V and their respective monomers M3–M7 from the base promoted, stepwise addition–elimination of OFCP.

**Fig. 1:**
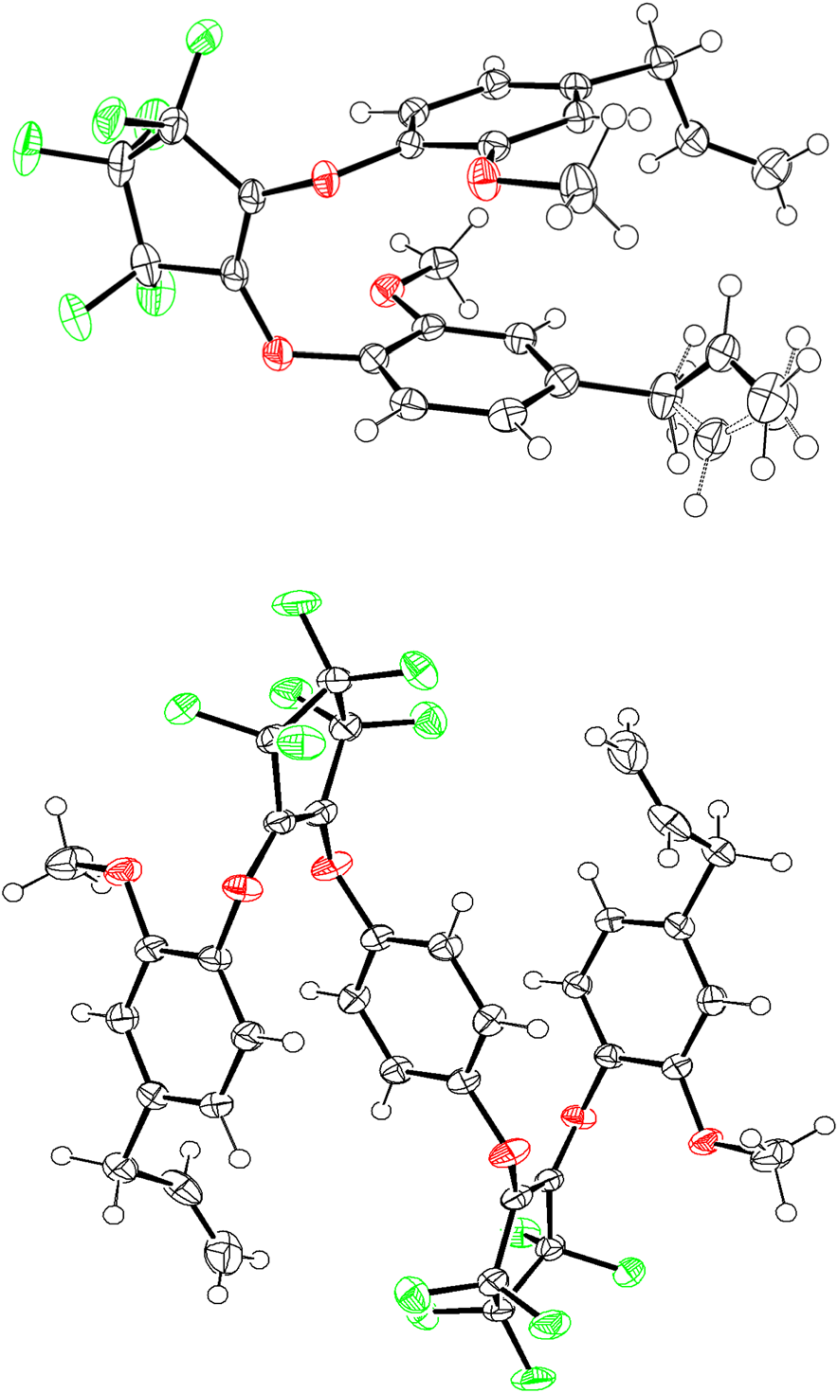
Single crystal XRD structures of M1 (top) and M5 (bottom). ORTEP representations of thermal ellipsoids are shown at 50% probability.

Segmented monomers M3–M7 were prepared using the similar methodology for the preparation of M1 and M2 which is outlined in Scheme 3. The first step is the preparation of intermediates I–V which involved the base-promoted addition of a range of diols/bisphenols with 1 : 1 equivalency with OFCP. After work-up and purification, this afforded the preparation of segmented bis-2,2,3,3,4,4-heptafluorocyclopentene alkyl/aryl ethers I, II, and V in excellent isolated yields (>92%) and modest yields of III and IV (42% and 65%, respectively). The choice of diols/bisphenols included a range of 6-carbon chain aliphatic species and bisphenols and their fluorinated surrogates. The final step involves the installation of two additional equivalents eugenol to the terminal fluoroalkene of the intermediates I–V in overall good to excellent isolated yields of monomers M3–M7 (80–96%) and retention of the desired vinyl substitution on the cyclopentene ring. However, analysis of the ^19^F NMR of monomer M3 revealed evidence of the minor allyl substituted isomer in less than 5% which is carried over to the polymer synthesis and as demonstrate later in this work contributes no deviation to molecular weight or thermal properties (see ESI, Fig. S18† for structure elucidation). This is a result of the fluoride-catalyzed rearrangement of the desired vinyl substituted fluorocyclopentene and its mechanism has been thoroughly investigated.^22^ All other monomers showed no presence of the allyl isomer and may be a result of differences in the nucleophile electronic effects that were not explored further in this study. Except for monomers M1 and M5 which were isolated as solids, all others afforded oils with increasing viscosity upon installation of aromatic moieties. As such, monomers M1 and M5 afforded single crystal X-ray quality solids and their ORTEP structures are illustrated in Fig. 1. Of potential interest is the alignment of the aryl rings in both the X-ray structures. In the absence of any significant hydrogen or halogen bonding this ring-stacking effect likely drives the formation of crystals in these two species. Detailed ^1^H, ^13^C, and ^19^F NMR spectroscopy data are provided in the ESI section.†

### Thermal analysis of monomers

Given the high molecular weight range of monomers M3–M7, DSC analysis provided insight to their glass transition temperature (*T*_g_) behavior correlating their segment substitution (R) as detailed in Table 1. Comparing alkyl segmented monomers M3 and M4, the octafluorohexyl segmented species has a significantly higher molecular weight yet afforded a slightly lower *T*_g_ of −43 °C to the non-fluorinated hexyl analog (*T*_g_ of −48 °C) due to the lowering of intermolecular forces inducing favorable chain mobility. However, both monomers M3 and M4 showed a higher *T*_g_ due to the installation of segments compared with M1 (*T*_g_ of −54 °C) with overall lower molecular weight. While monomer M5 (R = Ph) initially melted at 99 °C, upon cooling and reheating afforded a *T*_g_ of −29 °C indicating a degree of vitrification. Comparing M5 to the bisphenolic segmented monomers M6 (R = bisphenol A) and M7 (R = bisphenol AF), both showed increasing *T*_g_ of −11 °C and 3 °C, respectively, with increasing molecular weight and rigidity of their bisphenolic units. Interestingly, the bisphenol AF monomer M7 also produced a minor *T*_g_ of −43 °C which indicates a dual *T*_g_ structure, suggesting this inflection is due the eugenol end-unit contribution. As detailed later in this work, these effects based on segment substitution demonstrate pronounced effects upon their incorporation as silicone copolymers in relation to their rheological behavior.

**Table 1:** DSC analysis of monomers M1–M7

Entry	Mol wt	*T*_g_ a (°C)
M1	500.44	−54
M2	344.30	—
M3	790.64	−48
M4	934.57	−43
M5	782.58	−29^b^
M6	900.76	−11
M7	1008.7	3^c^

a DSC (5 °C min^−1^) in nitrogen determined by third heating cycle.

b *T*_m_ 99 °C; *T*_c_ 18 °C.

c Minor −43 °C (eugenol contribution).

### Polymer synthesis and structural characterization

**Scheme 4:**
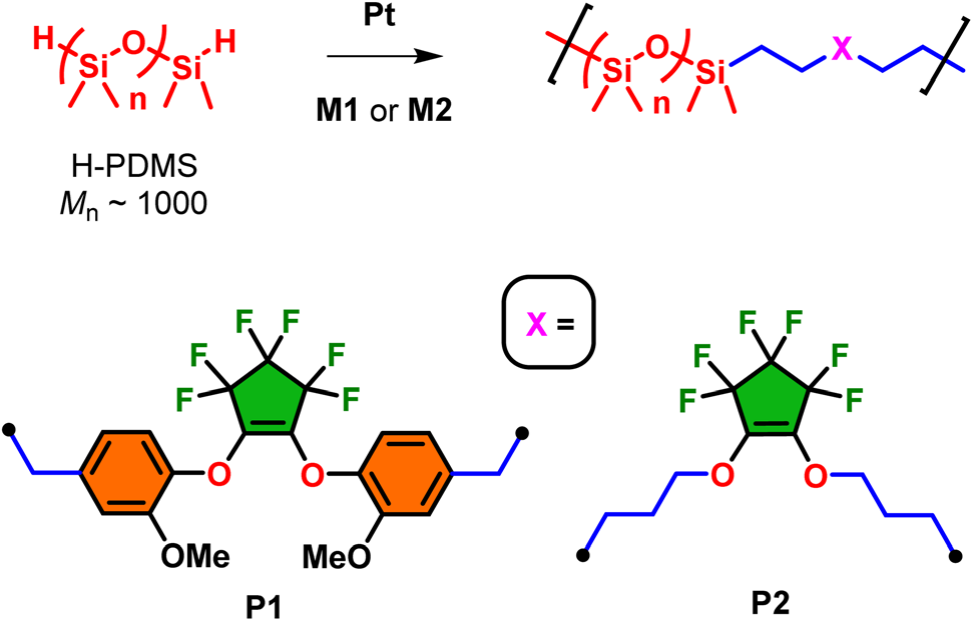
Pt-catalyzed hydrosilylations using H-PDMS oligomer with M1 or M2 affording copolymers P1 and P1.

**Scheme 5:**
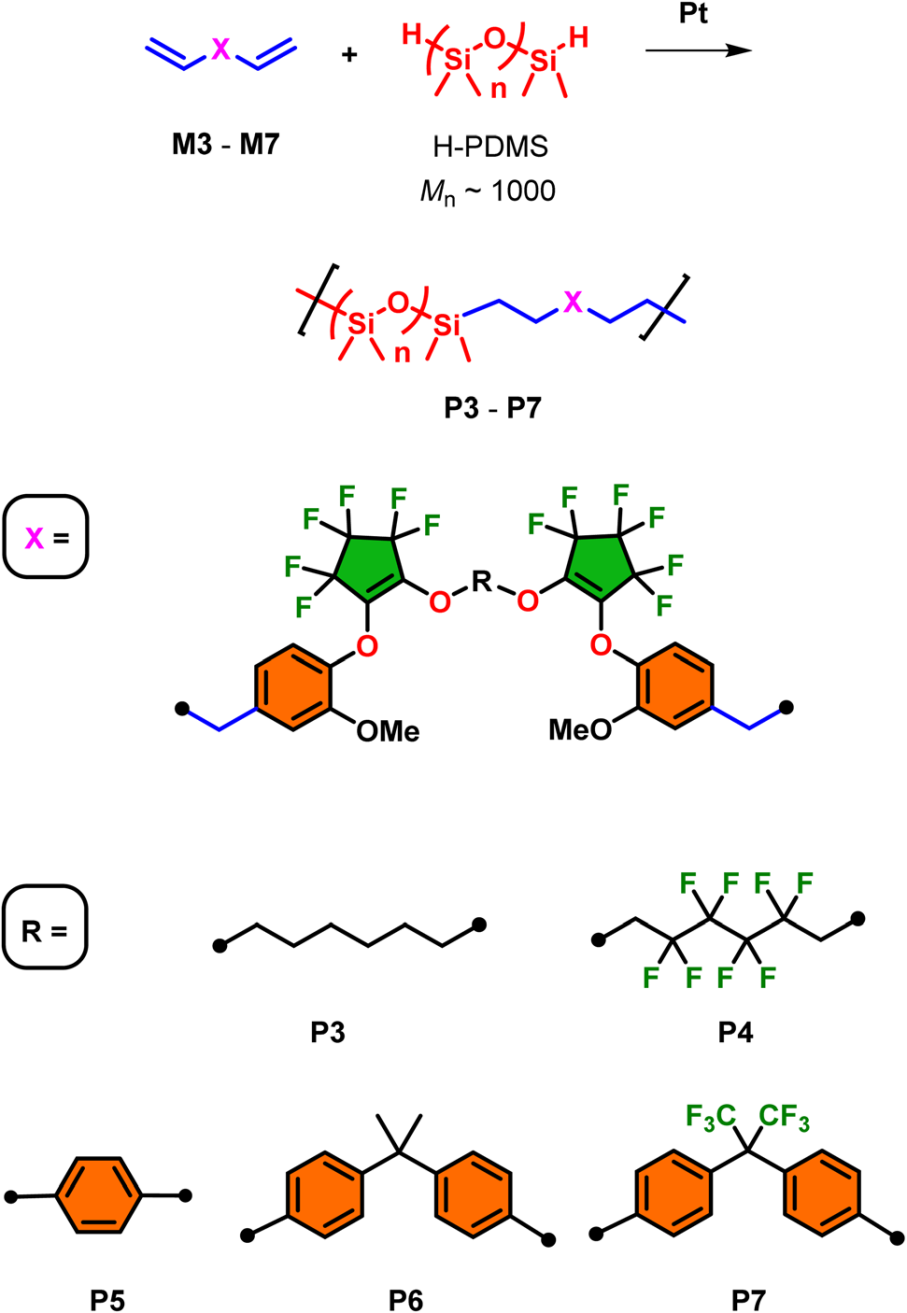
Preparation of segmented copolymers P3–P7 by Pt-catalyzed hydrosilylations using H-PDMS oligomer and the respective monomers M3–M7.

Utilizing monomers M1–M7, fluorosilicone copolymers P1–P7 were prepared by Pt-catalyzed (using Karstedt's catalysis at 1 mol%) hydrosilylation using hydride-terminated polydimethylsiloxane (H-PDMS) at room temperature for 24 h (Schemes 4 and 5). For this study, H-PDMS with a 1000 g mol^−1^ number-average molecular weight (determined by ^1^H NMR) was selected to match the molecular weight of the monomer units to influence the bulk physical properties. The reactions were performed neat using both M1 and M2 since they possess good miscible when mixed with H-PDMS. On the other hand, for the preparation of copolymers P3–P7 using monomers M3–M7, respectively, a minimal amount of toluene was required to induce stirring and miscibility for adequate mixing with H-PDMS. In all cases, near quantitative conversion of the hydrosilylation polymerization was observed using ^1^H NMR (in CDCl_3_) whereby the diagnostic silyl hydride (Si–*H*) was consumed at 4.2 ppm in addition to the conversion of the terminal alkenes AMX-pattern (−C*H*=C*H*_2_). All formulations produced transparent oils with varying degrees of viscosity which will be analyzed using rheology later in this report.

A summary of molecular weight analysis and thermal properties of fluorosilicone copolymers P1–P7 is summarized in Table 2. Polymer molecular weight analysis was carried out using ^1^H NMR end-group analysis in addition to GPC separation in THF using polystyrene as standards. Specifically, number-average molecular weight (*M*_n_) from ^1^H NMR analysis was determined by relative integration of the dimethylsiloxane repeat unit (–Si(CH_3_)_2_*CH̲*_2_^−^) relative to residual total alkene end-groups (–C*H̲*=C*H̲*_2_). Table S1 in the ESI details† the end-group analysis calculations in addition to integrated ^1^H NMR spectra for copolymers. Except for P2, P1 and the segmented copolymers P3–P7, demonstrated consistent *M*_n_ values among the series. Copolymer P2 showed poor hydrosilylation reactivity and this may be rationalized such that 2-propenylbenzenes undergo metal-catalyzed isomerization to 1-propenylbenzene that is accomplished by an η^3^-allyl metal–hydride complex suggesting the M1 alkenes might be more reactive towards hydrosilylation than the aliphatic alkenes of M2.^23^ For the segmented copolymers P1–P7, GPC analysis revealed consistent *M*_n_ values among the series whereby *M*_w_ values of copolymers P5–P7 are significantly elevated compared indicating the influence of segment molecular weight effects increases void volumes during polymer chain separation in the GPC column. For P2, the observed high dispersity (*Đ*) confirms the poor monomer conversion as previously discussed from ^1^H NMR analysis.

**Table 2:** Selected molecular weights and thermal properties of copolymers P1–P7

Entry	*M*_n_ a × 10^−3^ (NMR)	*M*_n_ b × 10^−3^ (GPC)	*M*_w_ b × 10^−3^ (GPC)	*Đ* b	*T*_m_ c (°C)	*T*_d_ d (°C)	Char^d^ (%)
P1	16.2	13.1	34.6	2.60	−53, −41	367	7.4
P2	9.00	10.7	64.0	6.00	−45	353	3.1
P3	13.0	13.1	43.1	3.30	−46	369	5.1
P4	13.1	7.80	22.3	2.84	−44	384	3.0
P5	17.0	8.10	57.4	7.07	−44	404	3.2
P6	15.7	10.7	76.9	7.17	−45	409	2.8
P7	15.7	7.40	32.6	4.42	−44	401	2.7
H-PDMS	1.00	—	—	—	−54, −41	*205* e	—

a End-group analysis by ^1^H NMR in CDCl_3_.

b GPC in THF using polystyrene as standard.

c DSC (5 °C min^−1^) in nitrogen determined by third heating cycle.

d TGA onset (10 °C min^−1^) to 900 °C in nitrogen.

e b.p. at 205 °C reported by commercial manufacturer.

### Thermal analysis of polymers

Thermal analysis of P1–P7 using DSC after the third heating–cooling cycle over the range of 50 °C to −90 °C did not reveal any glass transition temperature (*T*_g_) event. Compared with monomer *T*_g_ values shown in Table 1, the contribution of M1–M7 appears subdued by the flexible PDMS segments in the reported temperature range. Given the limitations of DSC cooling, the *T*_g_ of PDMS segments are typically −130 °C (ref. 24) and segment contributions could be present below −90 °C. In the segmented copolymers P3–P7, the melting point transition *T*_m_ was reported in a narrow range of −44 °C to −46 °C which is nearly 10 °C higher than H-PDMS *T*_m_ of −54 °C (major) indicating segments suppress the reorganization of PDMS chains. This is further evidenced by similar major and minor melting point values of P1*T*_m_ of (−54, −41 °C) possessing no segments in the polymer microstructure indicating this copolymer retains bulk PDMS crystallization behavior.

Thermogravimetric analysis (TGA) was performed on copolymers P1–P7 in nitrogen to 900 °C. Only the segmented copolymers P5–P7 showed an increase in onset of decomposition (*T*_d_) by 20 °C on average and as high as to 409 °C compared to the rest polymer series due to inherent aromatic stability. Compared with commercial high molecular weight PDMS the *T*_d_ of P1–P7 is > 369 °C and is on the higher end of these materials. Char yields at 900 °C were inconsistent among the copolymer series but overall remained low ranging 2.5–7.4% from carbonaceous char from the aromatic ring fusion contributions of the fluorocyclopentenyl ring, phenolic, and/or eugenol components.

### Polymer rheology analysis

**Fig. 2:**
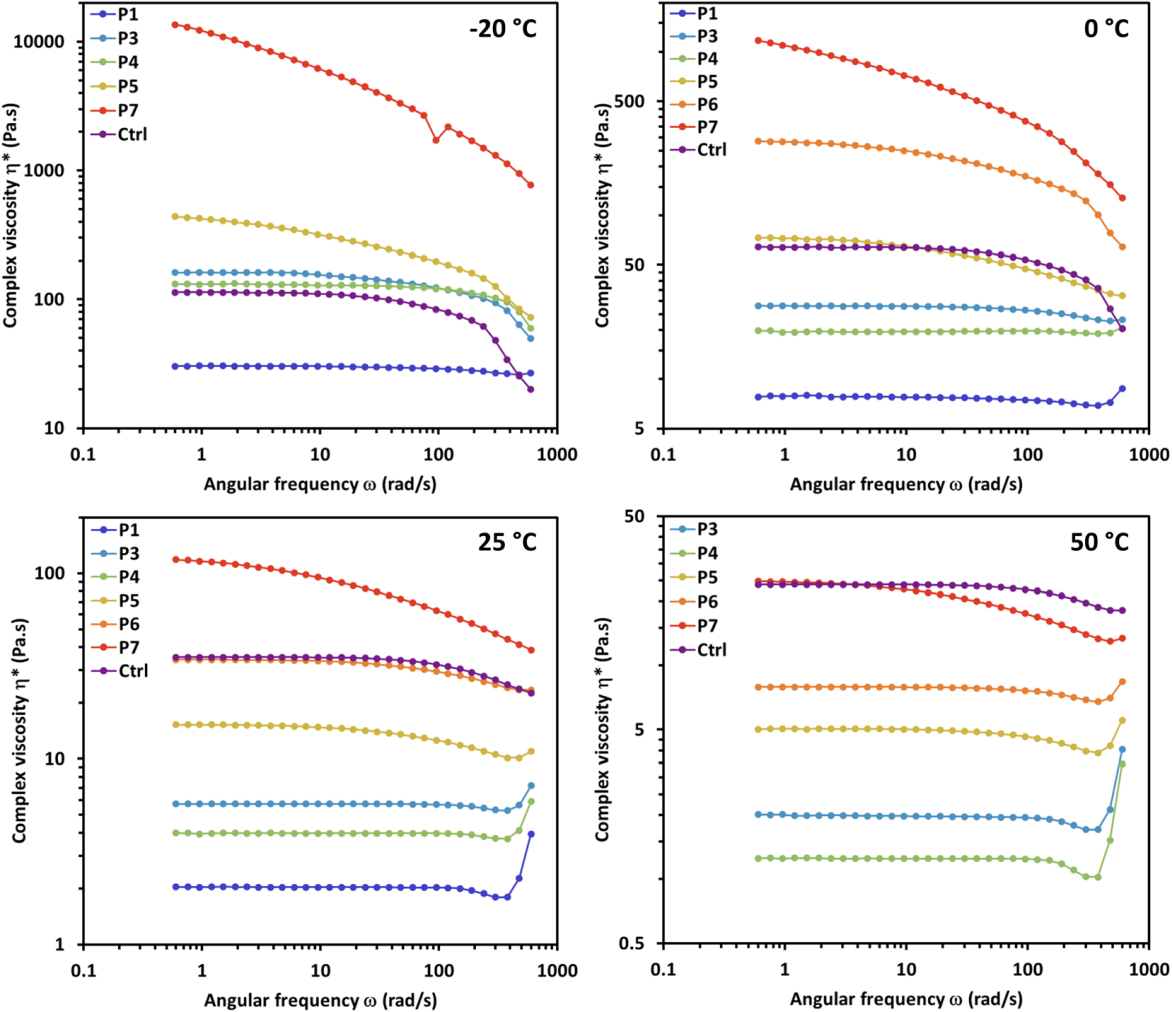
Oscillation frequency sweeps at 30% strain (below critical strain for all samples) of control H-PDMS (Ctrl) copolymers P1 and P3–P7 and in environmental testing chamber (ETC) at temperatures: −20 °C, 0 °C, 25 °C and 50 °C.

**Fig. 3:**
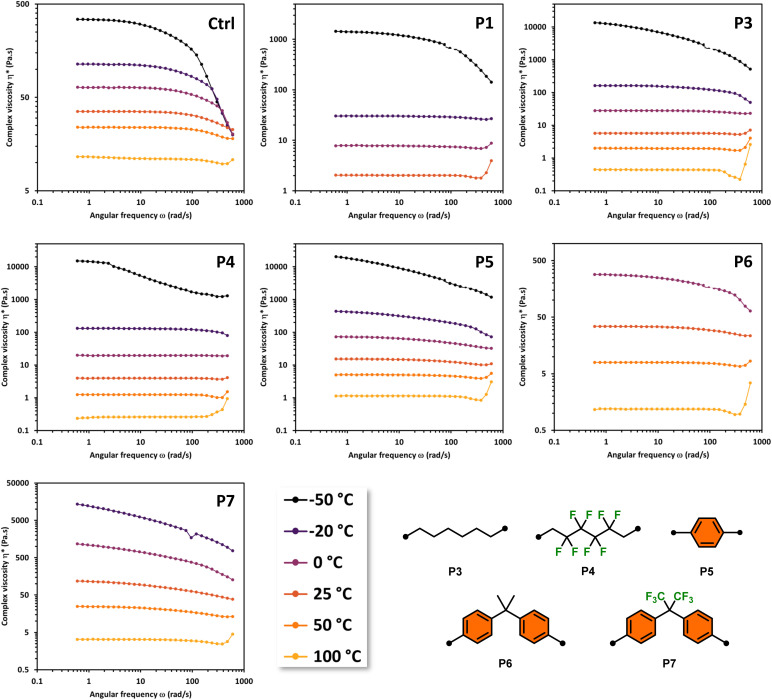
Oscillation frequency sweeps with 30% strain at temperatures: −50, −20, 0, 25, 50, and 100 °C of Ctrl, P1, and P3–P7.

Rheological analysis was performed on copolymers P1 and P3–P7 (P2 was excluded from rheological analysis due to its incomplete reactivity) along with H-terminated PDMS (*M*_n_ of 10 000) as a control (Ctrl). Ctrl was chosen as a standard for comparison due to analogous bulk viscoelastic properties to the copolymers (similar qualitative viscosity and surface tension) as well as a comparable molecular weight. All samples displayed shear-dependent behavior as evidenced by the complex viscosity over a range of temperatures (see Fig. 2 and 3).

The control H-PDMS (Ctrl) displays characteristic shear thinning behavior under increasing shear rate for the full temperature range. A model case for this non-Newtonian shear thinning is the room temperature (25 °C) frequency sweep at 30% strain (Fig. 2), where the viscosity stays constant under increasing shear rate until around 20 rad s^−1^ when the viscosity rapidly starts to decrease in a smooth curve as the shear rate sweeps up to 600 rad s^−1^. This effect is postulated as due to alignment of the individual siloxane polymer chains under sufficient shear rate, eliminating chain entanglement and thus lowering viscosity.

The non-Newtonian shear thinning of PDMS is well known.^25^ During oscillatory frequency sweeps the complex viscosity H-PDMS (Ctrl) begins to decline at elevated shear rates over a range of designated temperatures (Fig. 3). When compared to Ctrl, the copolymers P1 and P3–P7 display varying behavior in the frequency sweeps that are dependent on both temperature and segment composition. None of the copolymers display completely typical shear thinning curves like the H-PDMS. Instead, the segment effects are observable as temperature dependent deviations.

It is important to note that copolymers P1 and P3–P7 have widely varying qualitative pour and quantitative complex viscosities at room temperature. This is despite the fact they all have comparable molecular weights as to presume these viscosity effects are driven largely by the segment effects. P1 and P3–P5 all have lower viscosities than Ctrl at room temperature, while P6 and P7 have higher room temperature viscosities. Practically, the temperature dependence of viscosity in the copolymers meant that P1 and P3–P5 were all amenable to low temperature (<0 °C) testing as comparable to Ctrl. Conversely, P6 and P7 were well behaved during high temperature (>25 °C) testing but neared solidity at low temperatures making the oscillation frequency sweeps at those temperatures impractical.

The alkyl chain-based segments of P3 and P4 led to some particularly interesting viscosity effects under oscillation shear. Both copolymers were more resistant to shear thinning along the frequency range than the control H-PDMS (Ctrl). P4 in fact appears to display non-Newtonian shear thickening effects at highly elevated shear rates, especially at elevated temperatures at and above 25 °C (Fig. 3). We hypothesize that the steady viscosity under increasing shear rate and the high shear rate shear-thickening effects are driven primarily by the segments potential to chain entangle. The rheology properties of P3 and P4 contrast with P7 whose bulky segments prevent ready chain entanglement and thus display pronounced shear thinning even at low frequencies. This contrast is also readily observable from Fig. 2 in the overlay of the frequency sweeps of the copolymers at 50 °C. Strikingly, copolymers P3–P5 display resistance to shear thinning at mid-range shear rates while P7 shear thins more drastically than the control even starting at the mid-range shear rate.

Overall, the rheology study of copolymers P1 and P3–P5 reveal a pronounced shear thickening effect at the highest shear rates (>100 rad s^−1^) at elevated temperatures. The effect takes the form of a slight dip (shear thinning) followed by a spike in increasing viscosity at the highest shear rates. This is a common effect in structured fluids, materials which contain more than one phase dispersed in a carrier liquid (*e.g.* toothpaste, cosmetics, caulk). These materials show a yielding phase at mid-range increasing shear/rate followed by an increase in viscosity due to structure rearrangements as a result of applied shear, or flow induced shear thickening. This effect in some cases shows an erratic increase in complex viscosity which could support the rapid formation and breakdown of stress bearing structures within the material above a critical shear rate.^26^ It also appears that copolymers P6 and P7 behave as pseudoplastic materials at temperatures ≤25 °C, meaning they do not appear to have yield stress required for flow but nonetheless behave nonlinearly and display shear thinning behavior. This can be observed in the continuous downward slope of the viscosity *versus* shear rate curves.

## Conclusions

This work showed octofluorocyclopentene (OFCP) can be used as a key building block for the preparation of a diverse pool of segmented, vinyl-terminated monomers *via* base-promoted addition–elimination chemistry with bisphenolic or diols in overall high isolated yields. The use of these high molecular weight monomers underwent hydrosilylation polymerization with hydride-terminated polydimethylsiloxane (PDMS) oligomer to afford fluorinated silicones. Structural elucidation was confirmed through NMR spectroscopy confirmed their molecular weight and molar dispersity indices consistent with step-growth polymerization kinetics. Thermal analysis revealed that the segment incorporation did not affect the melt transition of the PDMS and their individual glass transition temperatures were subdued as a bulk co-polymer while retaining high thermal stability. Rheological analysis revealed complex temperature and shear dependent behavior of all copolymers. This behavior appears to be driven primarily by segment effects. The bulky and inflexible aryl segments tend to produce pseudoplastic-like material effects while the more mobile and flexible segments tend to look like structured materials with pronounced shear-thickening especially at elevated temperatures. Because of the simplicity of monomer synthesis using OFCP, an expanded study with longer aliphatic/aromatic segments can be envisioned with commercial bisphenols or diols. In addition, the adaptation of this preliminary study involving the incorporation of various molar feed ratios of monomers could produce terpolymer systems that would significantly influence shear thickening/thinning behavior. Therefore, a semi-fluorinated silicone-based system can be conceived with tailorable viscosity targeting a multitude of applications whereby operating temperatures under shear loads are important for fluid properties.

## Experimental

### Materials

Chemicals and solvents were purchased through commercial suppliers and used as received as reagent grade (>95% purity) unless specifically noted. Octafluorocyclopentene (OFCP) was purchased through SynQuest Laboratories. Hydride terminated polydimethylsiloxane (H-PDMS) with *M*_n_ of 1000 and *M*_n_ of 10 000 were acquired from Gelest. The synthesis of monomers M1 and M2 were carried out using a modified procedure from previously published work and structure elucidation was confirmed within this work.^27^M1 was originally reported as an oil but can be isolated as a low melting solid (mp 39–40 °C) upon allowing the as prepared oil to solidify into a crystalline solid after 96 h of storage at 20 °C.

### Instrumentation

^1^H, ^13^C{^1^H}, and ^19^F NMR spectra were recorded on a Jeol 500 MHz spectrometer. Chemical shifts were reported in parts per million (ppm), and the residual solvent peak was used as an internal reference: proton (chloroform *δ* 7.26), carbon (chloroform, C{D} triplet, *δ* 77.0 ppm), and fluorine (chloroform, CFCl_3_*δ* 0.00) was used as a reference. Data are reported as follows: chemical shift, multiplicity (s = singlet, m = multiplet at mid-point), coupling constants (Hz), and integration.

Differential scanning calorimetry (DSC) analyses were performed on a TA Q2500 utilizing aluminum hermetic pans. Analyses was carried out using a 5 °C min^−1^ temperature gradient under nitrogen. Thermogravimetric analyses (TGA) were performed on a TA Q5500 utilizing platinum pans at 10 °C min^−1^ temperature gradient in nitrogen. All thermal events were determined using TA Trios software graphical suite.

Gel permeation chromatography (GPC) data in tetrahydrofuran (HPLC grade) were collected using polystyrene standards (Polymer Labs Easical PS-2) by external calibration using a Waters 2695 Alliance System with a photodiode detector, whereby samples were eluted in series through Polymer Labs PLgel Mixed-D and Mixed-E columns.

Rheology constant strain oscillation frequency (rad s^−1^) sweep analysis was performed on a TA hybrid rheometer (HR30) using a 25 mm aluminum parallel plate geometry in an environmental test chamber maintained at specified temperature. After zeroing the gap polymer samples (*ca.* 1 g total mass) were dispensed on a lower 25 mm disposable aluminum plate (with drip channel). The geometry was them brought to trim gap (1003 μm) and excess sample was trimmed 360 deg. to leave a surface flush with the edge of plates (this sometimes required the adding of additional material). Chamber and sample were then brought to testing temperature and geometry brought to test gap (1000 μm). Frequency range for all tests was 0.6 rad s^−1^ to 600 rad s^−1^ at a constant strain of 30%, 50%, and 100%. Rheology events were determined using TA Trios software graphical suite.

Capillary melting point was determined using a Stuart SMP30 melting point apparatus at a heating rate of 5 °C min^−1^ using borosilicate capillary tubes.

Single crystal X-ray diffraction studies were carried out on a Rigaku XtaLAB synergy, Dualflex, HyPix3000 diffractometer equipped with a Cu K_α_ radiation source (*λ* = 1.542 Å). Suitable crystals were selected and mounted on a Cryoloop secured with Paratone-N oil. The crystal of interest was kept at a steady *T* = 100.0(3) K in a nitrogen gas stream during data collection. Crystal-to-detector distance was 40.6 mm using an exposure time of 0.2 seconds with a scan width of 0.50° for all crystals in this work. The data collection routine, unit cell refinement, and data processing were carried out with the program CrysAlisPro (version 43.91a) and scaled using an empirical absorption correction implemented in the SCALE 3 ABSPACK software program as well as a numerical absorption correction based on Gaussian integration over a multifaceted crystal model. The structure was solved using ShelXT 2018/2 (ref. 28) and refined using ShelXL 2019/3 (ref. 29) by least squares minimization *via* Olex2.^30^ The final refinement model involved anisotropic displacement parameters for non-hydrogen atoms and a riding model for all hydrogen atoms. Olex2, Mercury, and ORTEP3 were used for molecular graphics generation.

#### General procedure for synthesis of intermediates (I–V)

Intermediates I–V were prepared on a 15 g scale: triethylamine (3 equiv.) and 1,6-hexanediol (for I), 2,2,3,3,4,4,5,5-octafluorohexane-1,6-hexanediol (for II), hydroquinone (for III), bisphenol A (for IV), or bisphenol AF (for V) (2 equiv.) were combined in dimethylformamide (50 mL) and allowed to stir at room temperature. From a Schlenk tube maintained at *ca.* −20 °C, octafluorocyclopenene (2.5 equiv.) was transferred *via* syringe to the reaction vessel by dropwise addition. The reaction was monitored by ^19^F NMR until 100% conversion of the desired product was observed. The solution was vacuum filtered to remove salts and washed with dichloromethane (3 × 50 mL). The filtrate was then concentrated using rotary evaporation at 85 °C under high vacuum (20 mm Hg). The crude solid was reconstituted with 100% hexanes and eluted through a silica plug under vacuum. The filtrate was then concentrated using rotary evaporation, followed by high vacuum (5 mm Hg) to afford the desired compound.

##### 1,6-Bis((perfluorocyclopent-1-en-1-yl)oxy)hexane (I)

Colorless oil (94%). ^1^H NMR (CDCl_3_, 500 MHz) *δ* 4.39 (m, 4H), 1.81 (m, 4H), 1.49 (m, 4H); ^13^C{^1^H} NMR (126 MHz) *δ* 136.0 (m), 134.0 (m), 111.0 (m), 73.4, 28.9, 24.9; ^19^F NMR (CDCl_3_, 471 MHz) *δ* −114.9 (m, 4F), −116.3 (m, 4F), 129.6 (m, 4F), 162.5 (m, 2F).

##### 2,2′-((2,2,3,3,4,4,5,5-Octafluorohexane-1,6-diyl)bis(oxy))bis(1,3,3,4,4,5,5-heptafluorocyclopent-1-ene) (II)

Turbid yellow oil (94%). ^1^H NMR (CDCl_3_, 500 MHz) *δ* 4.79 (m, 4H); ^13^C{^1^H} NMR (126 MHz) *δ* 137.6 (m), 134.4 (m), 110.8(m), 67.6 (m); ^19^F NMR (CDCl_3_, 471 MHz) *δ* −115.6 (m, 4F), −115.7 (m, 8F), −120.7 (m, 4F), −123.4 (m, 4F), −129.5 (m, 4F), −156.8 (m, 2F).

##### 1,4-Bis((perfluorocyclopent-1-en-1-yl)oxy)benzene (III)

White crystalline solid (49%). ^1^H NMR (CDCl_3_, 500 MHz) *δ* 7.23 (m, 4H); ^13^C{^1^H} NMR (126 MHz) *δ* 151.6, 145.0 (m), 120.5, 111.2 (m); ^19^F NMR (CDCl_3_, 471 MHz) *δ* −115.4 (m, 4F), 115.7 (m, 4F), 129.5 (m, 4F), 149.0 (m, 2F).

##### 4,4′-(Propane-2,2-diyl)bis(((perfluorocyclopent-1-en-1-yl)oxy)benzene) (IV)

Viscous yellow oil (65%). ^1^H NMR (CDCl_3_, 500 MHz) *δ* 7.23 (m, *J* = 8 Hz, 4H), 7.08 (m, *J* = 6 Hz, 4H), 1.69 (s, 6H); ^13^C{^1^H} NMR (126 MHz) *δ* 152.9, 148.7, 134.5 (m), 128.4, 118.2, 110.6 (m), 42.7, 30.8; ^19^F NMR (CDCl_3_, 471 MHz) *δ* −115.5 (m, 4F), −115.5 (m, 4F), −129.5 (m, 4F), −149.6 (m, 2F).

##### 4,4′-(Perfluoropropane-2,2-diyl)bis(((perfluorocyclopent-1-en-1-yl)oxy)benzene) (V)

Viscous yellow oil (92%). ^1^H NMR (CDCl_3_, 500 MHz) *δ* 7.44 (m, *J* = 9 Hz, 4H), 7.19 (m, *J* = 9 Hz, 4H); ^13^C{^1^H} NMR (126 MHz) *δ* 154.1, 136.2 (m), 131.3, 118.3, 110.1 (m), 76.0 (m); ^19^F NMR (CDCl_3_, 471 MHz) *δ* −64.0 (m, 6F) −115.5 (m, 4F), 115.8 (m, 4F), −129.6 (m, 4F), −146.8 (m, 2F).

#### General procedure for the synthesis of monomers M3–M7

Monomers M3–M7 were prepared on a 5-gram scale: cesium carbonate (4 equiv.), eugenol (2.05 equiv.), and intermediate I–V(1 equiv.) were combined in dimethylformamide (50 mL) and allowed to stir for 24–72 h at room temperature. The reaction was monitored by ^19^F NMR until 100% conversion of the desired product was observed. The solution was vacuum filtered to remove carbonate salts and washed with dichloromethane (3 × 50 mL). The filtrate was then concentrated using rotary evaporation at 85 °C under high vacuum (20 mm Hg). The crude solid was reconstituted with ethyl acetate/hexanes (5 : 95 vol/vol) and eluted through a silica plug under vacuum. The filtrate was then concentrated using rotary evaporation, followed by high vacuum (5 mm Hg) to afford the desired compound.

##### 1,6-Bis((2-(4-allyl-2-methoxyphenoxy)-3,3,4,4,5,5-hexafluorocyclopent-1-en-1-yl)oxy)hexane (M3)

Yellow oil (96%). ^1^H NMR (CDCl_3_, 500 MHz) *δ* 6.99 (m, *J* = 8.5 Hz, 4H), 6.77 (m, *J* = 8.5 Hz, 4H), 5.93 (m, 2H), 5.08 (m, 4H), 4.23 (m, 4H), 3.81 (s, 6H), 3.36 (m, 4H), 1.54 (m, 4H), 1.24 (m, 4H); ^13^C{^1^H} NMR (126 MHz) *δ* 150.3, 142.4, 138.9, 136.9, 120.7, 119.1, 116.3, 113.0, 112.0–109.0 (unresolved multiplicity), 72.6, 55.9, 40.0, 29.1, 24.8 (m); ^19^F NMR (CDCl_3_, 471 MHz) *δ* −112.0 (m, 4F), −114.3 (m, 4F), −129.9 (m, 4F).

##### 4,4′-((((2,2,3,3,4,4,5,5-Octafluorohexane-1,6-diyl)bis(oxy))bis(3,3,4,4,5,5-hexafluorocyclopent-1-ene-2,1-diyl))bis(oxy))bis(1-allyl-3-methoxybenzene) (M4)

Pale yellow oil (80%). ^1^H NMR (CDCl_3_, 500 MHz) *δ* 7.09 (m, *J* = 8.5 Hz, 2H), 6.76 (m, *J* = 8.3 Hz, 4H), 5.95 (m, 2H), 5.08 (m, 4H), 4.80 (m, 4H), 3.82 (s, 6F), 3.37 (m, 4F); ^13^C{^1^H} NMR (126 MHz) *δ* 150.8, 140.0, 139.7, 136.7 (m), 130.0–109.0 (unresolved multiplicity), 120.9, 116.4, 112.9, 67.6 (m), 55.5 (m), 40.0 (m); ^19^F NMR (CDCl_3_, 471 MHz) *δ* −112.5 (m, 4F), −114.7 (m, 4F), −121.0 (m, 4F), 123.7 (m, 4F), 129.8 (m, 4F).

##### 1,4-Bis((2-(4-allyl-2-methoxyphenoxy)-3,3,4,4,5,5-hexafluorocyclopent-1-en-1-yl)oxy)benzene (M5)

White crystalline solid (83%). Mp 96–98 °C; ^1^H NMR (CDCl_3_, 500 MHz) *δ* 6.68 (m, 2H), 6.52–6.74 (overlapping m, 8H), 5.83 (m, 2H), 5.02 (m, 4H), 3.67 (s, 6H), 3.22 (m, 4H); ^13^C{^1^H} NMR (126 MHz) *δ* 151.4, 149.9, 140.5, 139.1, 136.8, 120.3, 119.9, 116.4, 116.7112.5, 55.6, 39.9; ^19^F NMR (CDCl_3_, 471 MHz) *δ* −113.2 (m, 4F), −115.5 (m, 4F), −130.2 (m, 4F).

##### 4,4′-((((Propane-2,2-diylbis(4,1-phenylene))bis(oxy))bis(3,3,4,4,5,5-hexafluorocyclopent-1-ene-2,1-diyl))bis(oxy))bis(1-allyl-3-methoxybenzene) (M6)

Viscous yellow oil (92%). ^1^H NMR (CDCl_3_, 500 MHz) *δ* 6.95 (m, *J* = 8.5 Hz, 4H), 6.64, (m, *J* = 8.5 Hz, 2H), 6.54 (m, *J* = 8.5 Hz, 6H), 6.42 (m, 2H), 5.83 (m, 2H), 5.02 (m, 4H), 3.70 (s, 6H), 3.23 (m, 4H), 1.56 (m, 4H), 1.25 (m, 1H); ^13^C{^1^H} NMR (126 MHz) *δ* 153.5, 150.0, 146.0, 140.5, 138.8, 136.9, 127.5, 120.0, 116.2, 115.3, 112.5, 60.5, 55.7, 41.9, 40.0, 31.0, 21.1, 14.3; ^19^F NMR (CDCl_3_, 471 MHz) *δ* −113.3 (m, 4F), −115.5 (m, 4F), −130.2 (m, 4F).

##### 4,4′-(((((Perfluoropropane-2,2-diyl)bis(4,1-phenylene))bis(oxy))bis(3,3,4,4,5,5-hexafluorocyclo-pent-1-ene-2,1-diyl))bis(oxy))bis(1-allyl-3-methoxybenzene) (M7)

Colorless viscous oil (93%). ^1^H NMR (CDCl_3_, 500 MHz) *δ* 7.09 (m, 4H), 6.57–6.53 (overlapping m, 8H), 6.42 (m, 2H), 5.80 (m, 2H), 5.02 (m, 4H), 3.71 (s, 6H) 3.21 (m, 4H); ^13^C{^1^H} NMR (126 MHz) *δ* 156.0, 150.1, 140.0, 139.4, 136.6, 131.3, 128.3, 120.2, 116.3, 115.0, 112.4, 55.7, 39.9; ^19^F NMR (CDCl_3_, 471 MHz) *δ* −64.0 (m, 6F), −113.2 (m, 4F), −116.2 (m, 4F), −130.3 (m, 4F).

#### Synthesis of copolymers P1–P7

H-PDMS (1000 g per mol avg molecular weight, 1 equiv.) was added to M1 or M2 (1 equiv.) and then Karstedt's Pt catalyst (5 wt% platinum divinyltetramethyldisiloxane complex in xylene, 0.05 equiv.) was added. The solution was vortexed for 5 min and then allowed to set at room temperature for 24 h. The copolymers were used without further purification affording a transparent, brown liquid. The same procedure was used to prepare P3–P7 with the corresponding monomers M3–M7 except a minimal amount of toluene was added to facilitate miscibility before adding the Pt catalyst.

## Conflicts of interest

The authors declare no competing financial interest.

## Supplementary Material

RA-015-D5RA03087K-s001

RA-015-D5RA03087K-s002

## Data Availability

The data supporting this article have been included as part of the ESI.†^1^H, ^19^F, ^13^C NMR for monomers M1–M7. ^1^H and ^19^F NMR for copolymers P1–P7. Tabulated *M*_n_ calculations from end-group analysis using ^1^H NMR. DSC for monomers M1–M7 and copolymers P1–P7. TGA data for copolymers P1–P7. Supplementary crystallographic data for the single crystal X-ray structure can be obtained free of charge *via*www.ccdc.cam.ac.uk/data_request/cif or by emailing da-ta_request@ccdc.cam.ac.uk, or by contacting the Cambridge Crystallographic Data Centre, 12 Union Road, Cambridge CB2 1EZ, UK, fax: +44-1223-336033 with reference to CCDC 2440261 (M1) and 2440277 (M3).
