# Supplementary material for: Modularly prepared segmented fluorinated silicones and their anomalous microstructure dependent flow behavior
Source: RSC Adv. 2025 Jun 24;15(27):21429–38. doi: 10.1039/d5ra03087k (PMC12186765; doi:10.1039/d5ra03087k)
Supplement: RA-015-D5RA03087K-s001 [file RA-015-D5RA03087K-s001.pdf]

Supplementary Information (SI) for RSC Advances.
This journal is © The Royal Society of Chemistry 2025

SUPPORTING INFORMATION

Modularly Prepared Segmented Fluorinated Silicones and their Anomalous Microstructure Dependent Flow Behavior

*Analise C. Migliaccio, Nathan J. Weeks, Scott T. Iacono**

[a]Department of Chemistry and Chemistry Research Center, Laboratories for Advanced Materials, United States Air Force Academy, Colorado Springs, Colorado (USA)

*Correspondence e-mail: scott.iacono@afacademy.af.edu

Table of Contents

NMR Data....S2
End-Group Molecular Weight Calculations....S46
DSC and TGA Thermal Data....S47
GPC Molecular Weight Data....S50

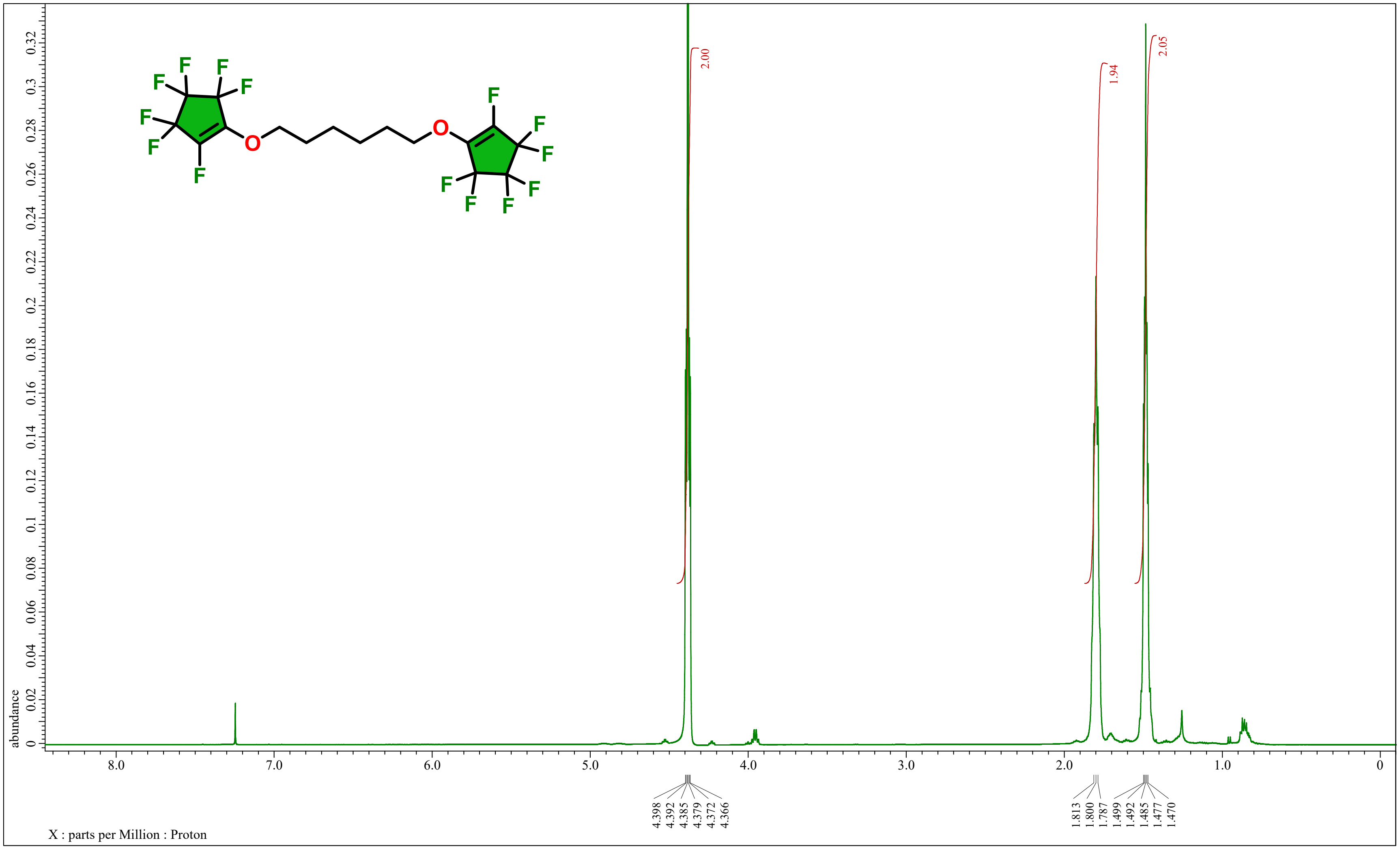

Figure S1. ^{1}H NMR of **I**.

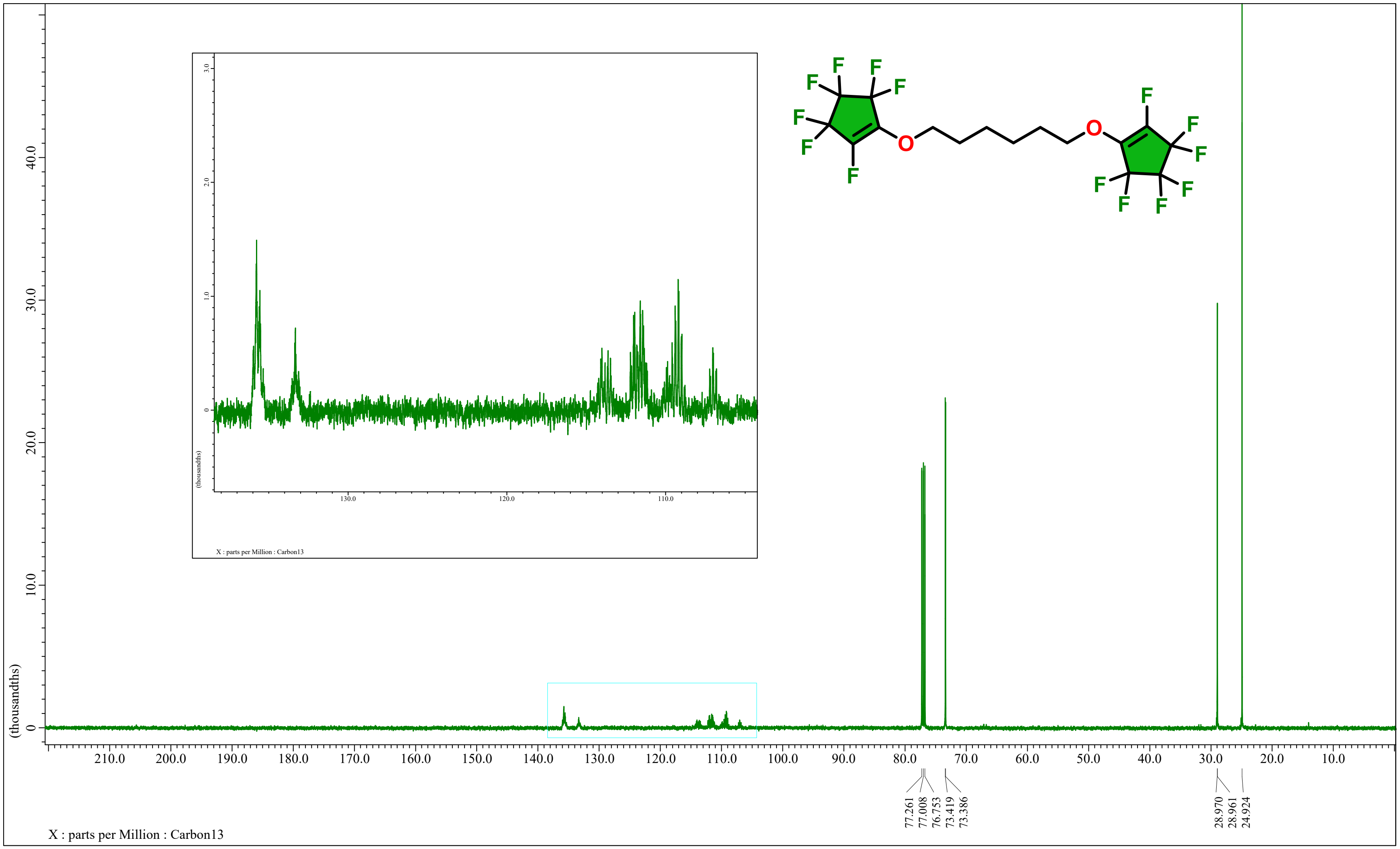

Figure S2. ^{13}C NMR **I**.

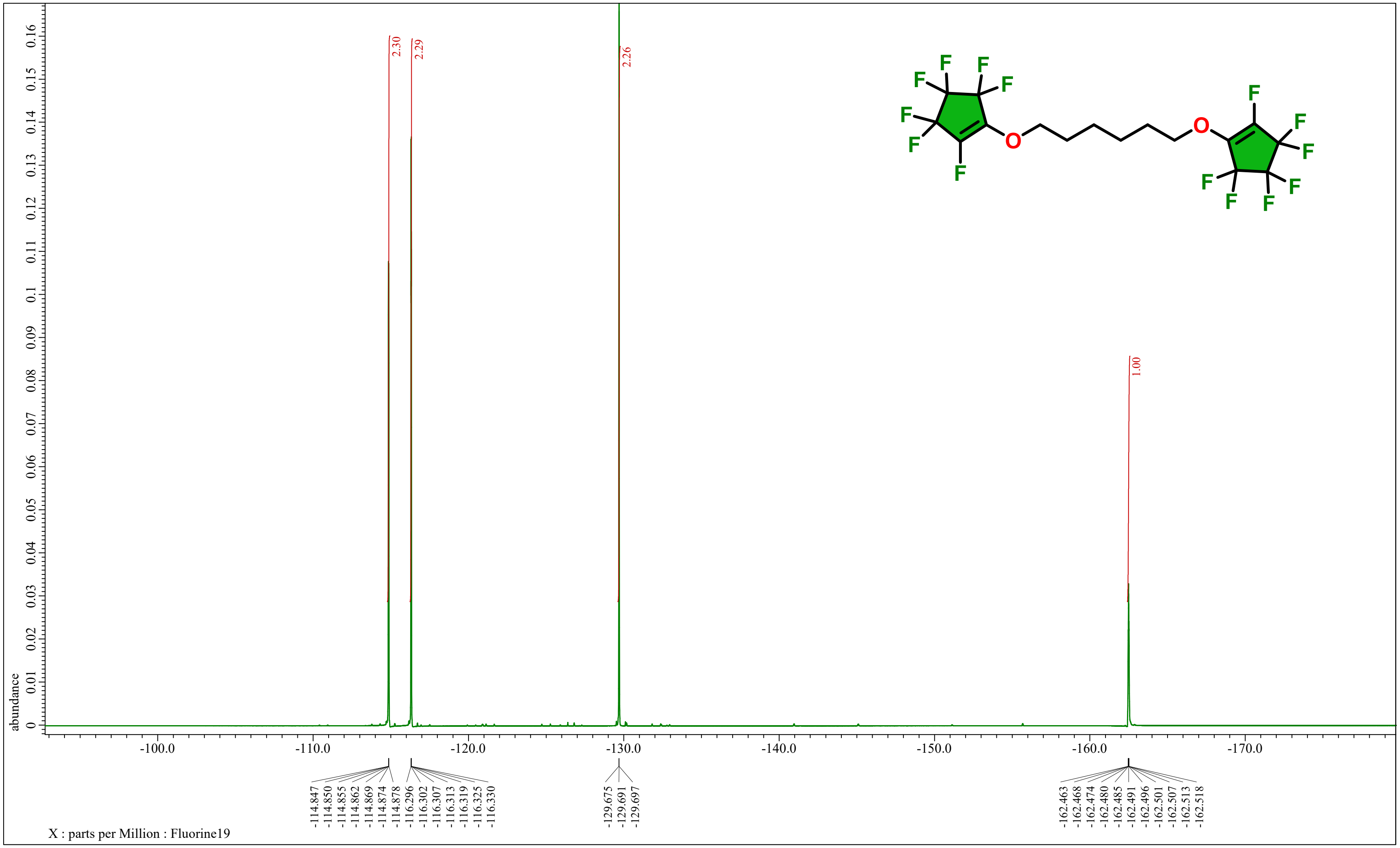

Figure S3. ^{19}F NMR **I**.

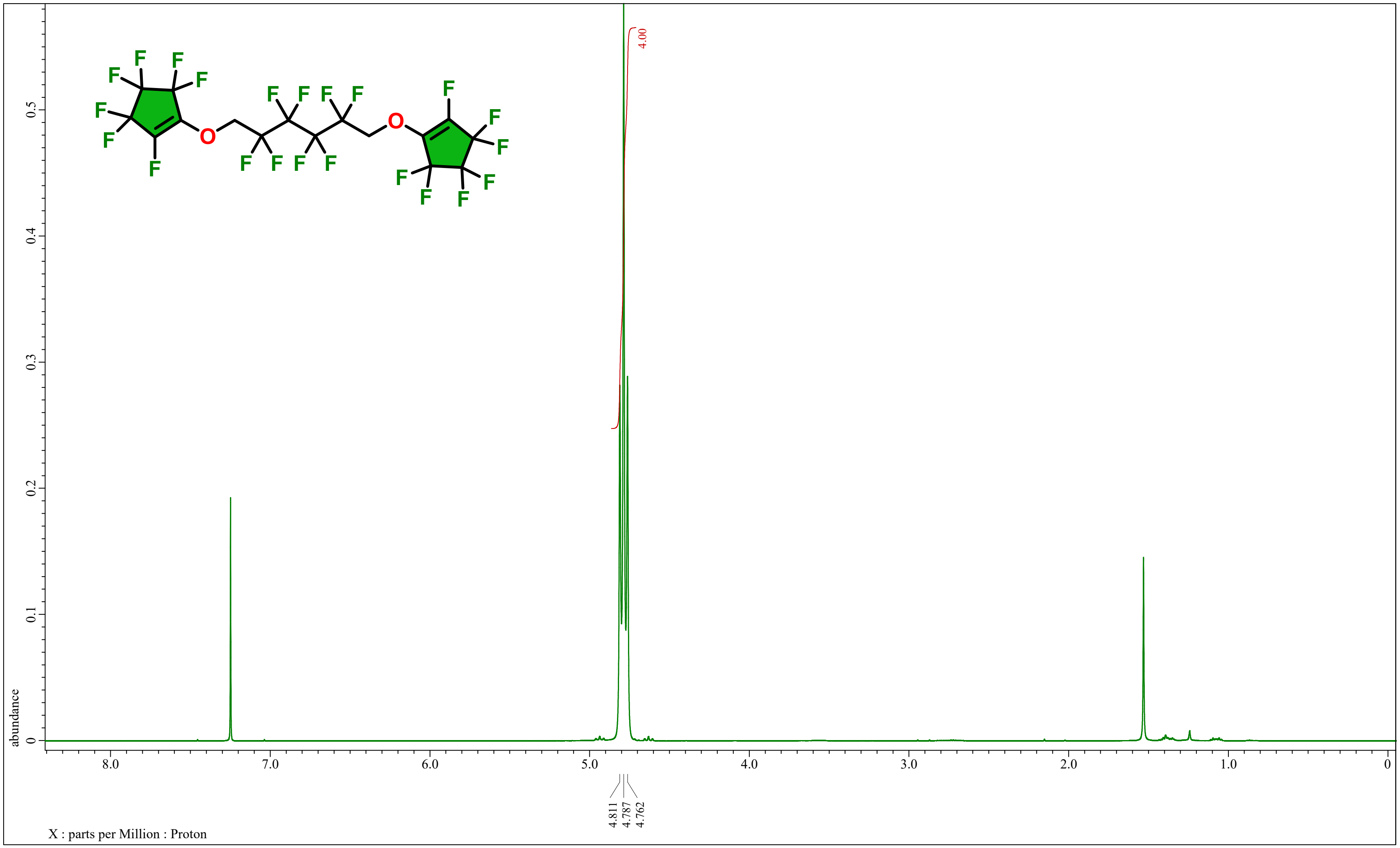

Figure S4. ^{1}H NMR of **II**.

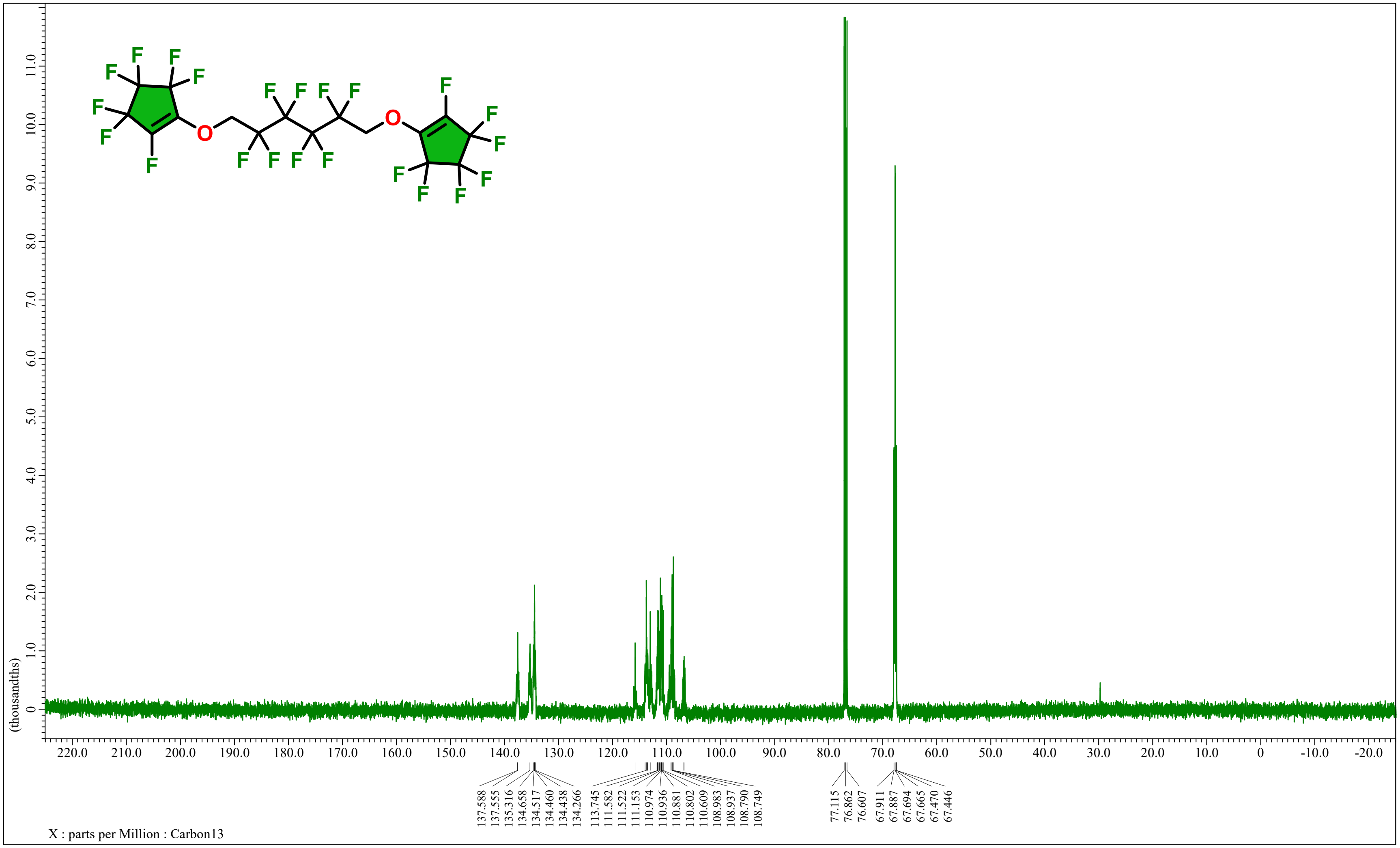

Figure S5. ^{13}C NMR **II**.

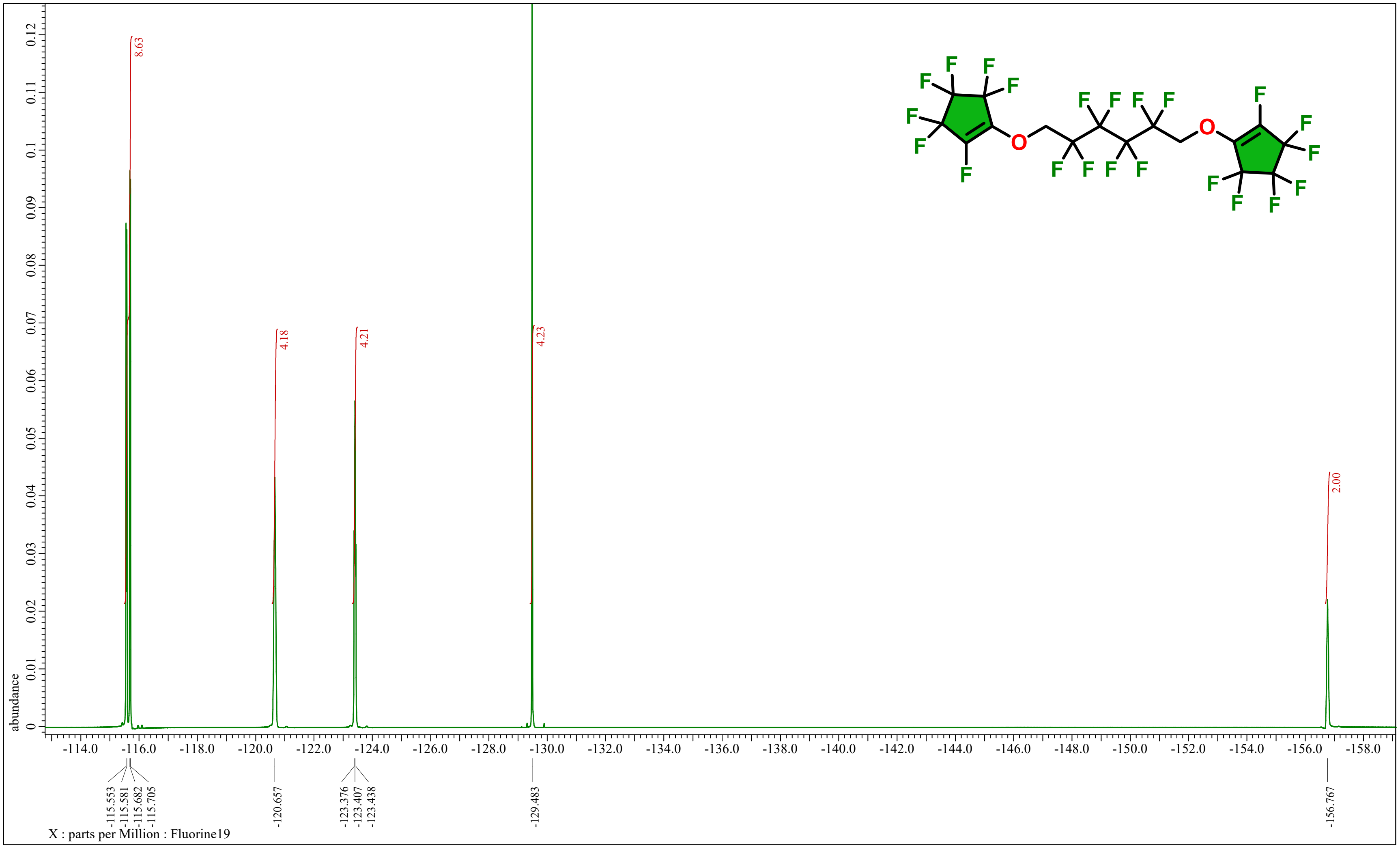

Figure S6. ^{19}F NMR **II**.

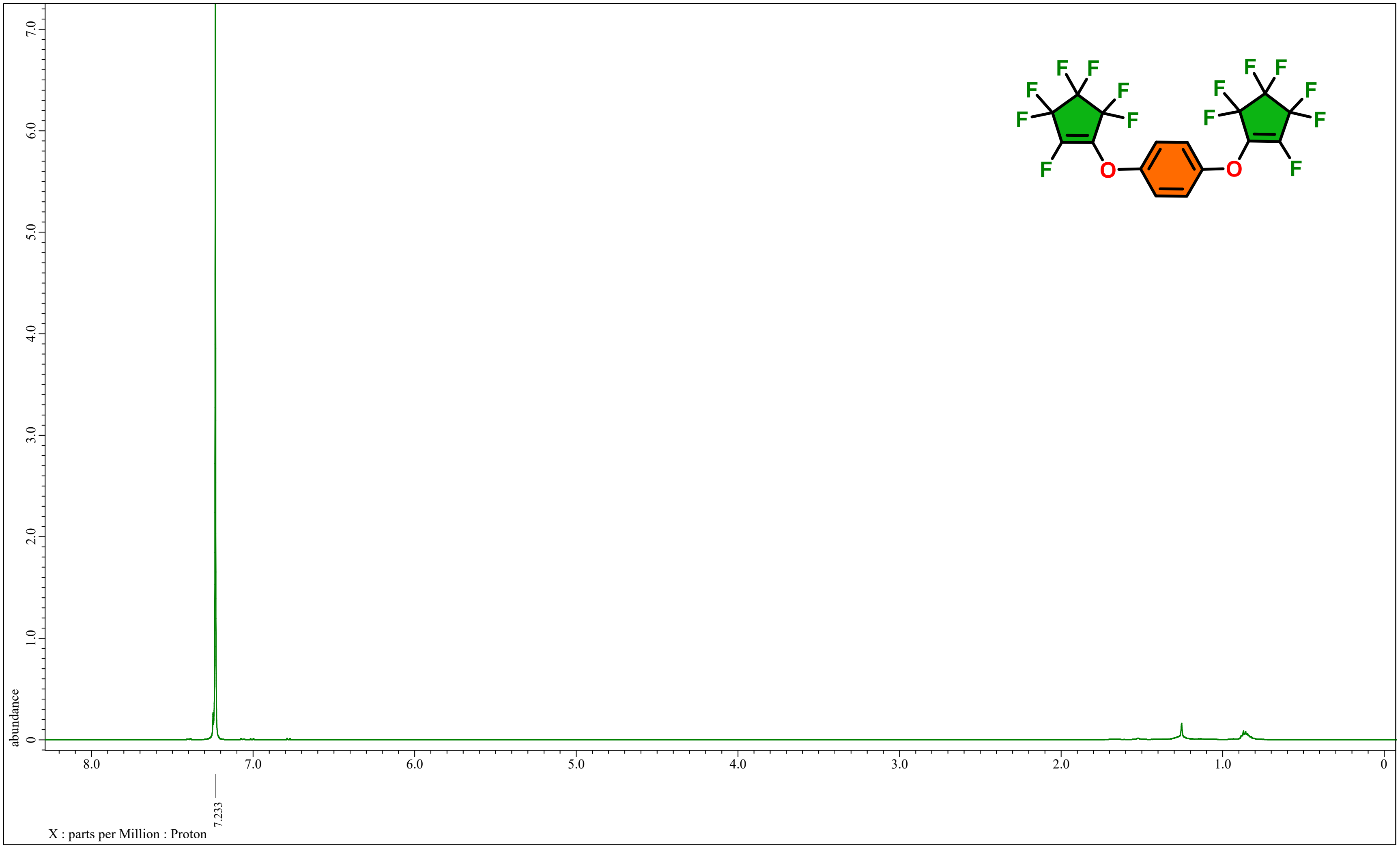

Figure S7. ^{1}H NMR of **III**.

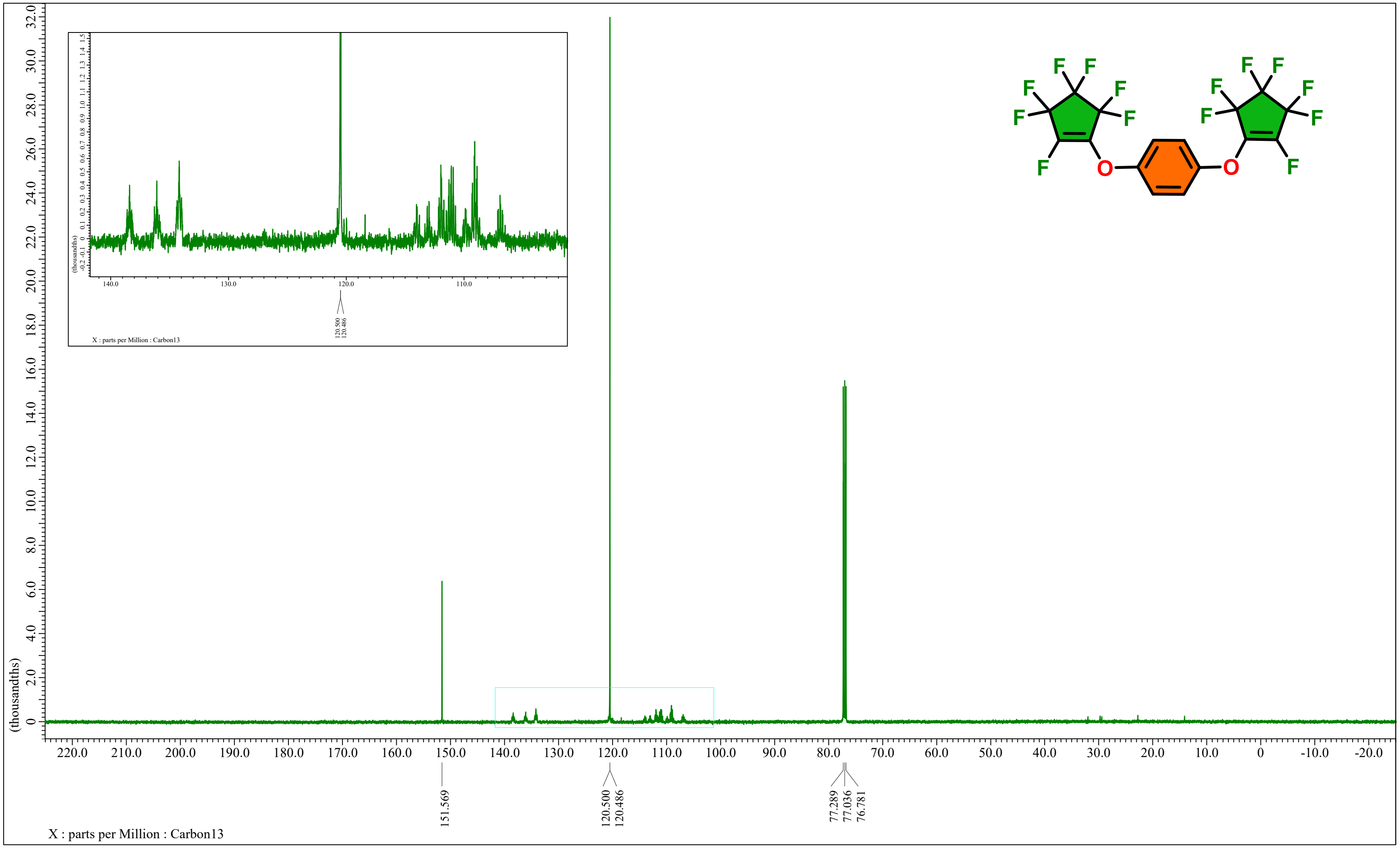

Figure S8. ^{13}C NMR **III**.

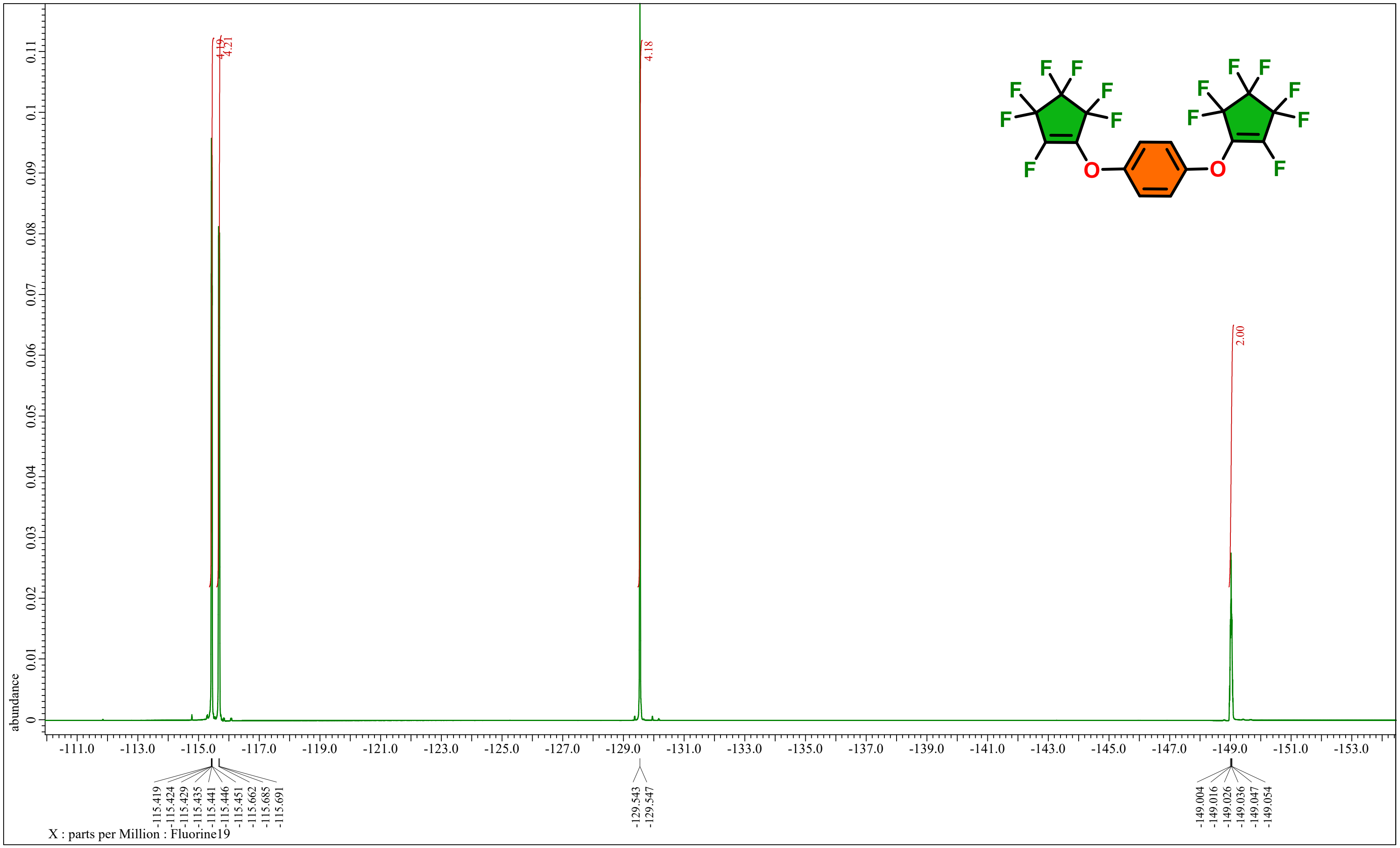

Figure S9. ^{19}F NMR **III**.

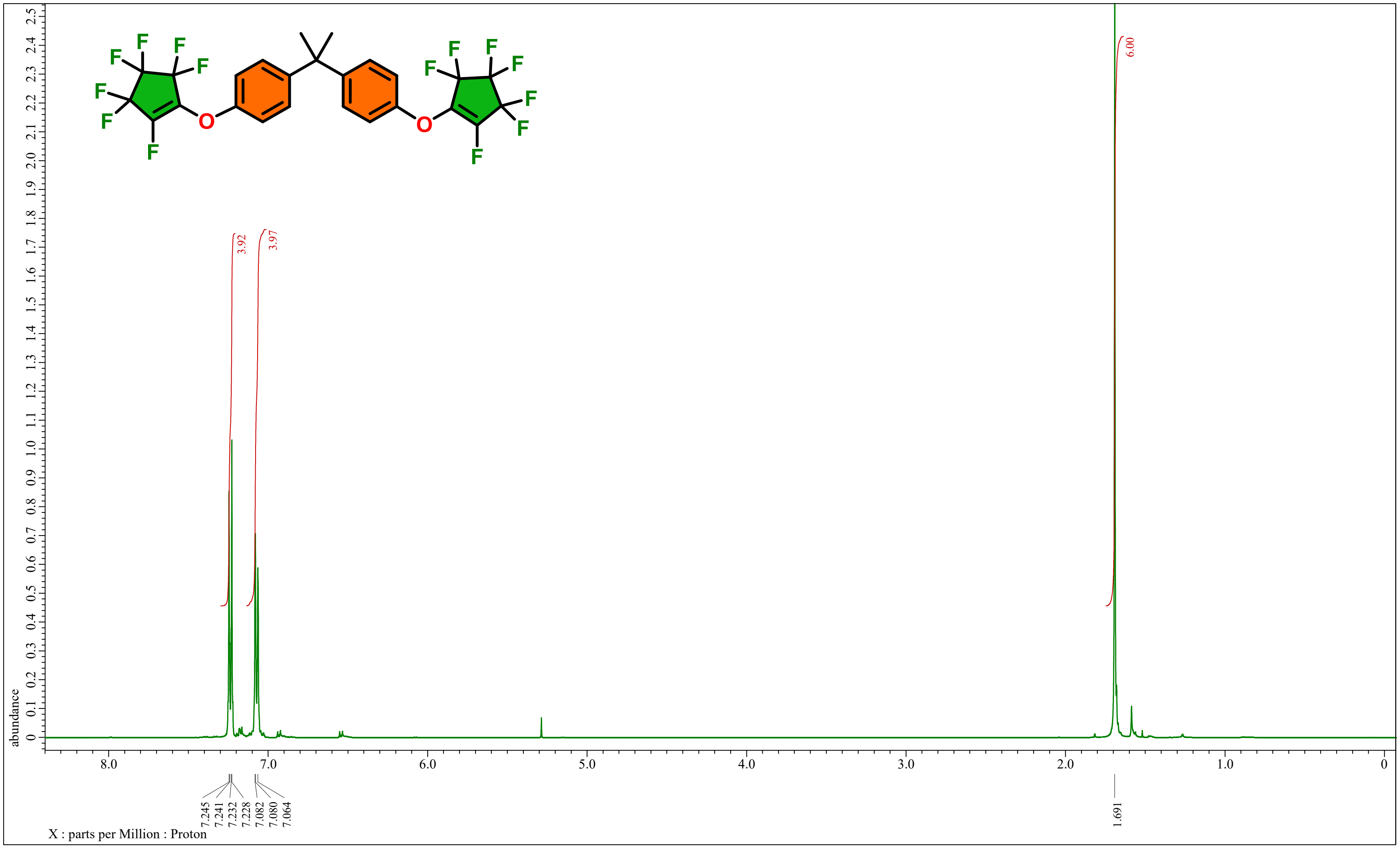

Figure S10. ^{1}H NMR of **IV**.

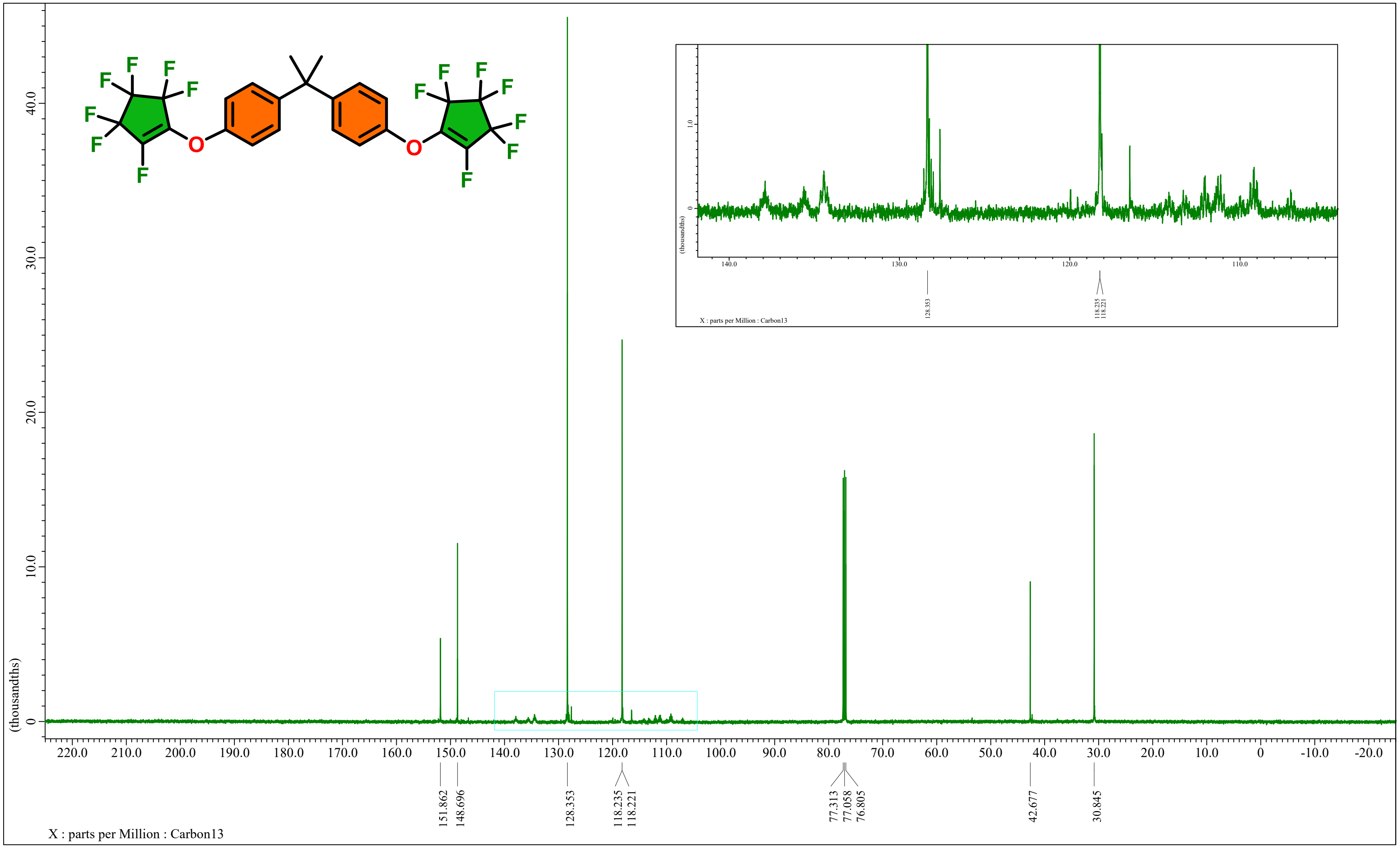

Figure S11. ^{13}C NMR **IV**.

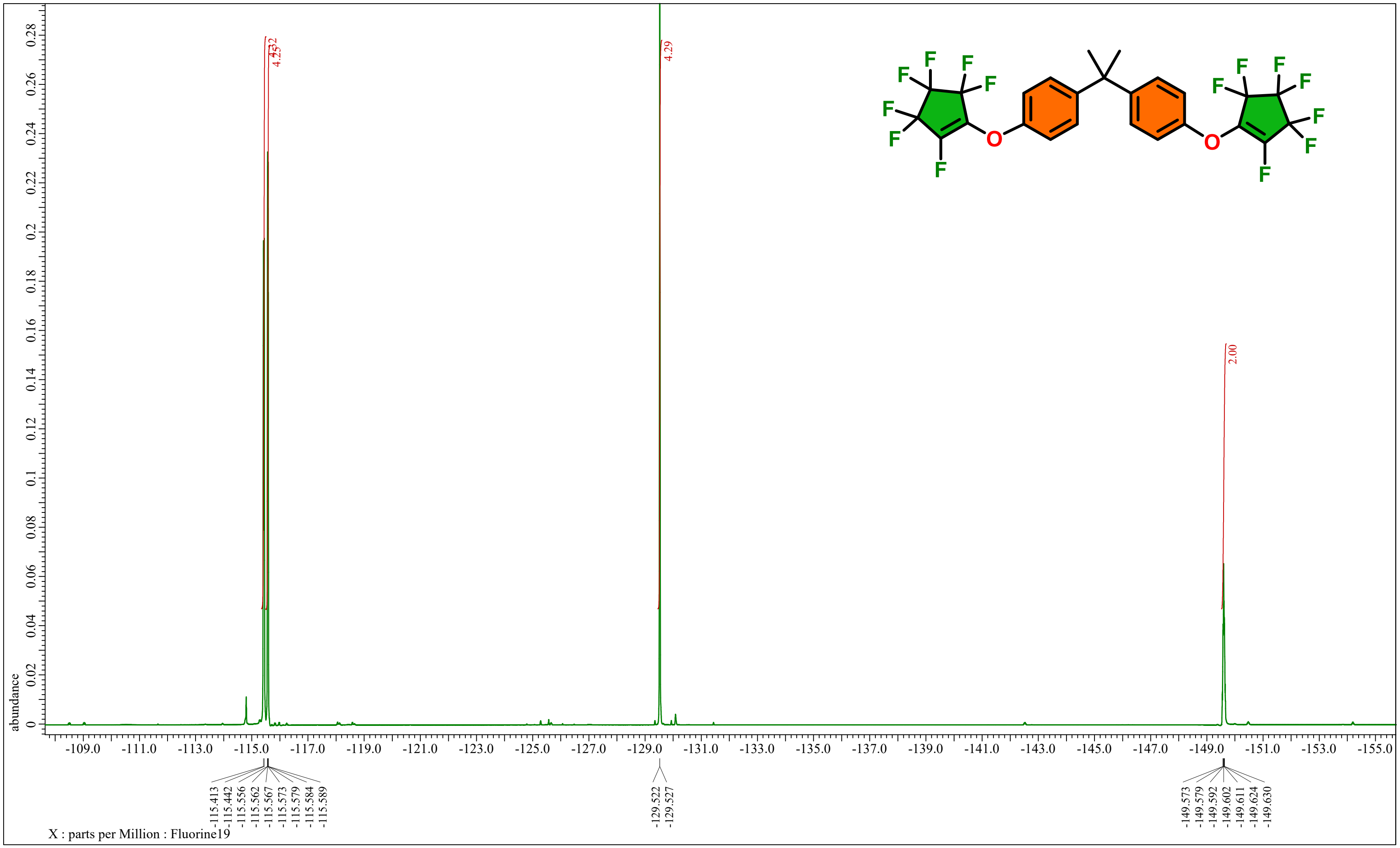

Figure S12. ^{19}F NMR **IV**.

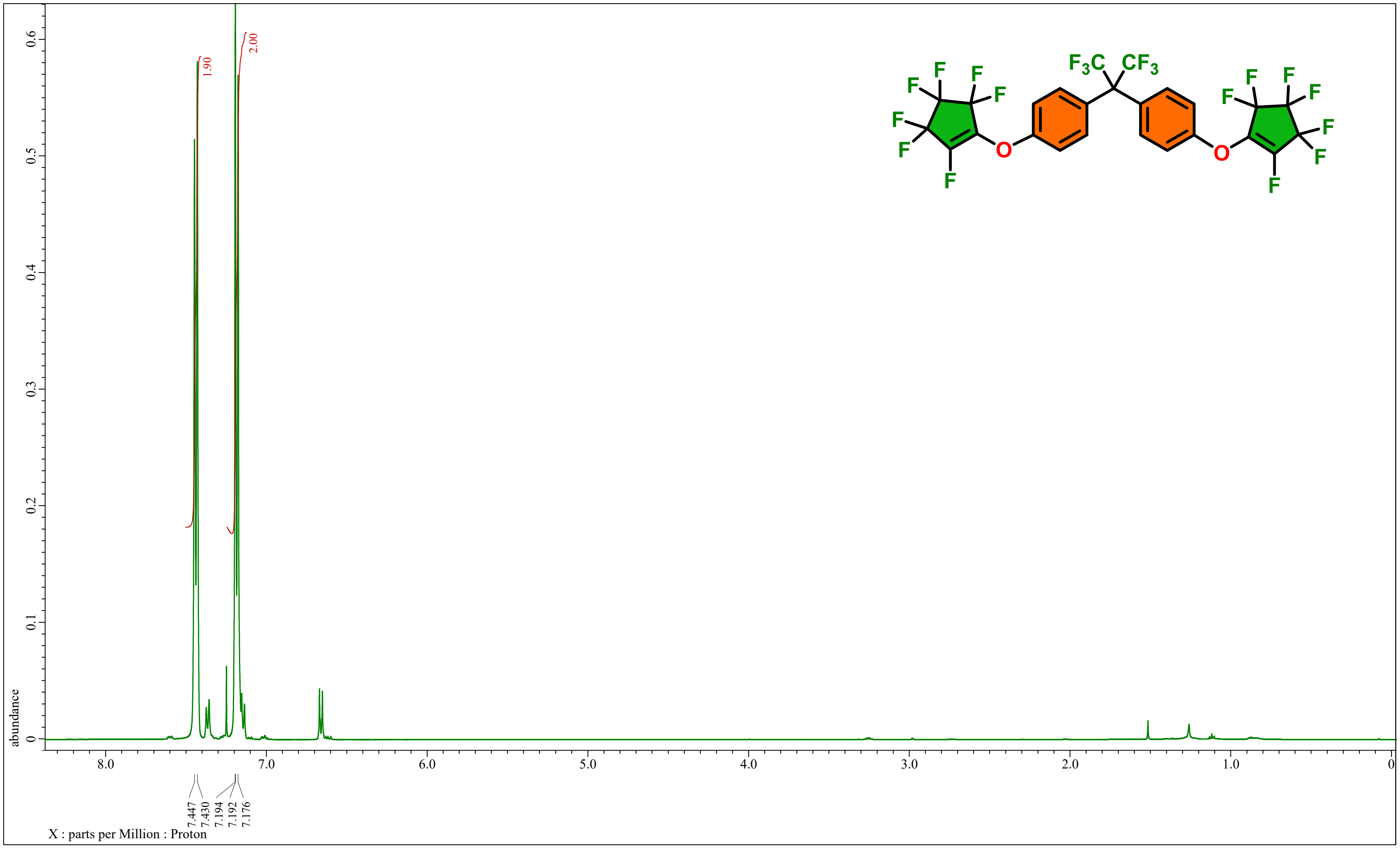

Figure S13. ^{1}H NMR of **V**.

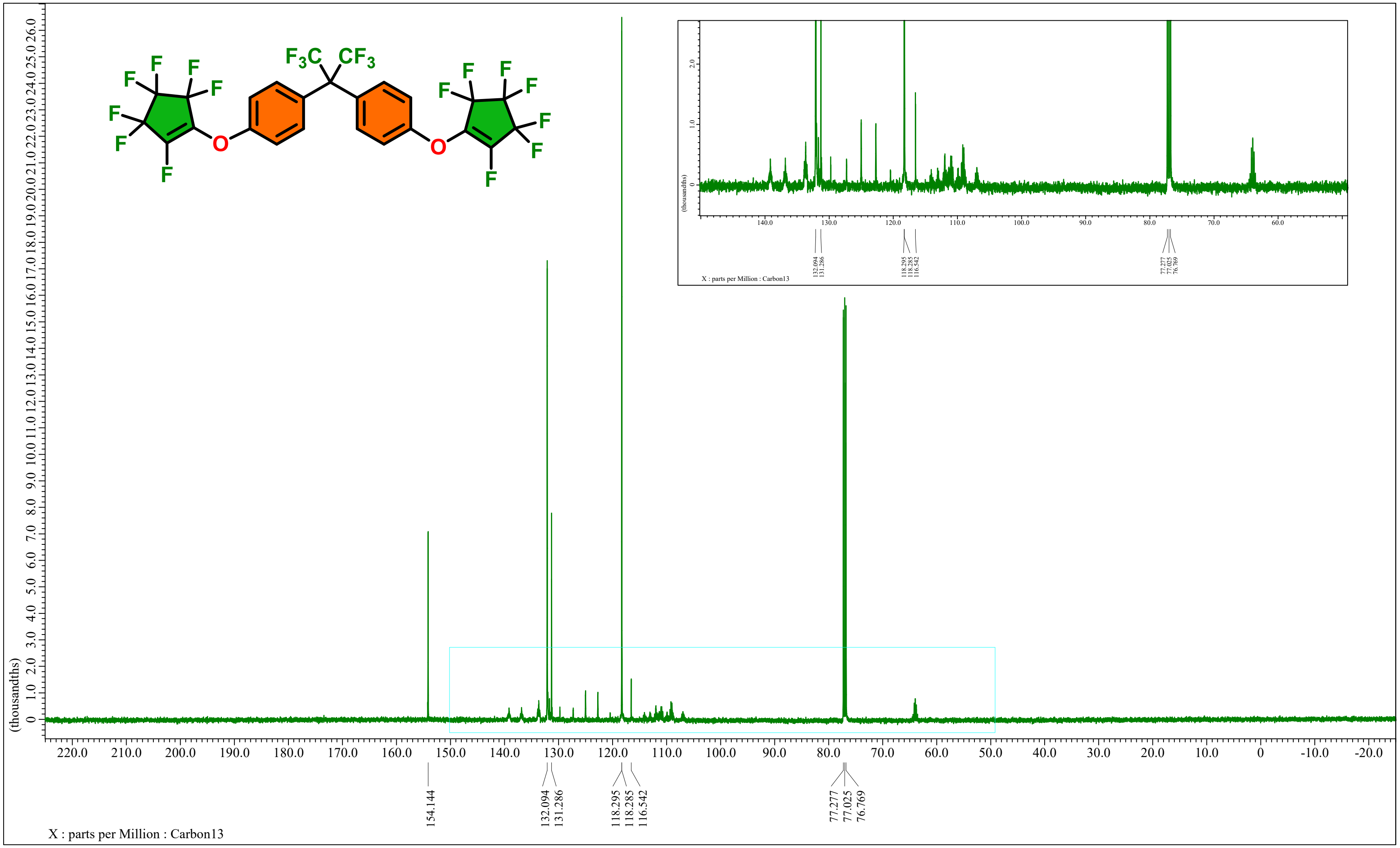

Figure S14. ^{13}C NMR **V**.

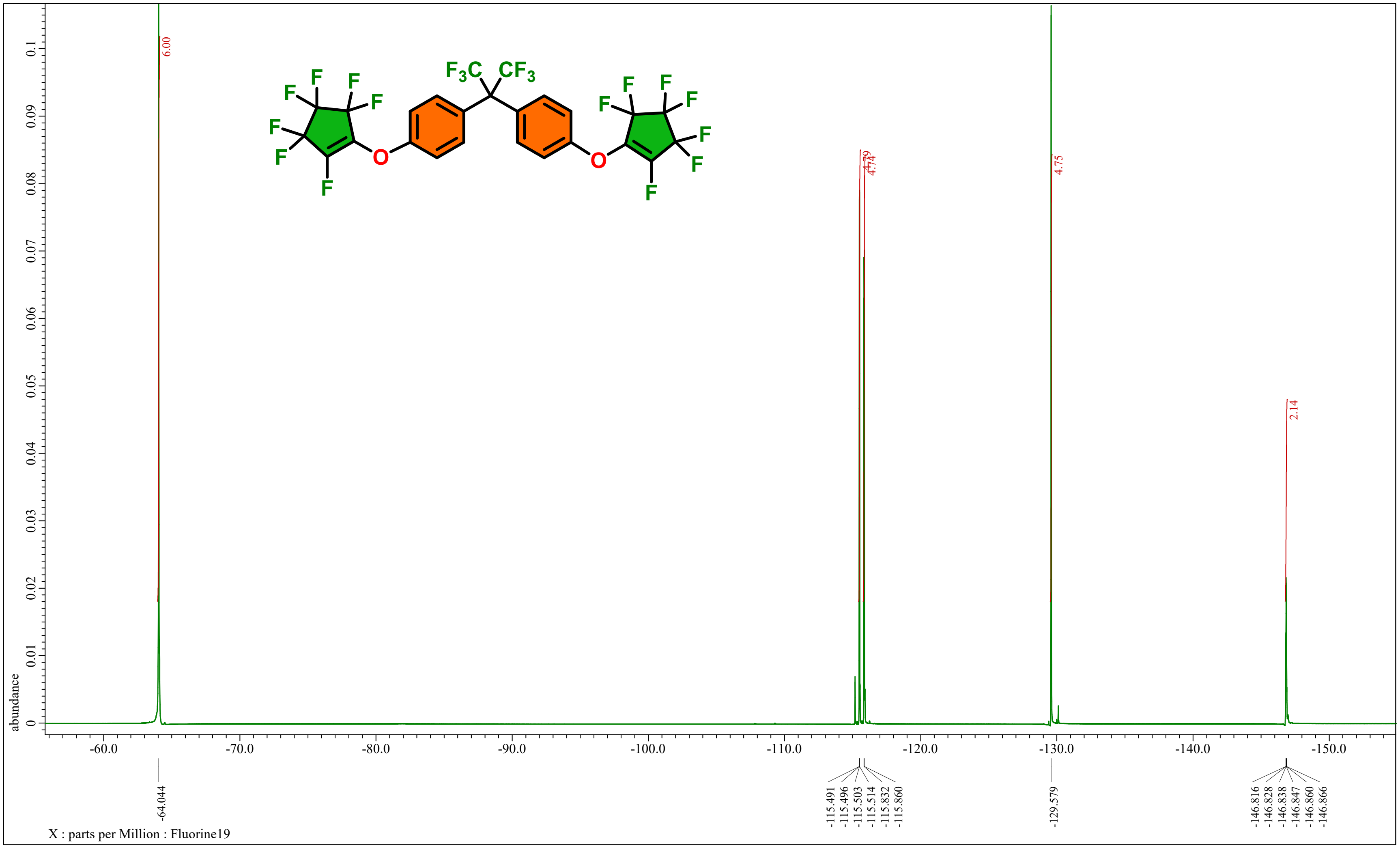

Figure S15. ^{19}F NMR **V**.

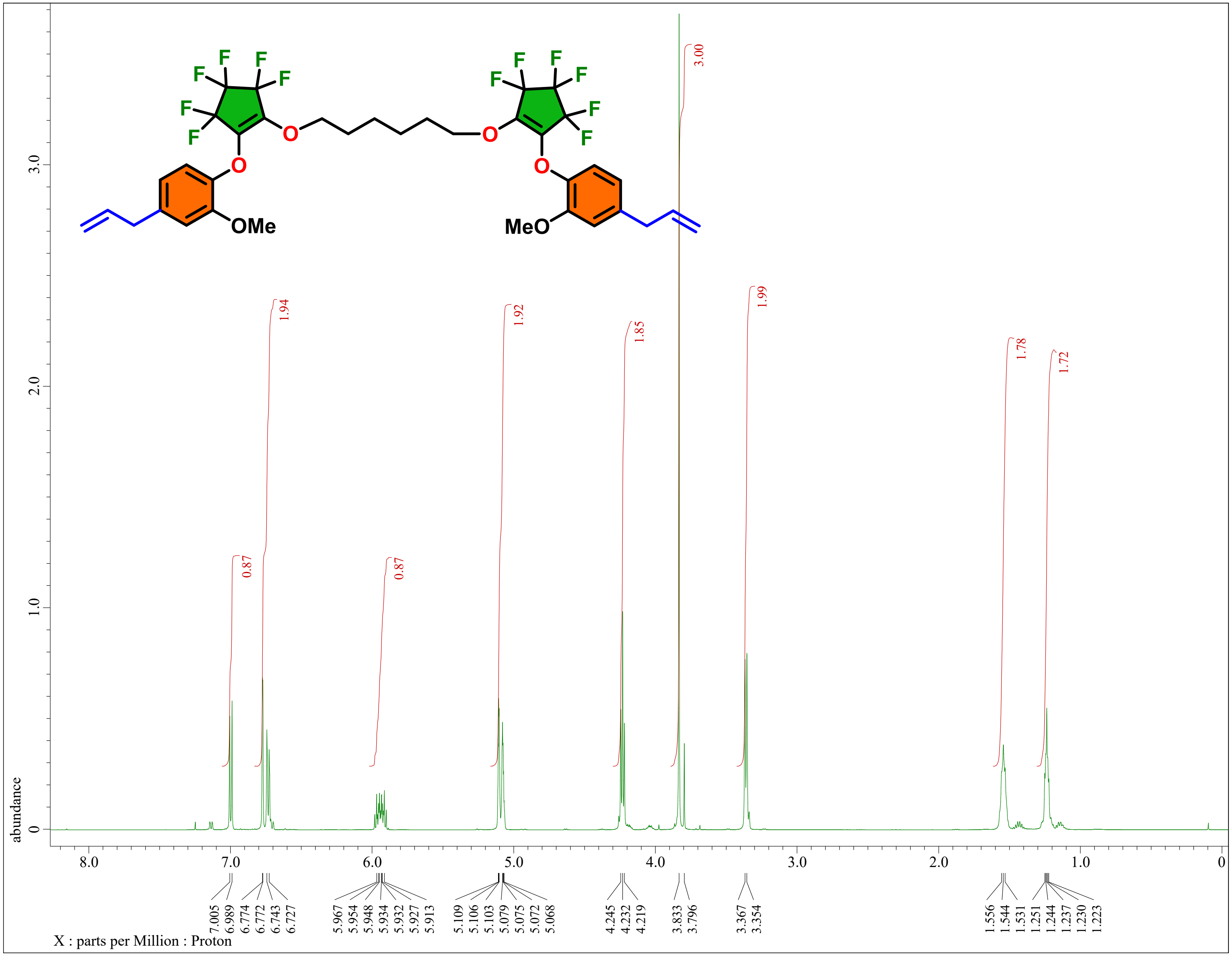

Figure S16. ^{1}H NMR of **M3**.

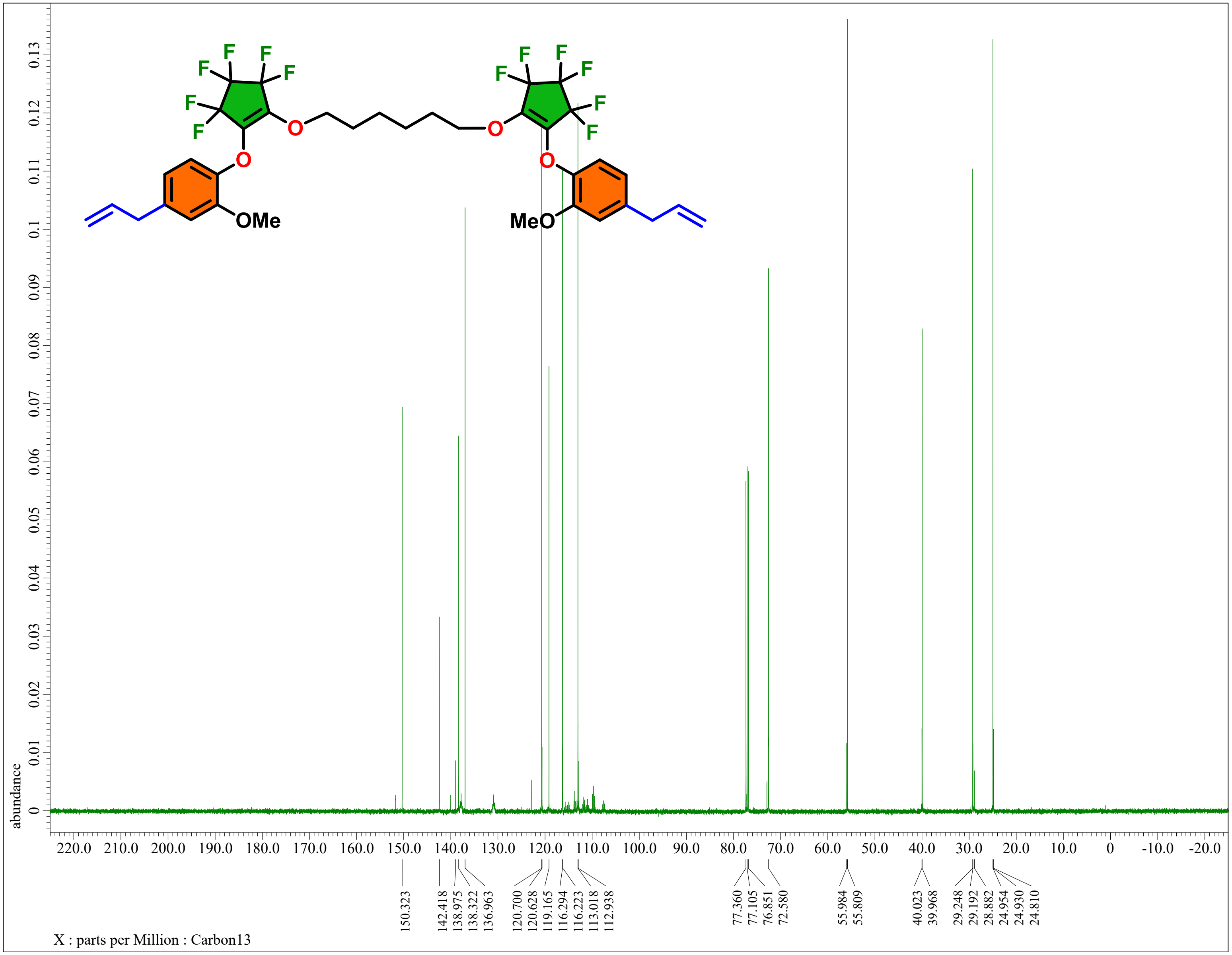

Figure S17. ^{13}C NMR **M3**.

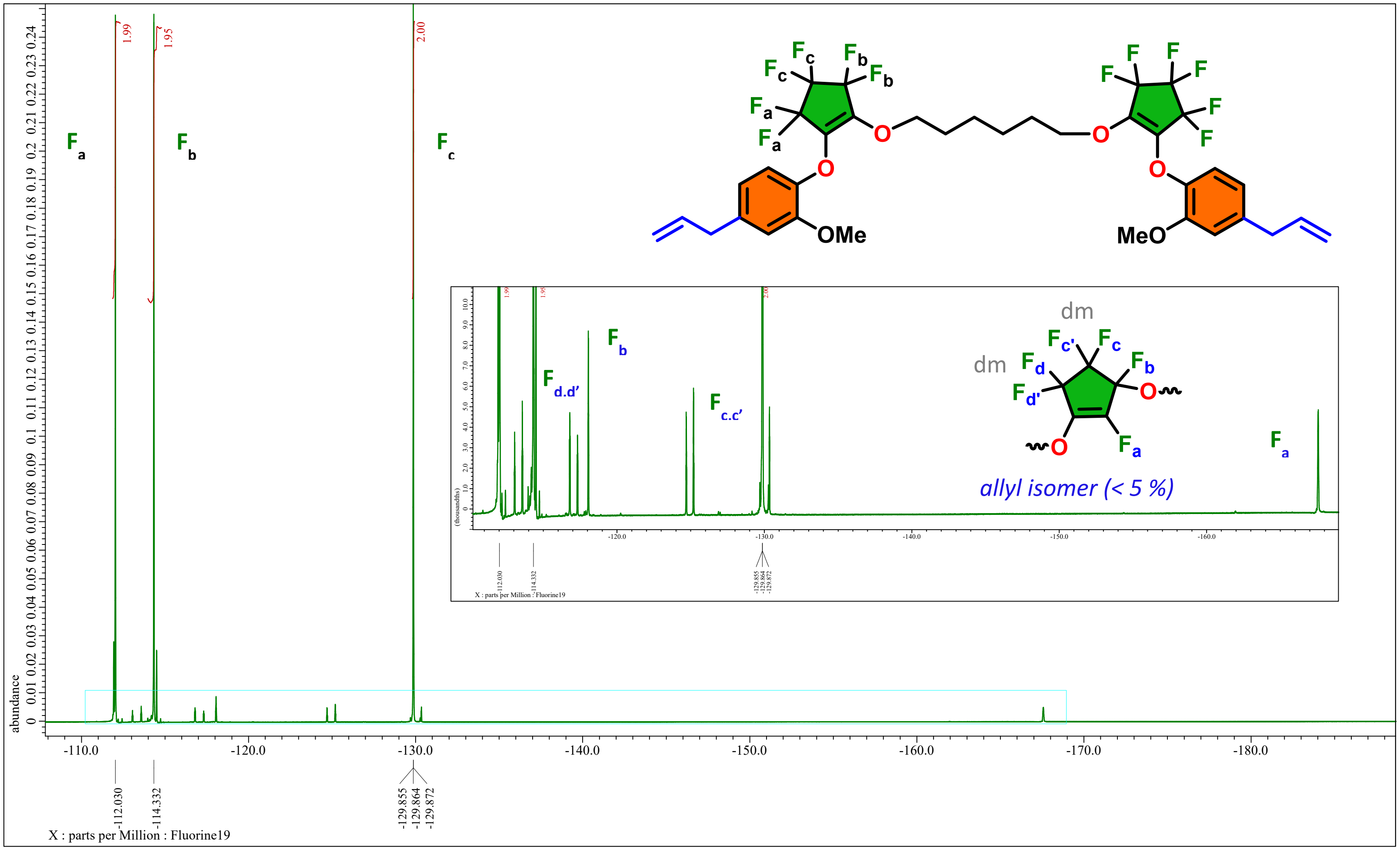

Figure S18. ^{19}F NMR of **M3** and the allyl isomer.

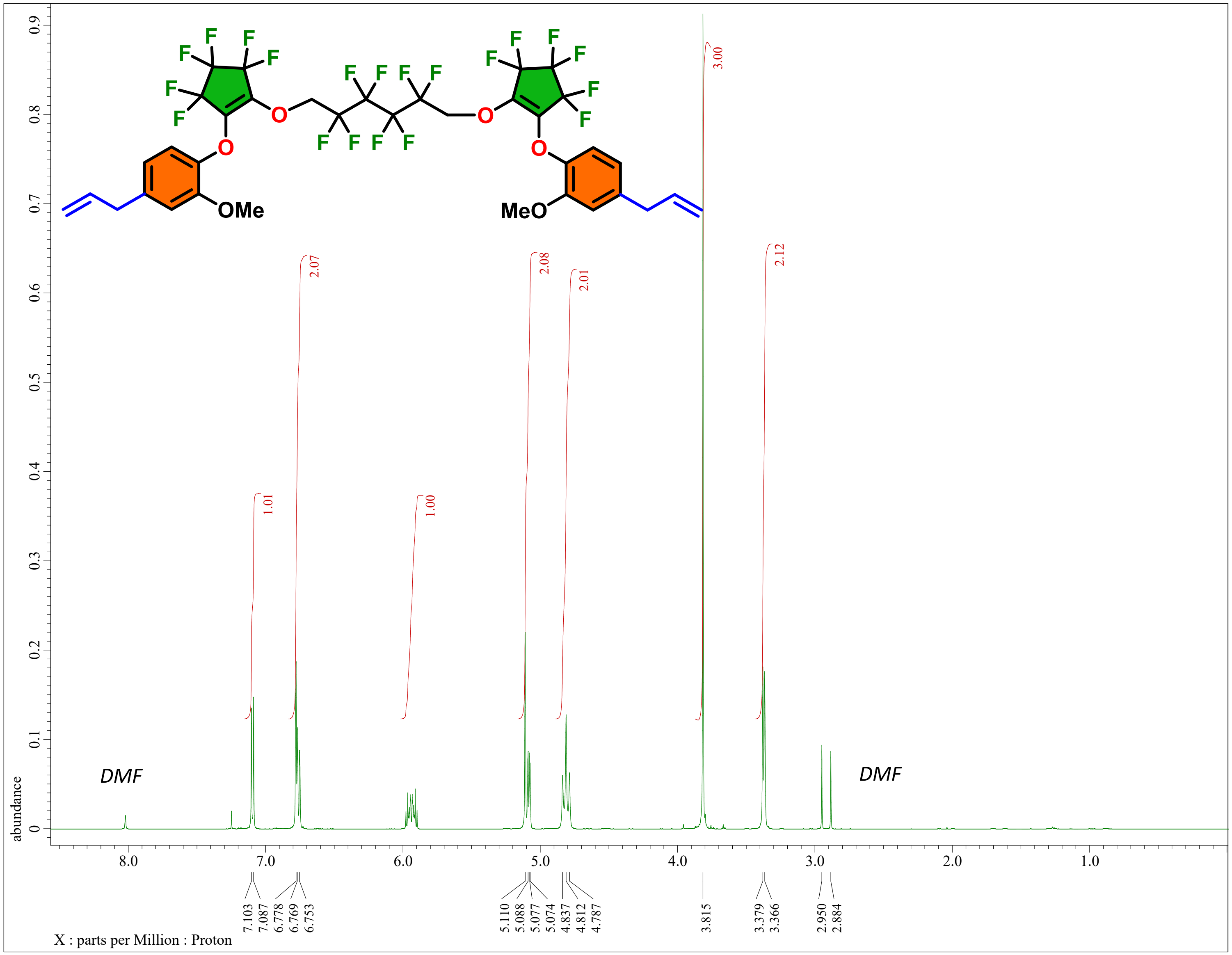

Figure S19. ^{1}H NMR of **M4**.

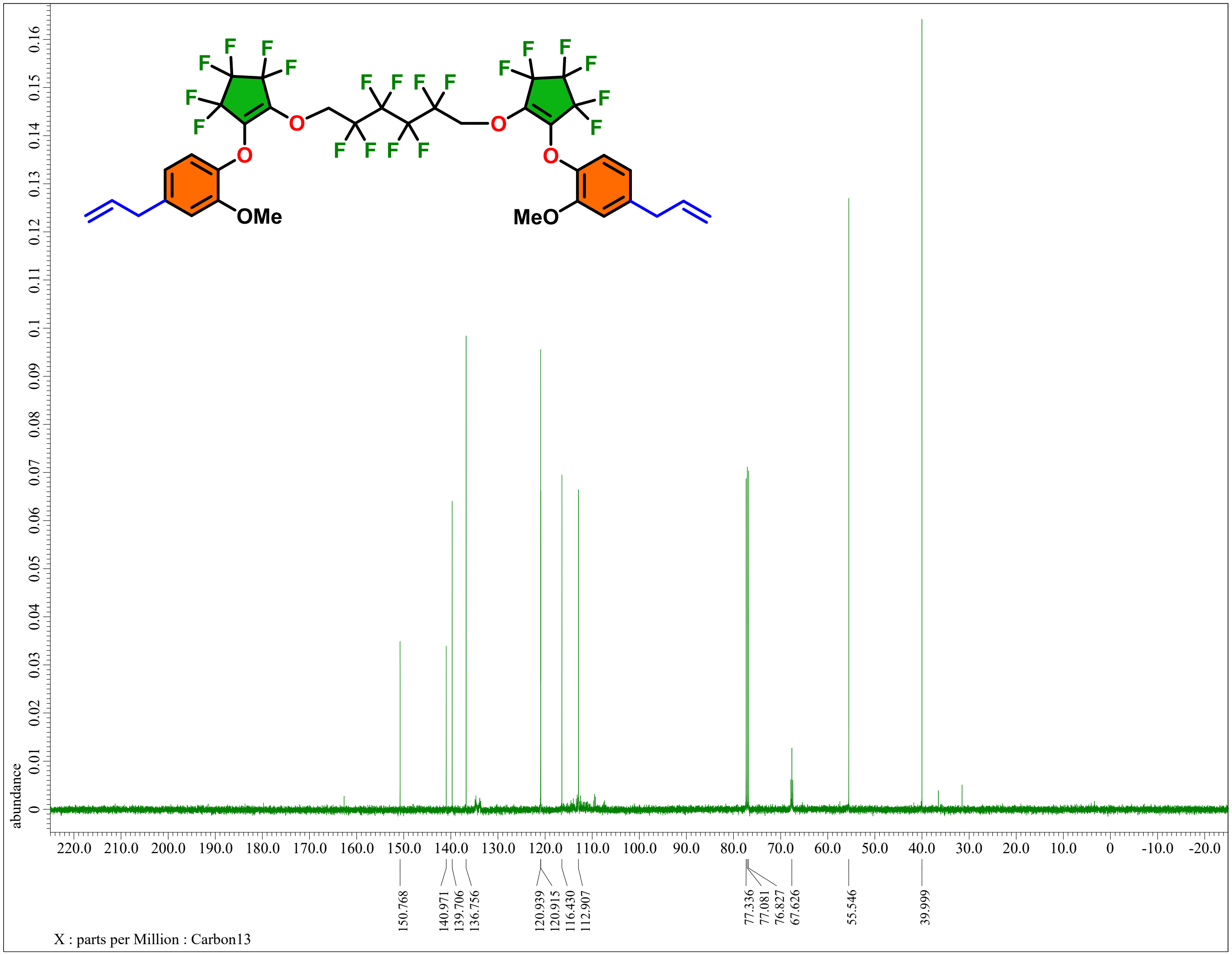

Figure S20. ^{13}C NMR **M4**.

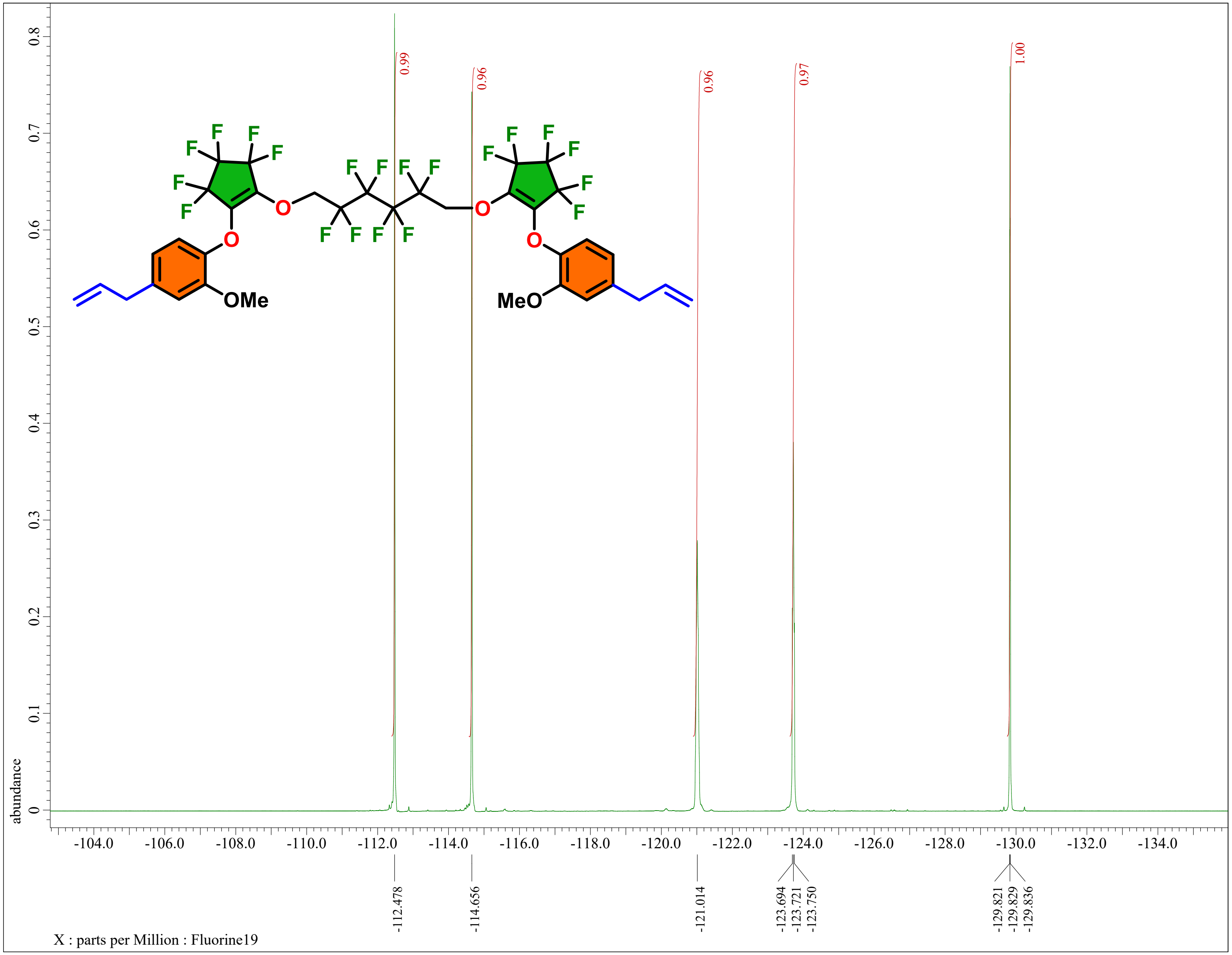

Figure S21. ^{19}F NMR **M4**.

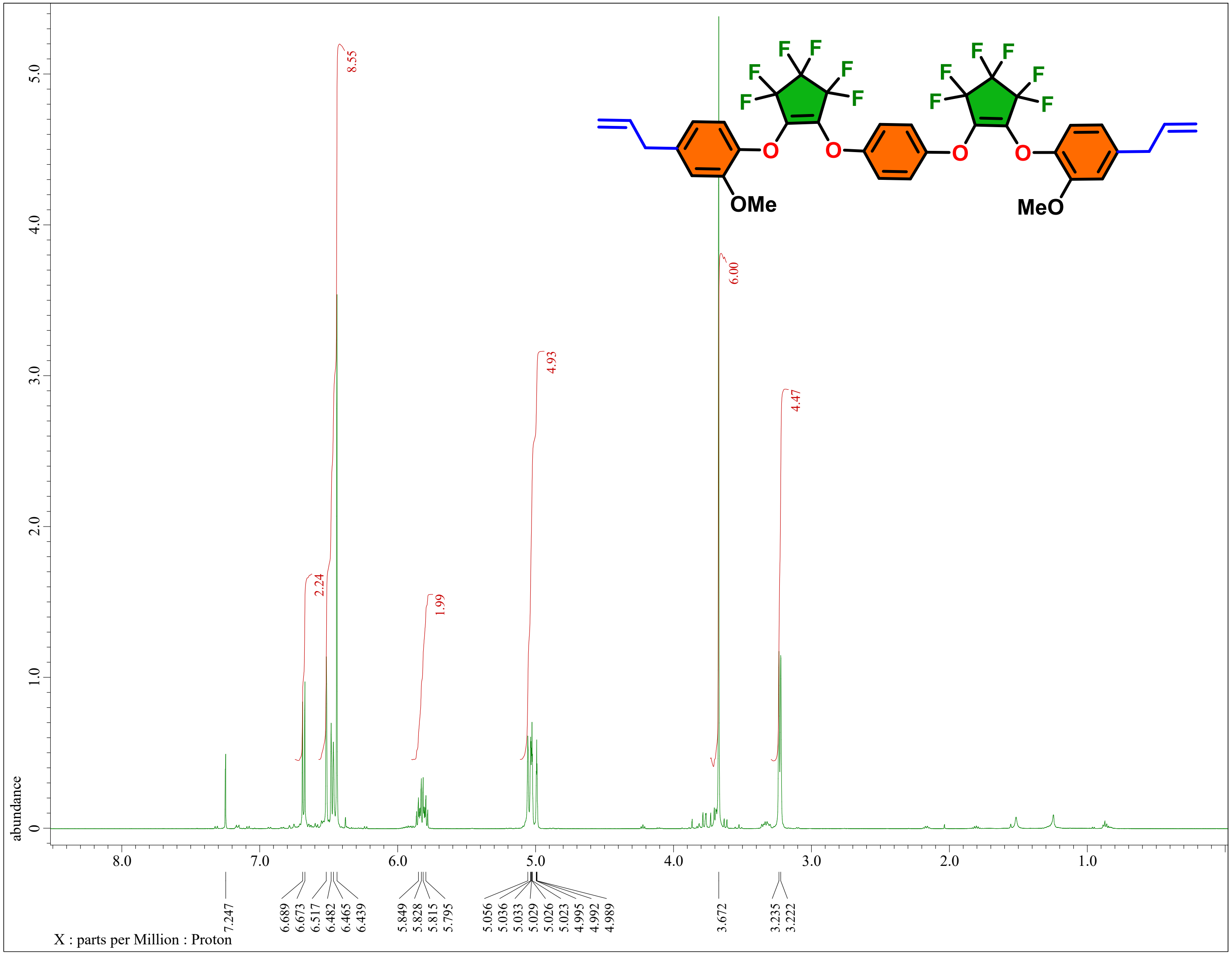

Figure S22. ^{1}H NMR of **M5**.

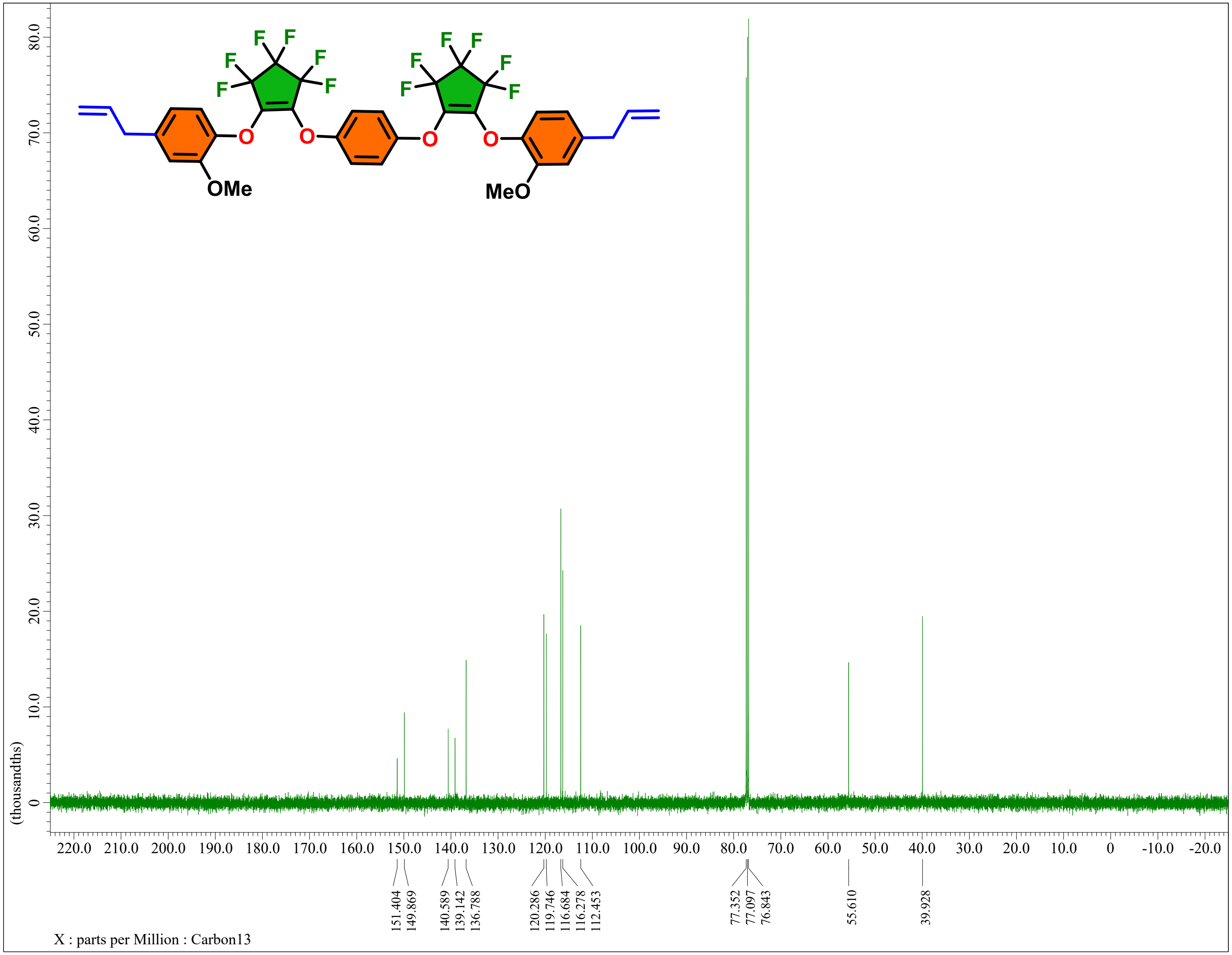

Figure S23. ^{13}C NMR **M5**.

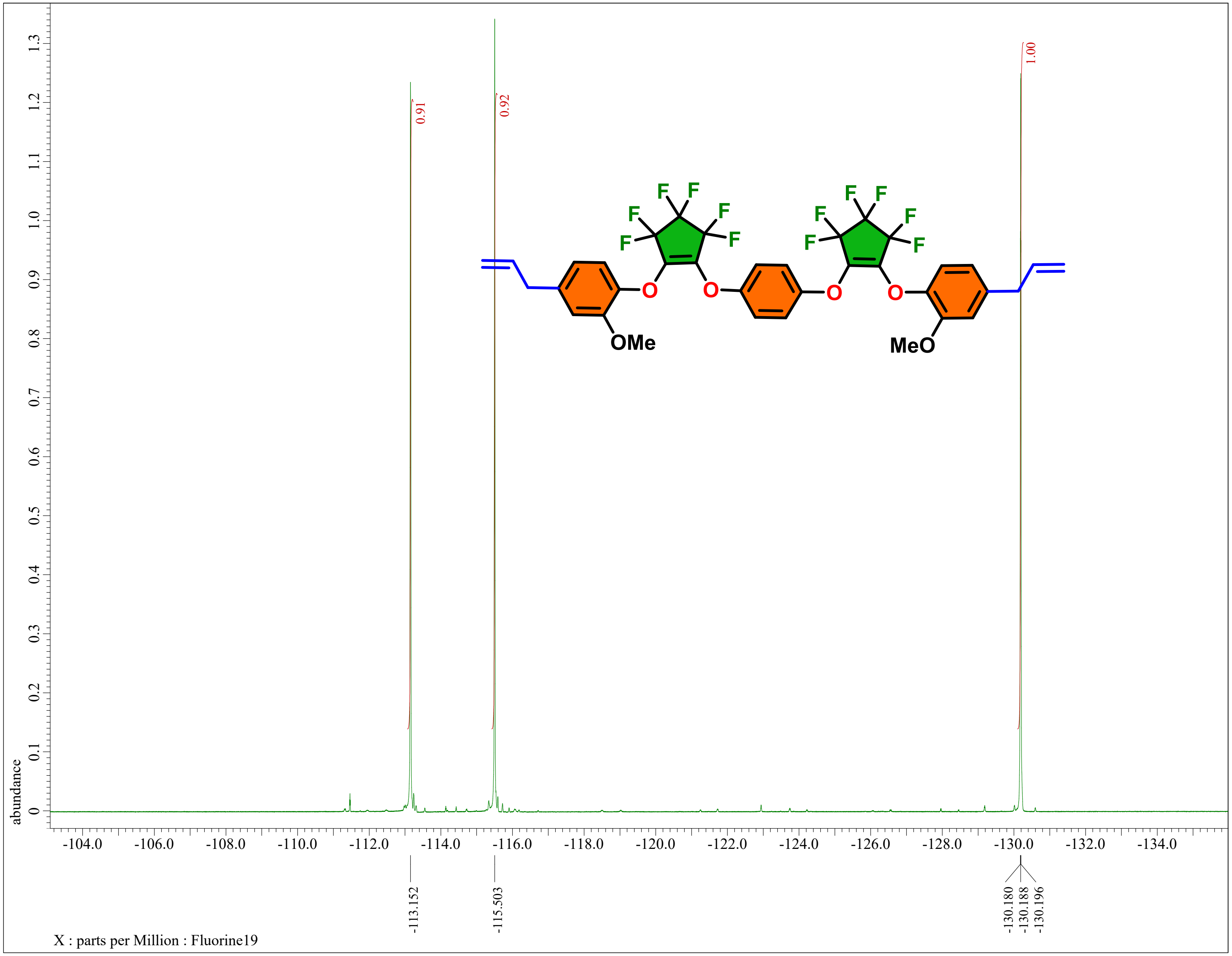

Figure S24. ^{19}F NMR **M5**.

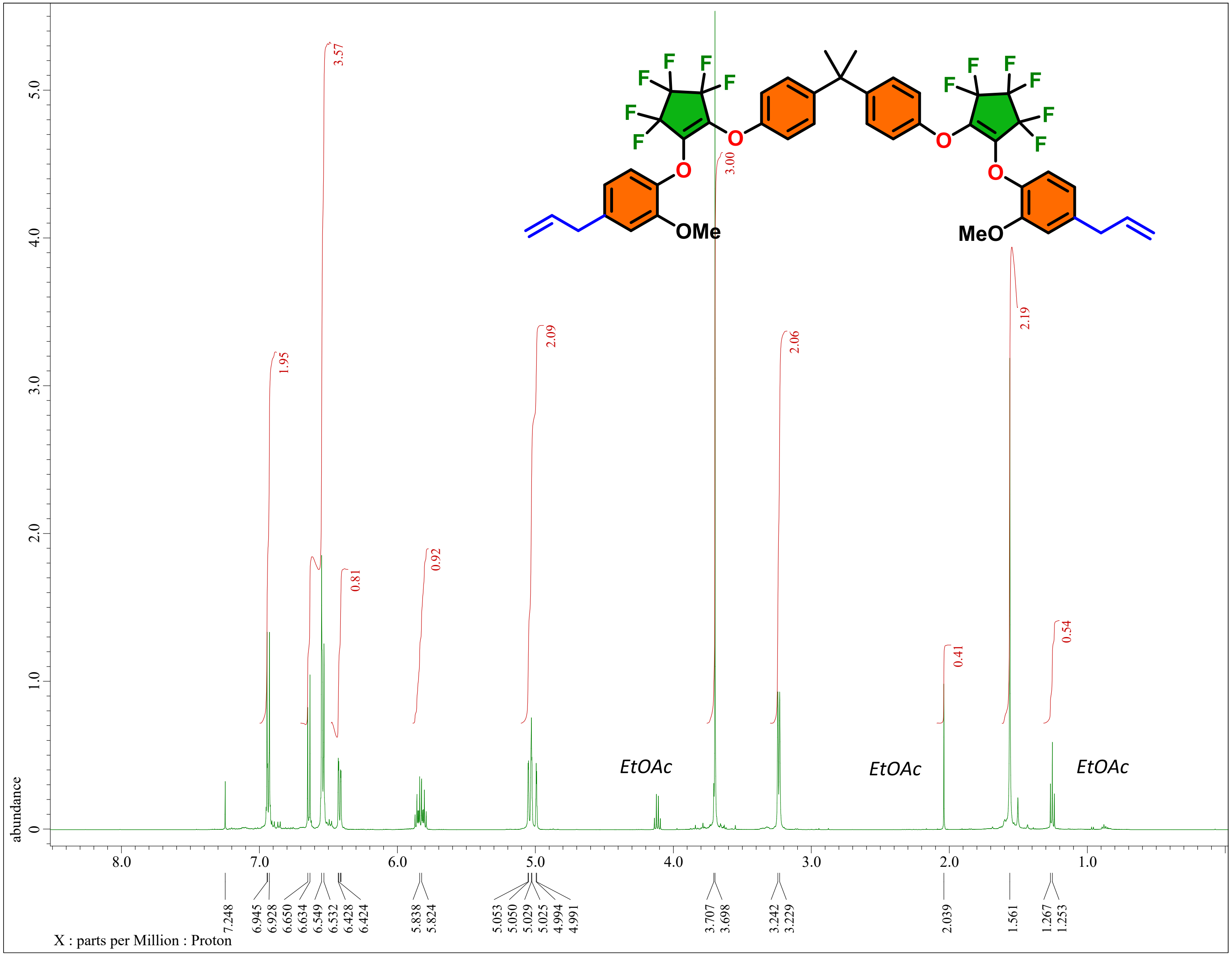

Figure S25. ^{1}H NMR of **M6**.

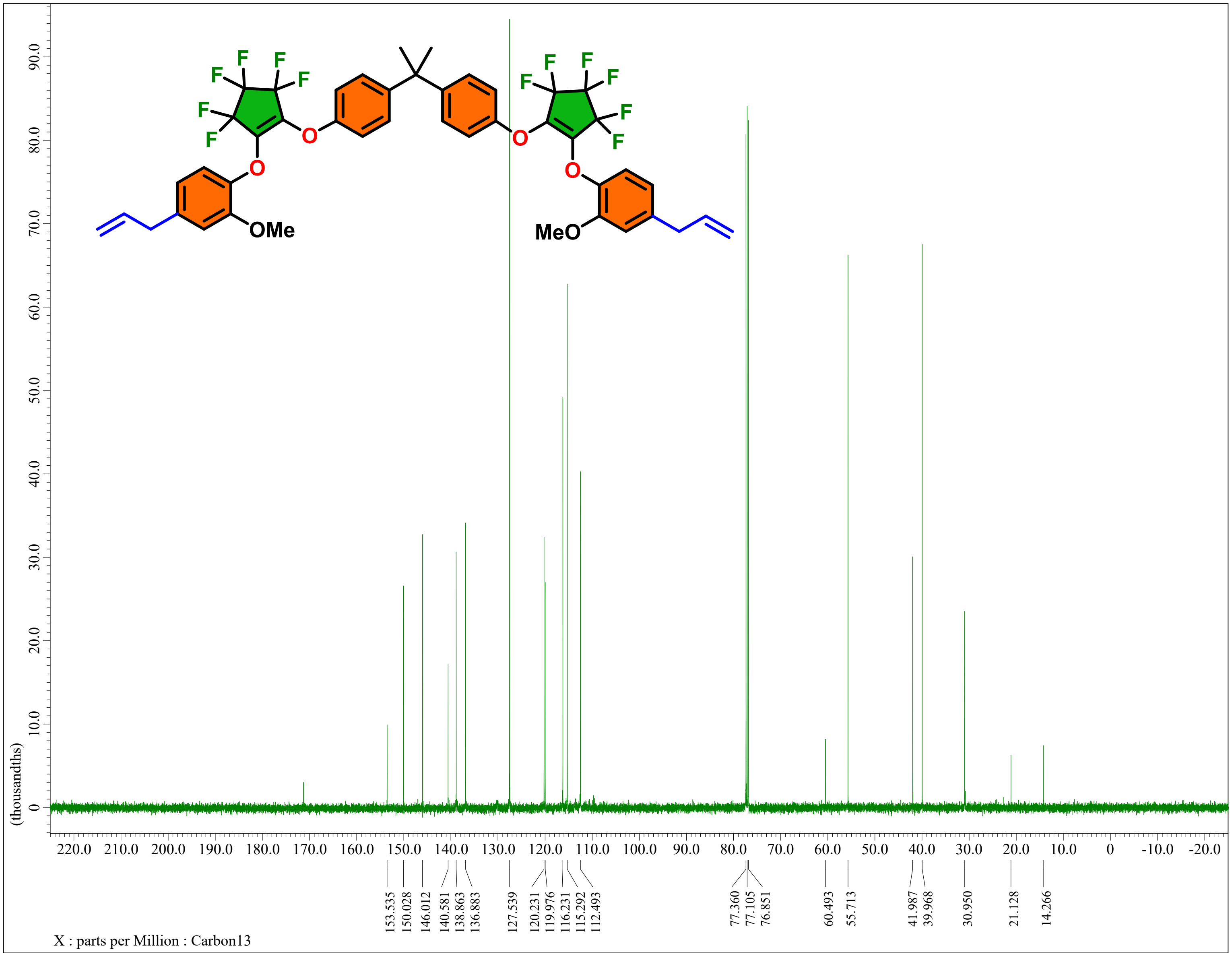

Figure S26. ^{13}C NMR **M6**.

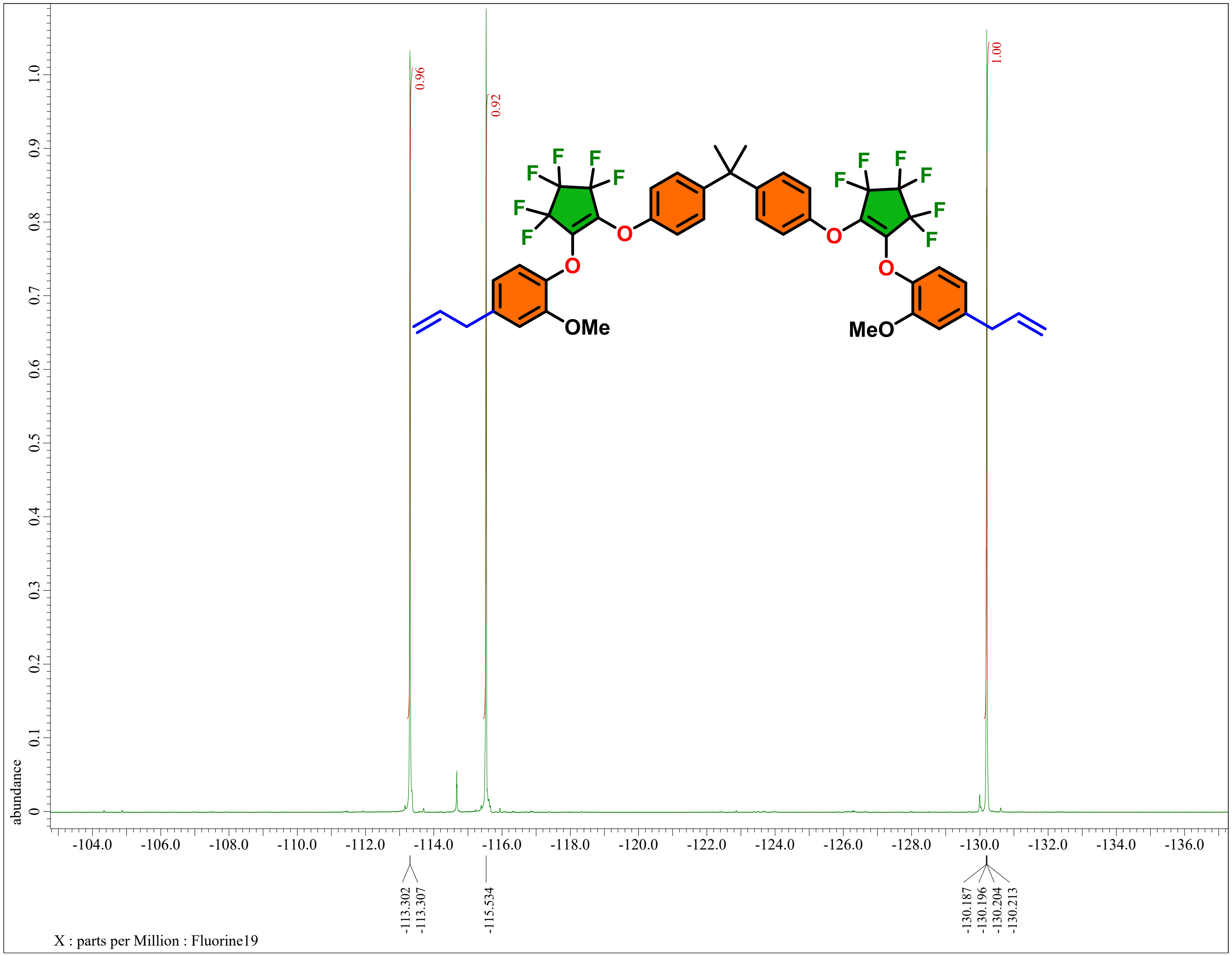

Figure S27. ^{19}F NMR **M6**.

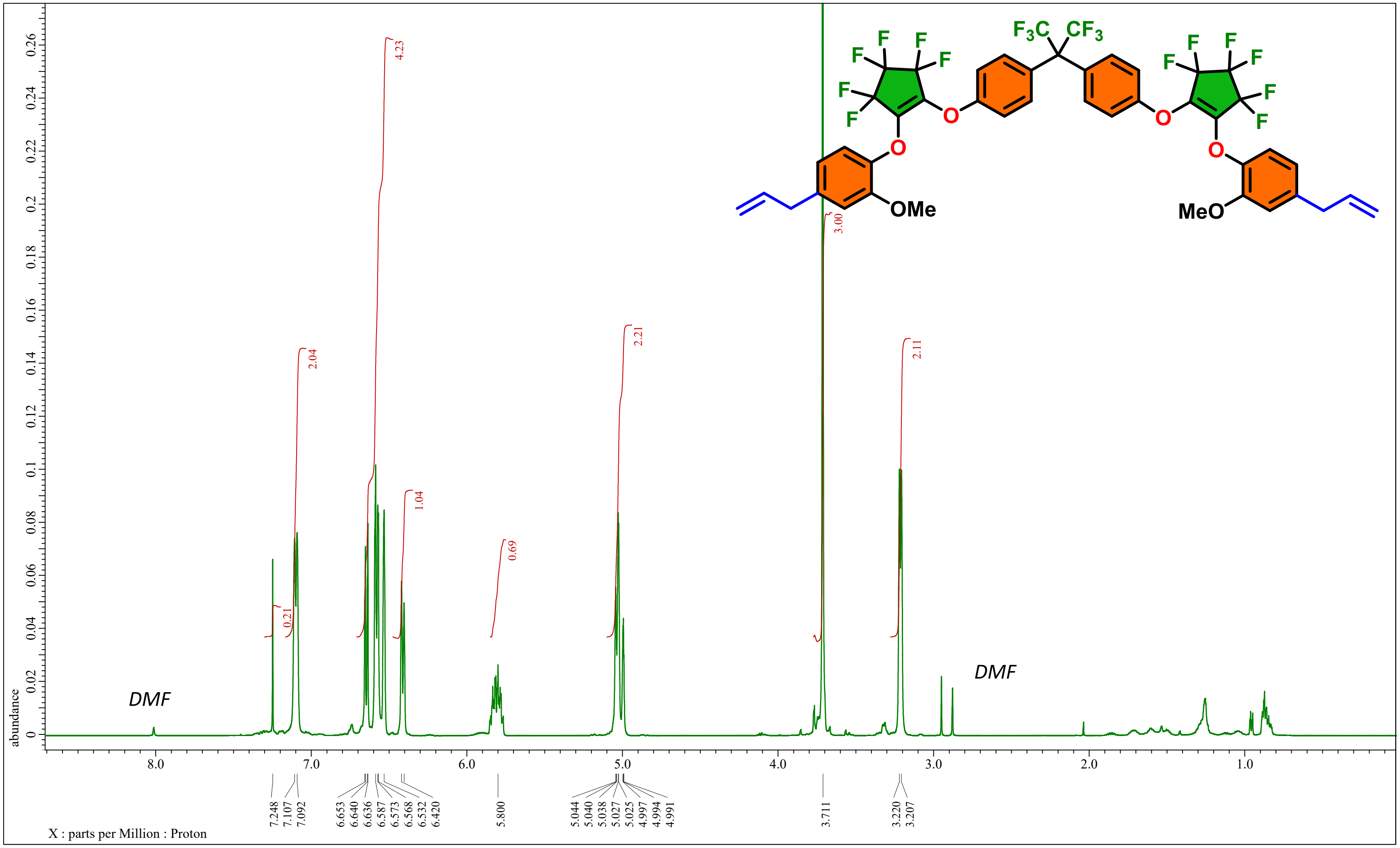

Figure S28. ^{1}H NMR of **M7**.

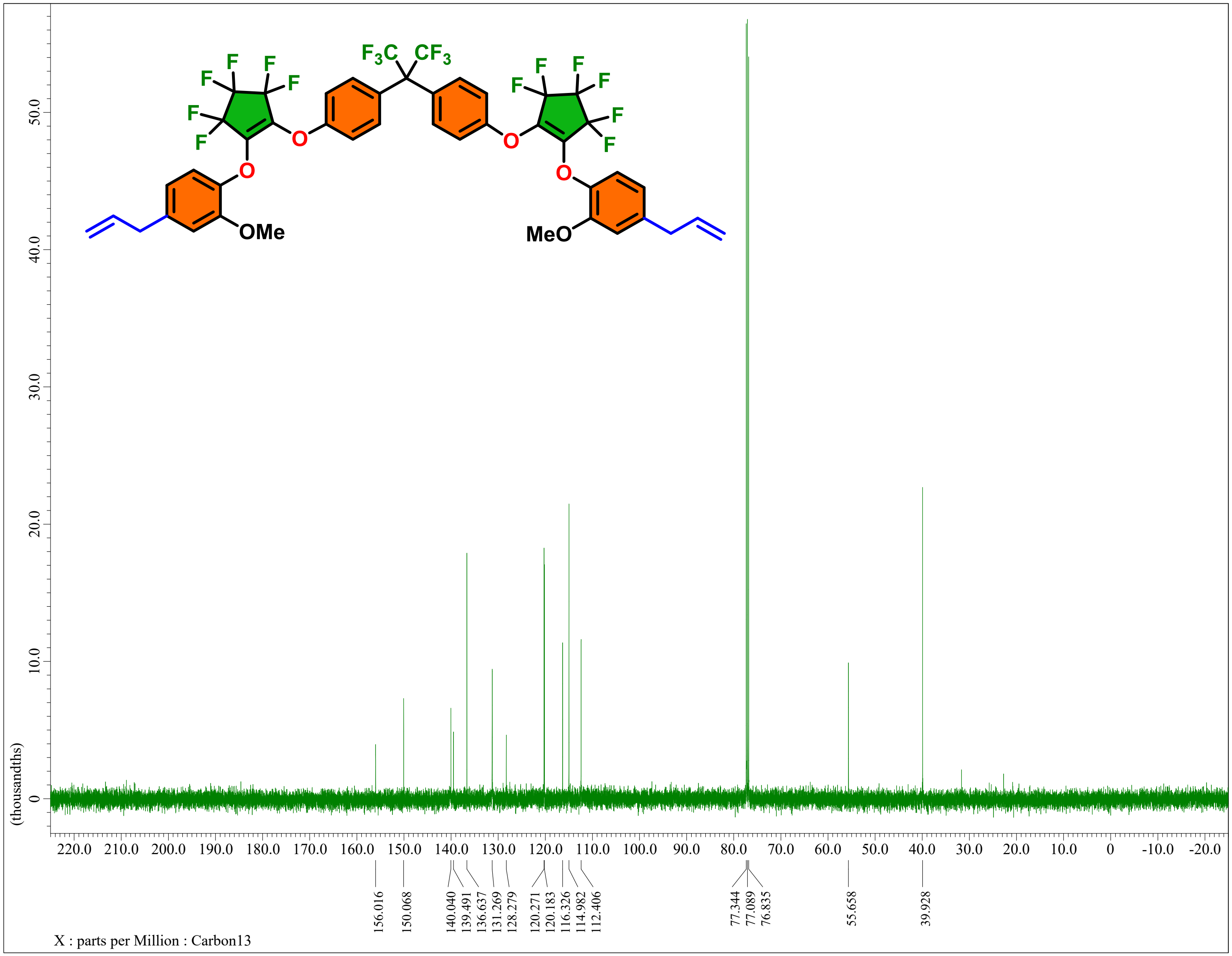

Figure S29. ^{13}C NMR **M7**.

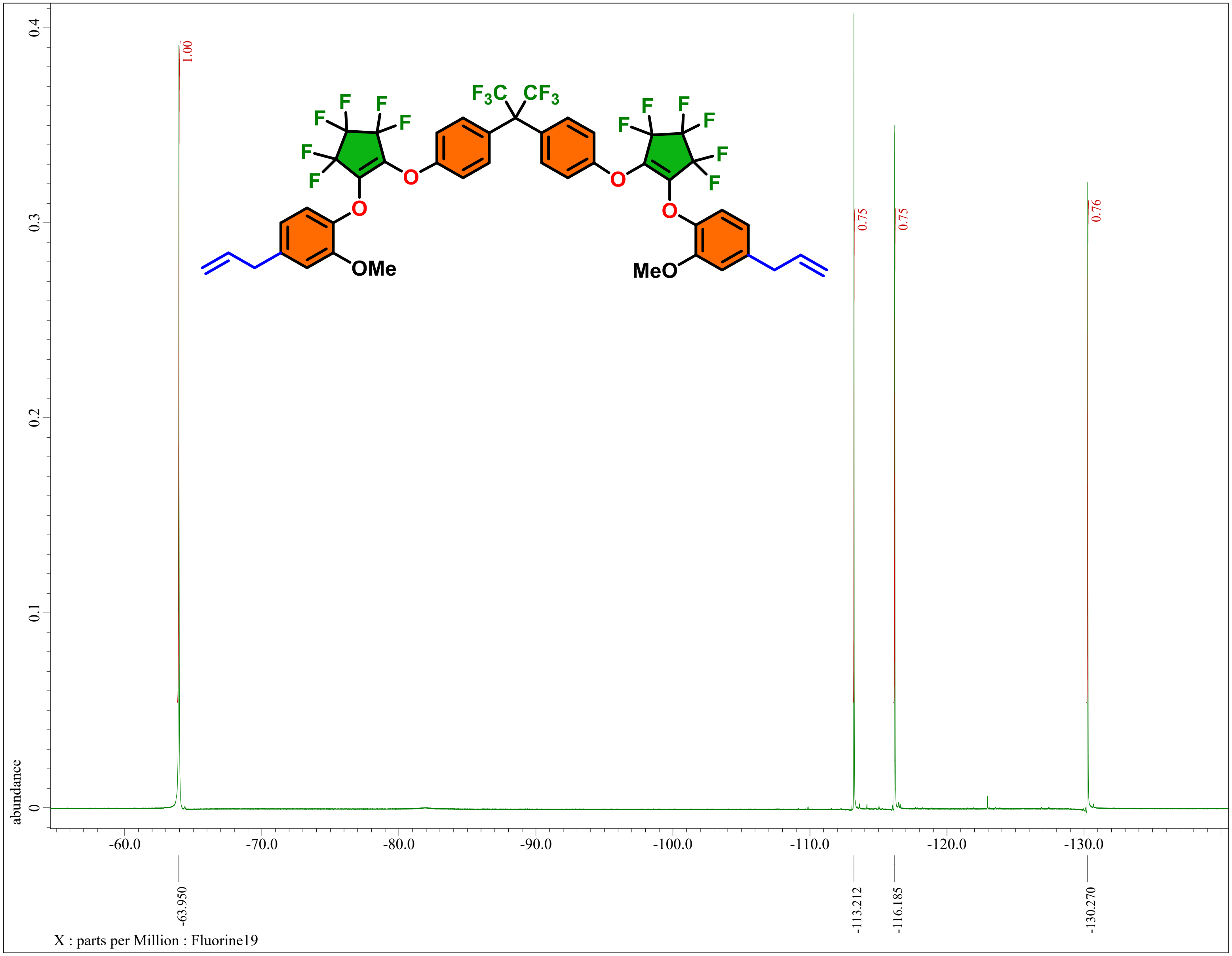

Figure S30. ^{19}F NMR **M7**.

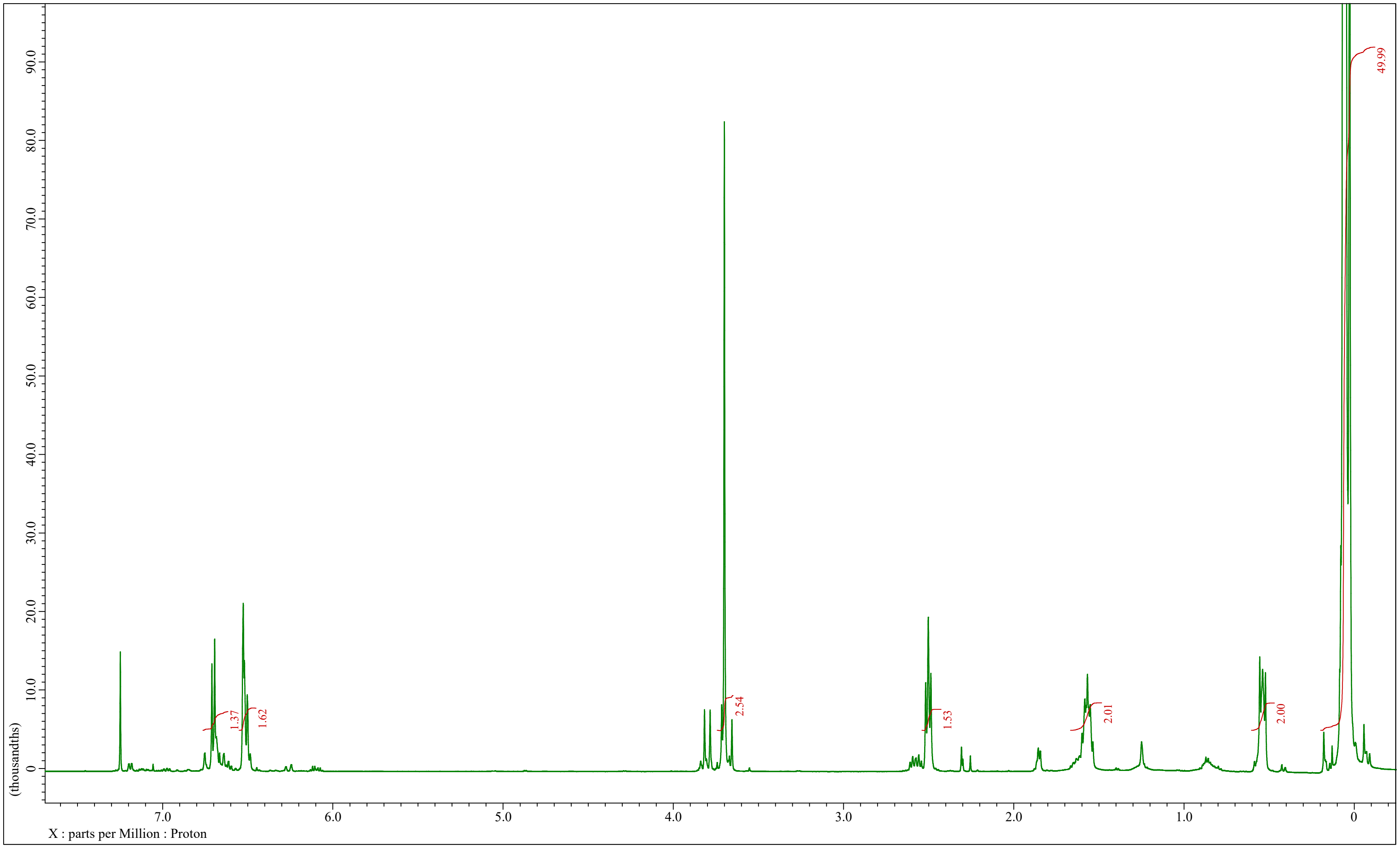

Figure S31. ^{1}H NMR of **P1**.

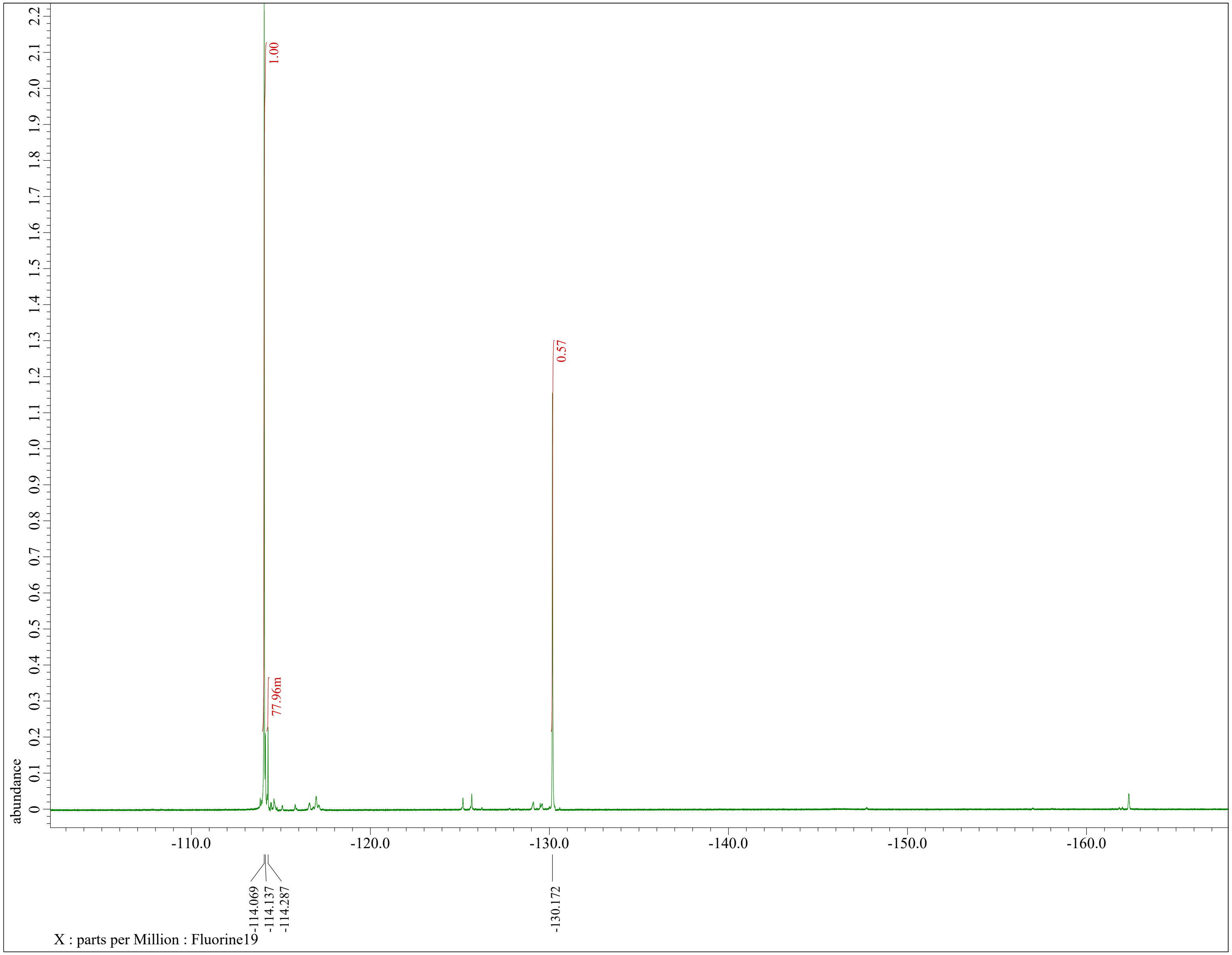

Figure S32. ^{19}F NMR of **P1**.

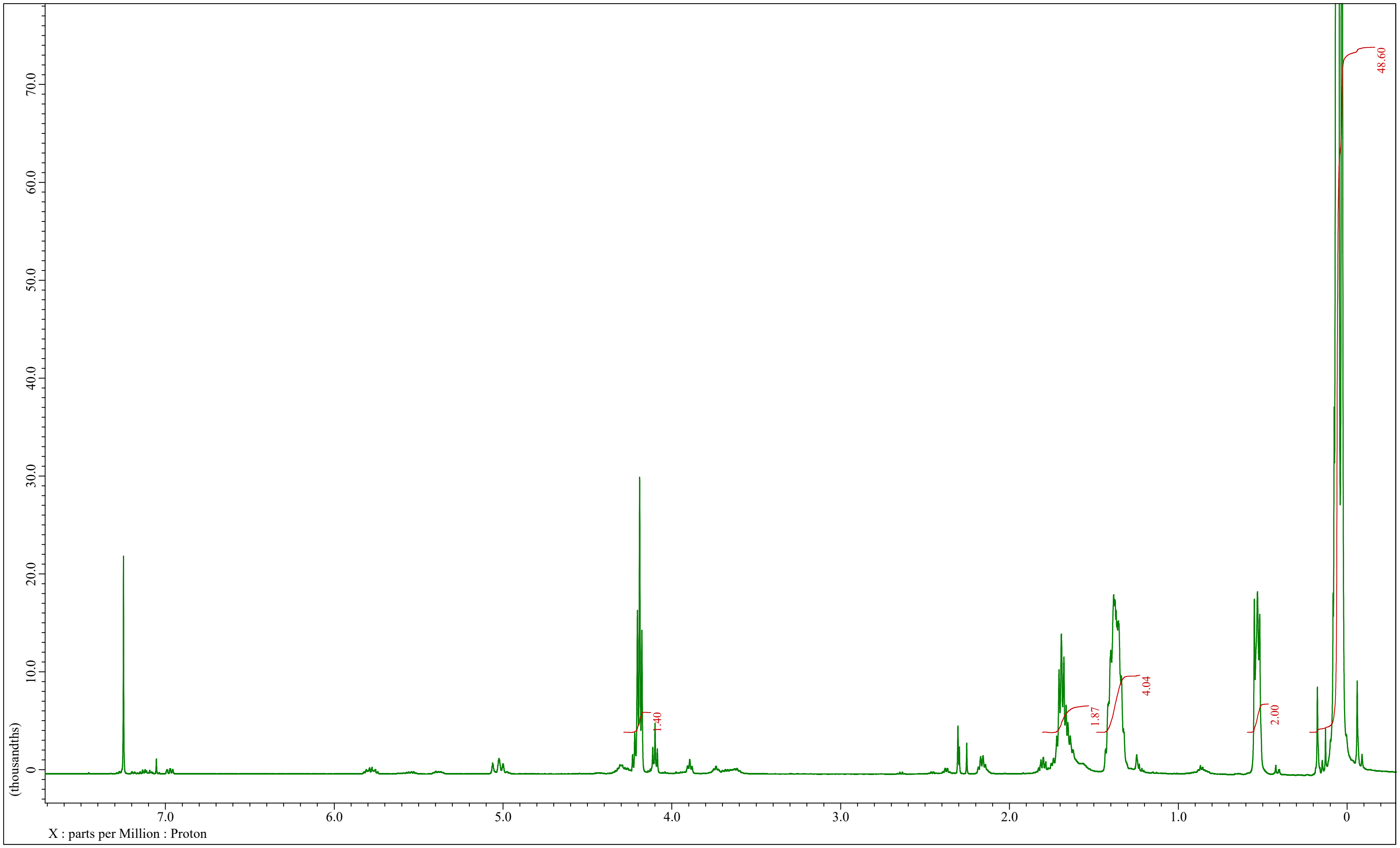

Figure S33. ^{1}H NMR of **P2**.

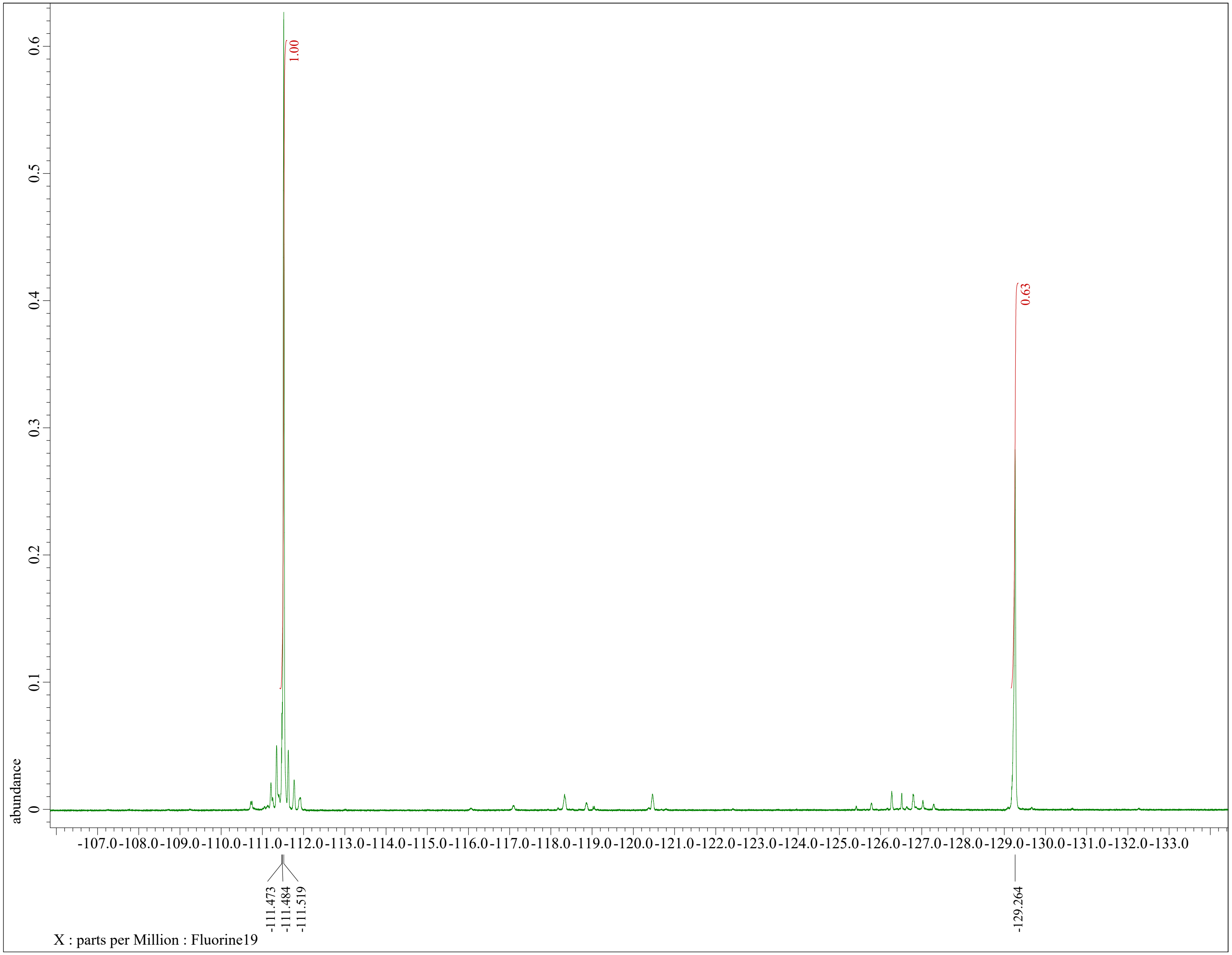

Figure S34. ^{19}F NMR of **P2**.

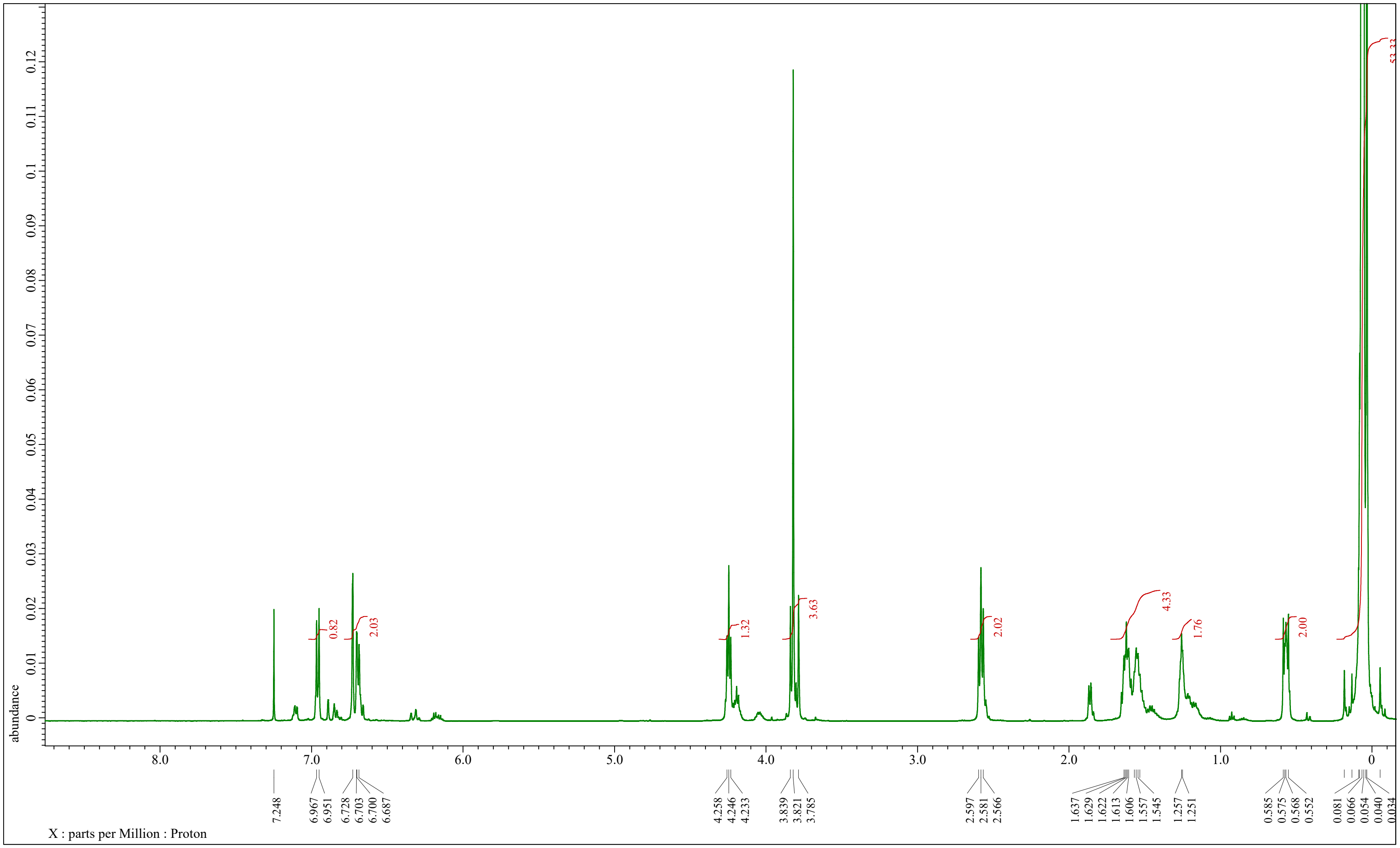

Figure S35. ^{1}H NMR of **P3**.

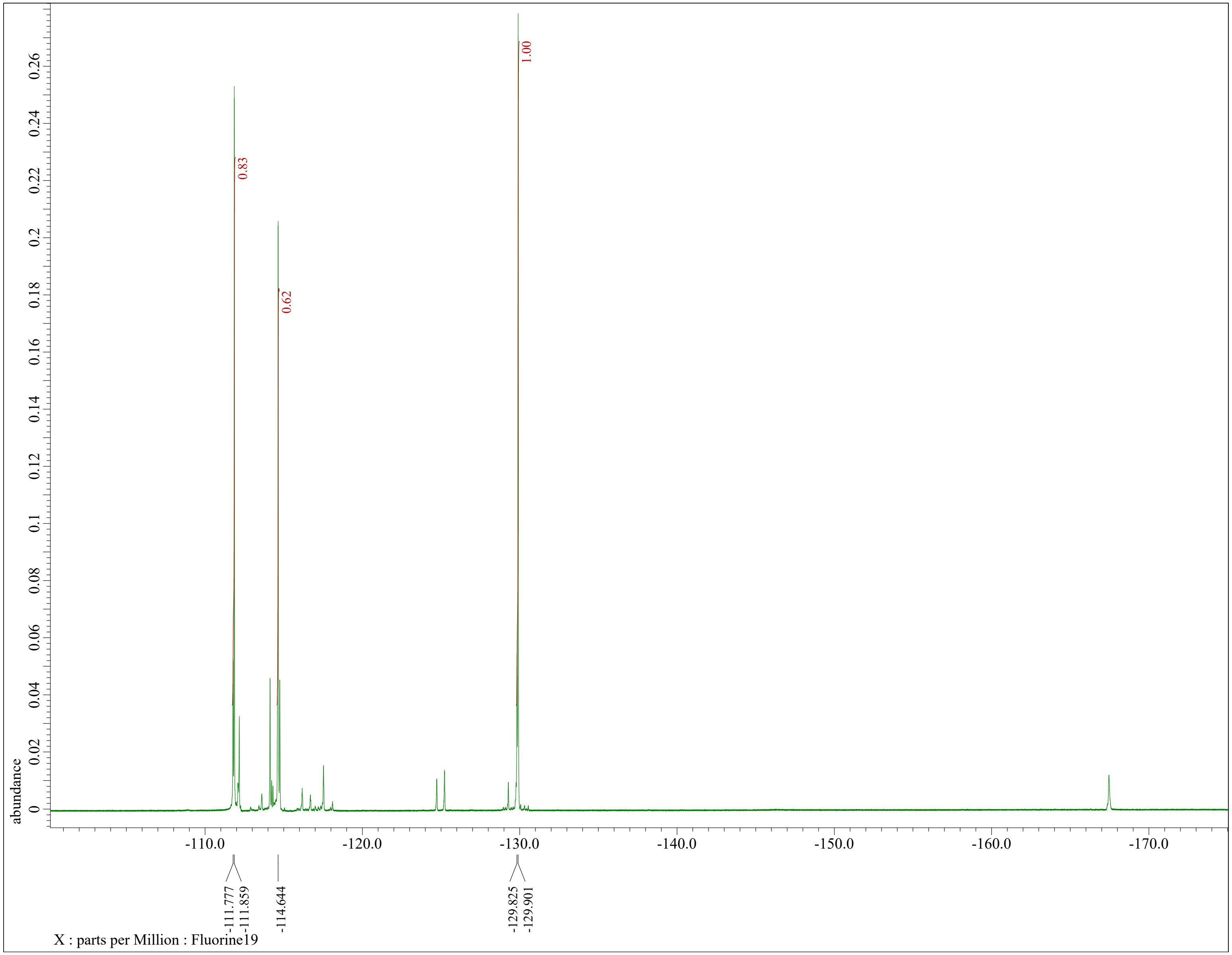

Figure S36. ^{19}F NMR of **P3**.

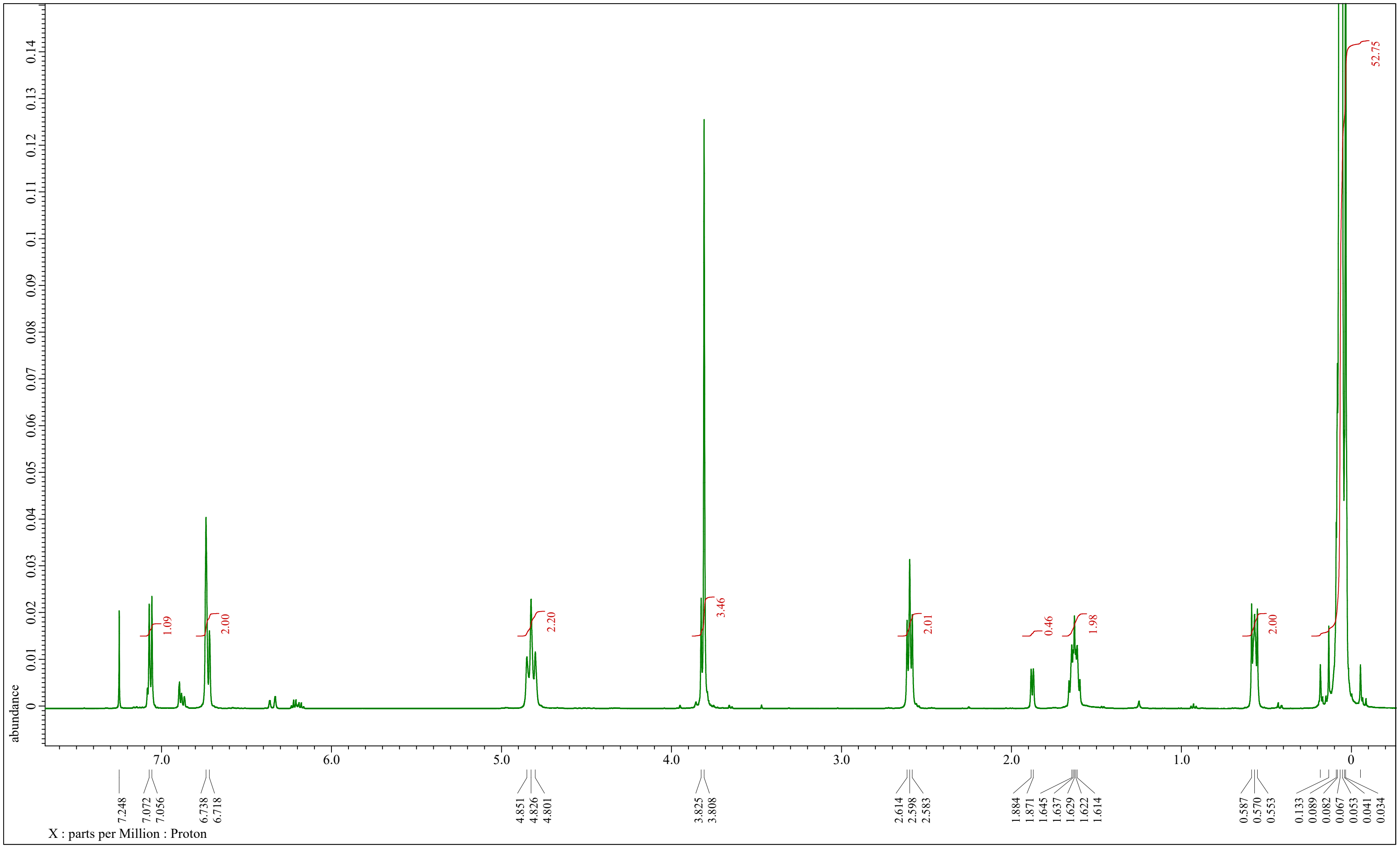

Figure S37. ^{1}H NMR of **P4**.

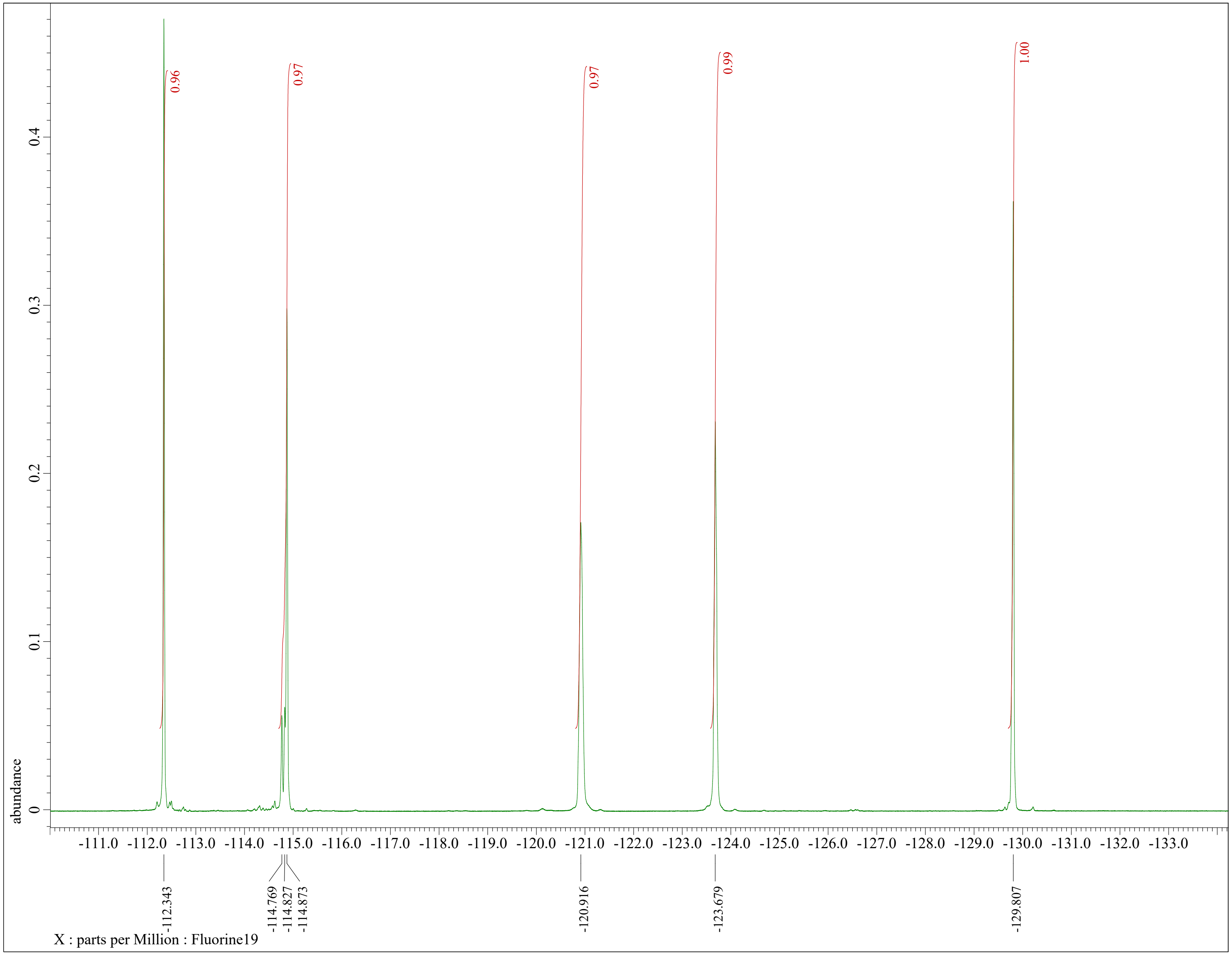

Figure S38. ^{19}F NMR of **P4**.

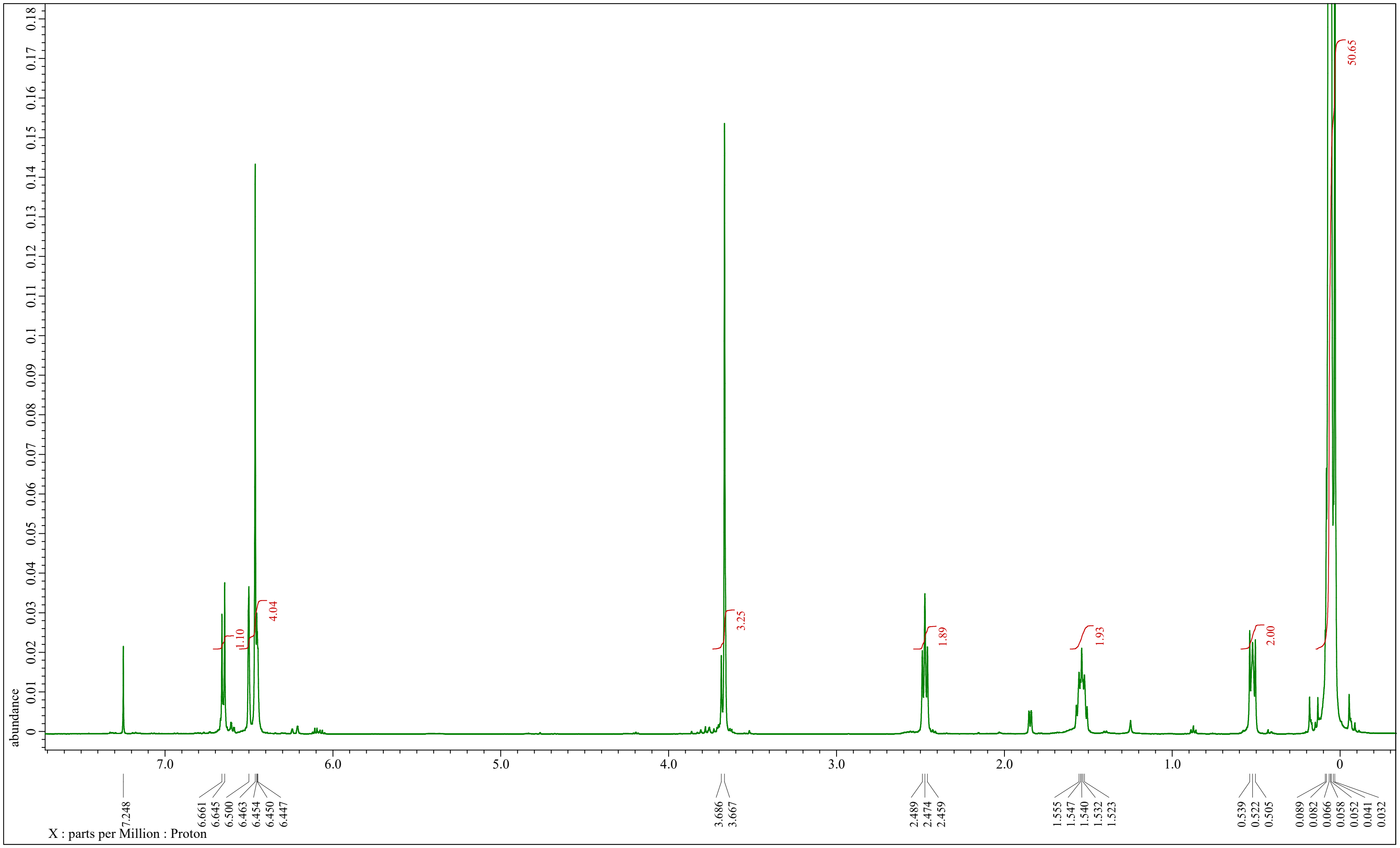

Figure S39. ^{1}H NMR of **P5**.

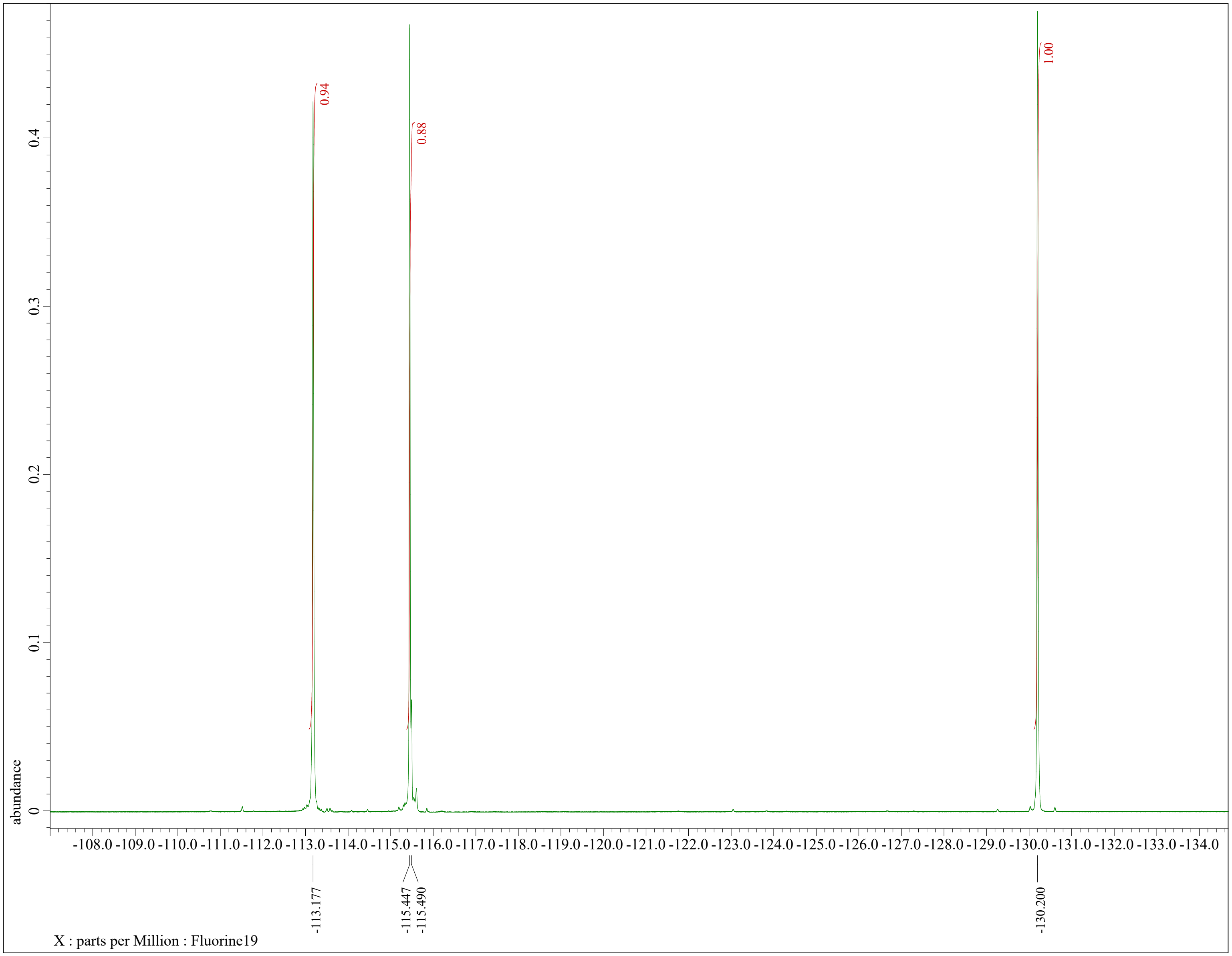

Figure S40. ^{19}F NMR of **P5**.

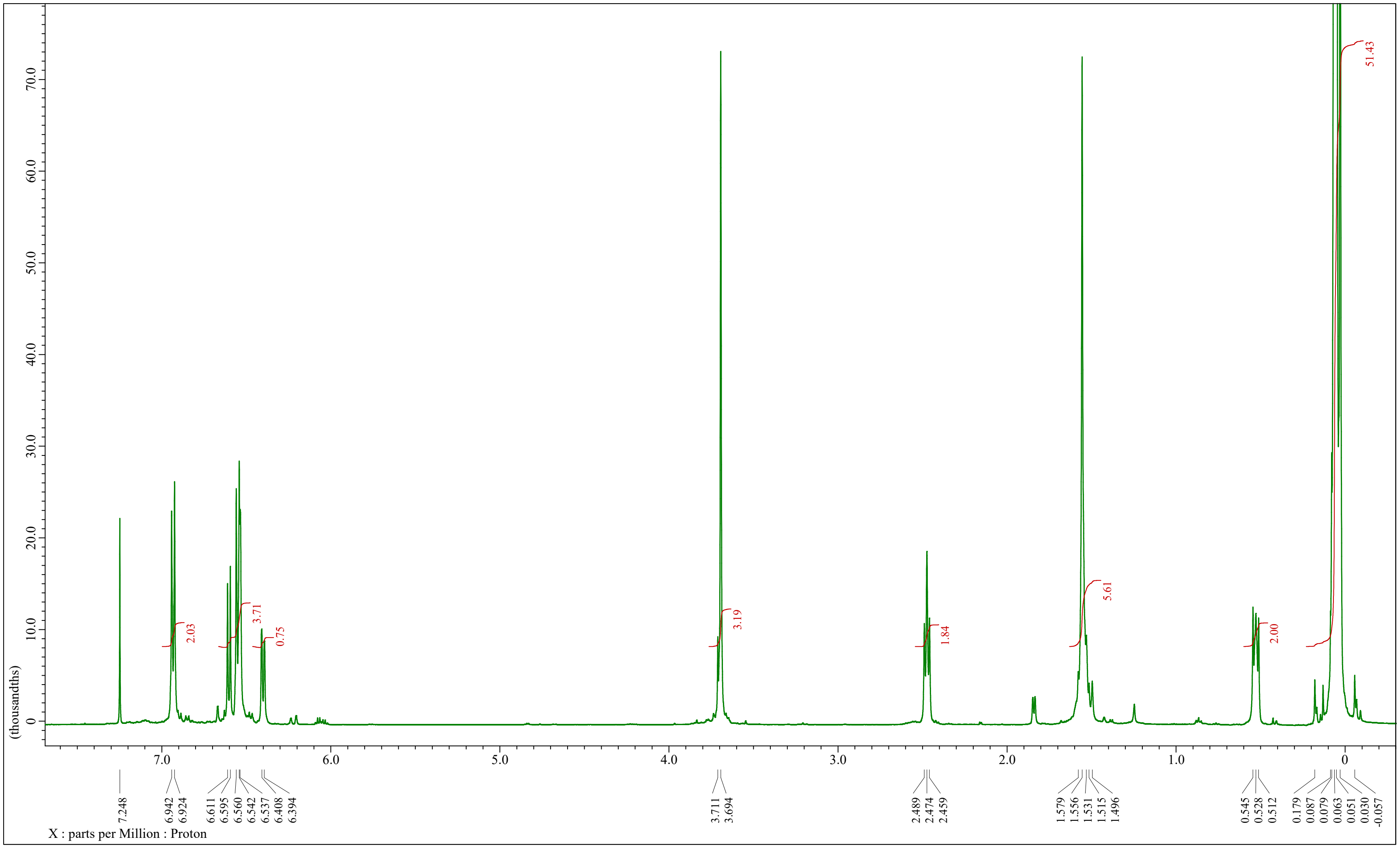

Figure S41. ^{1}H NMR of **P6**.

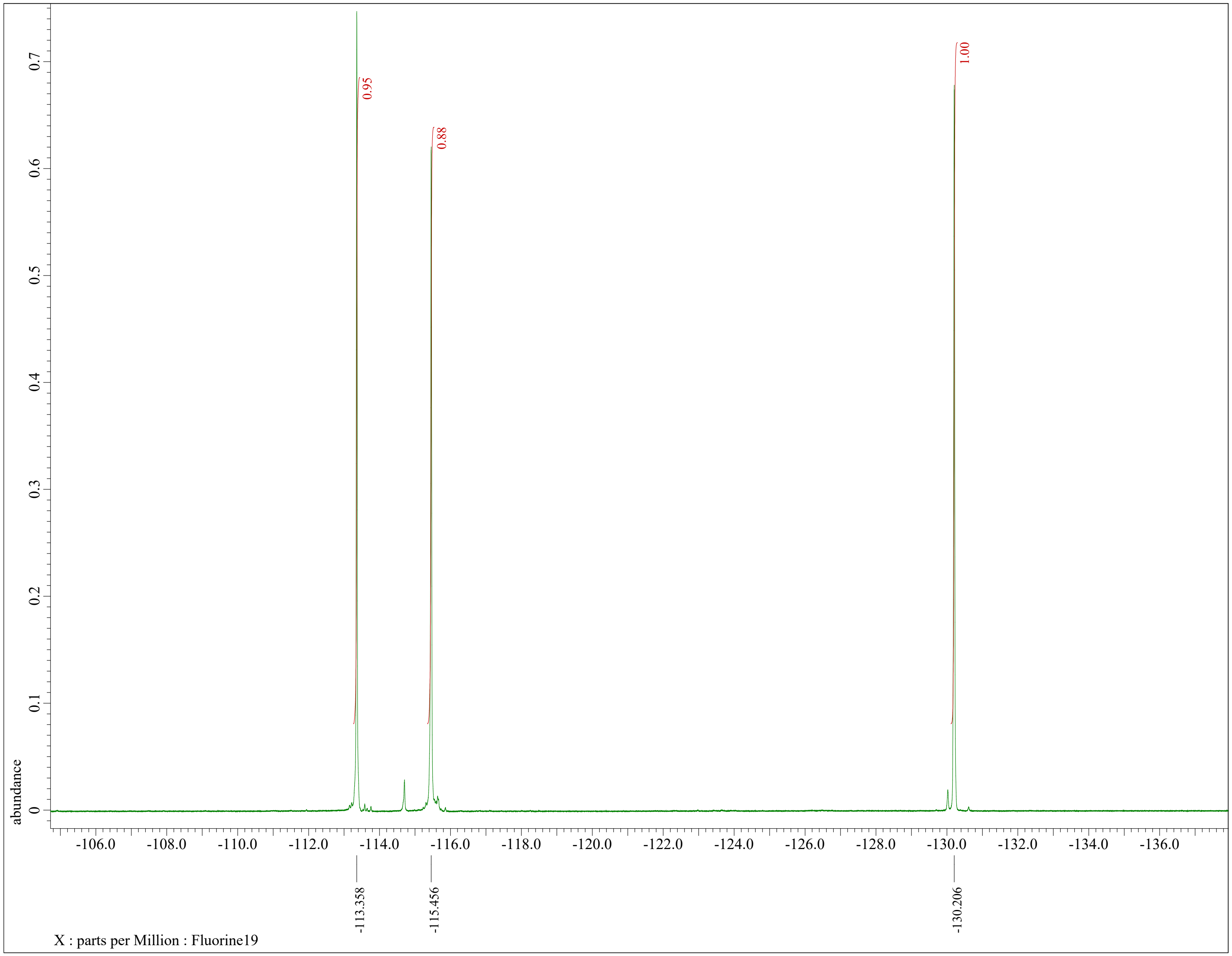

Figure S42. ^{19}F NMR of **P6**.

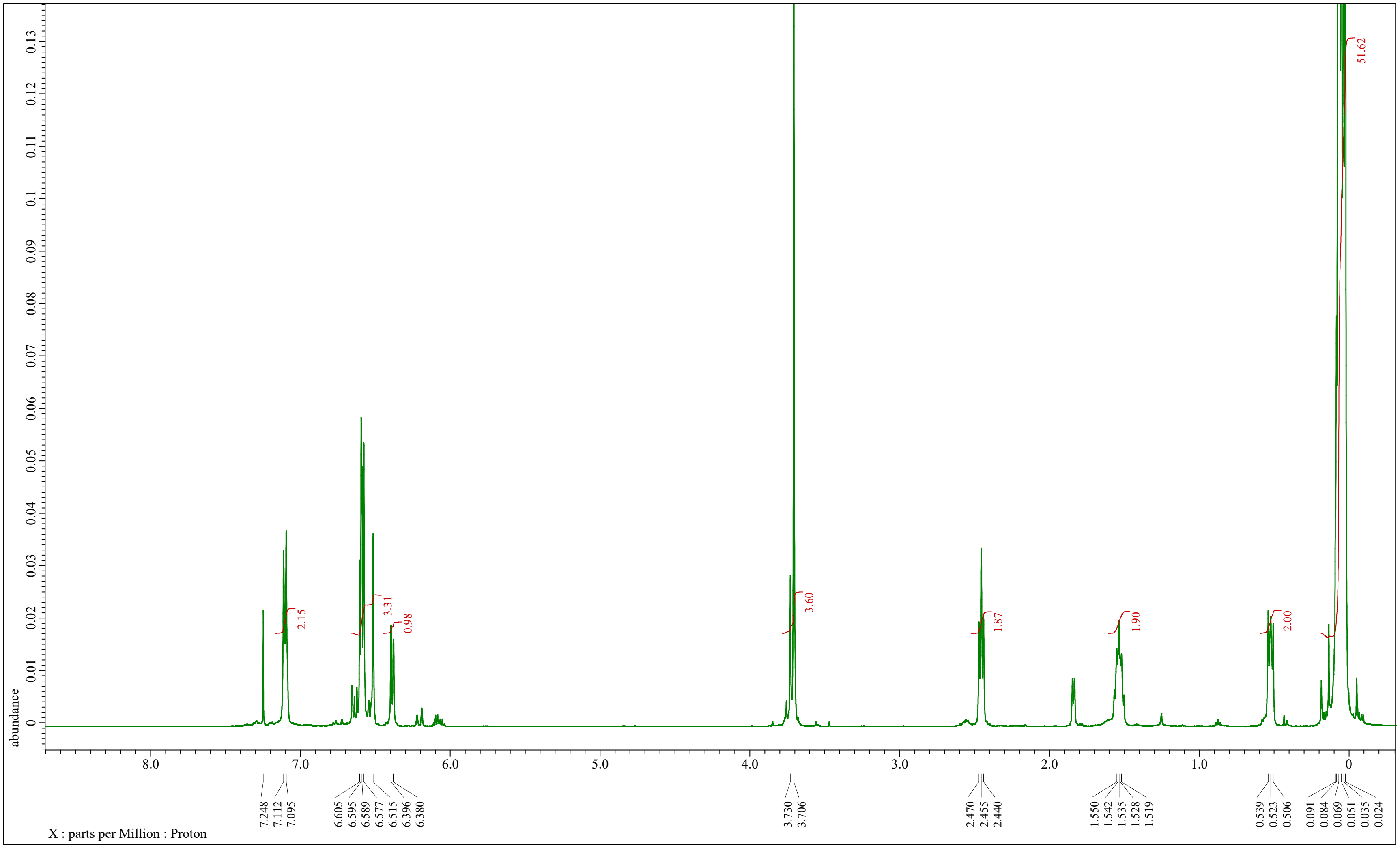

Figure S43. ^{1}H NMR of **P7**.

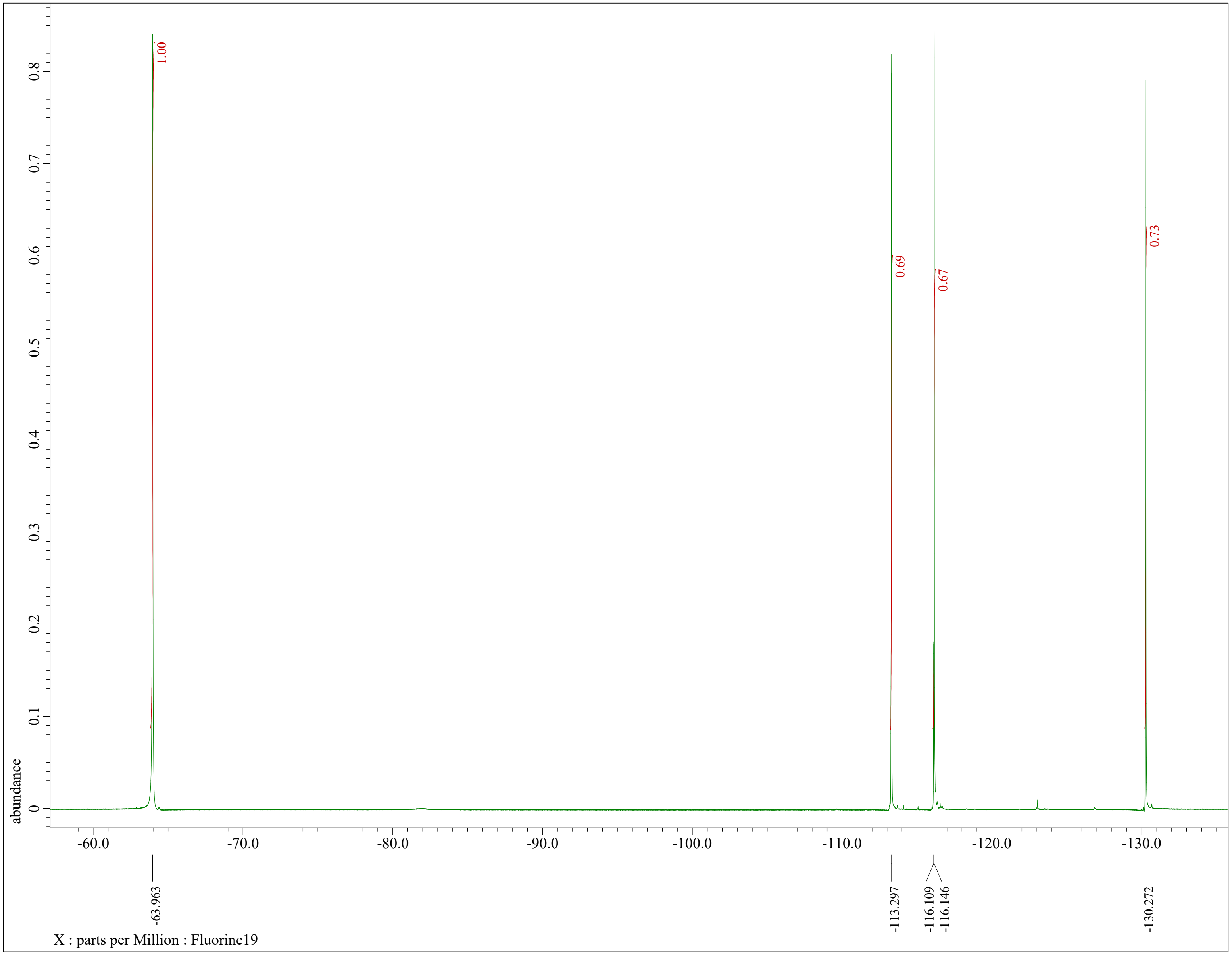

Figure S44. ^{19}F NMR of **P7**.

Table S1. ^{1}H NMR End-group Calculations

entry	Area(RU)	#H(RU)	#H(EG)	AREA(EG)	DP	MW(RU)	EG MW	MW
**P1**	20.9	2	3	3	10.45	15,717	503	16,220
**P2**	12.9	2	3	3	6.45	8,695	347	9,042
**P3**	13.6	2	3	3	6.80	12,172	791	12,963
**P4**	12.6	2	3	3	6.30	12,191	935	13,126
**P5**	18.2	2	3	3	9.10	16,225	783	17,008
**P6**	15.6	2	3	3	7.80	14,828	901	15,729
**P7**	14.6	2	3	3	7.30	14,666	1,009	15,675

Key: RU = repeat unit ((−O-Si$(CH_3)_2C\underline{H_2}$−); EG = end-group (−CH=CH_2); DP = degree of polymerization; MW = molecular weight

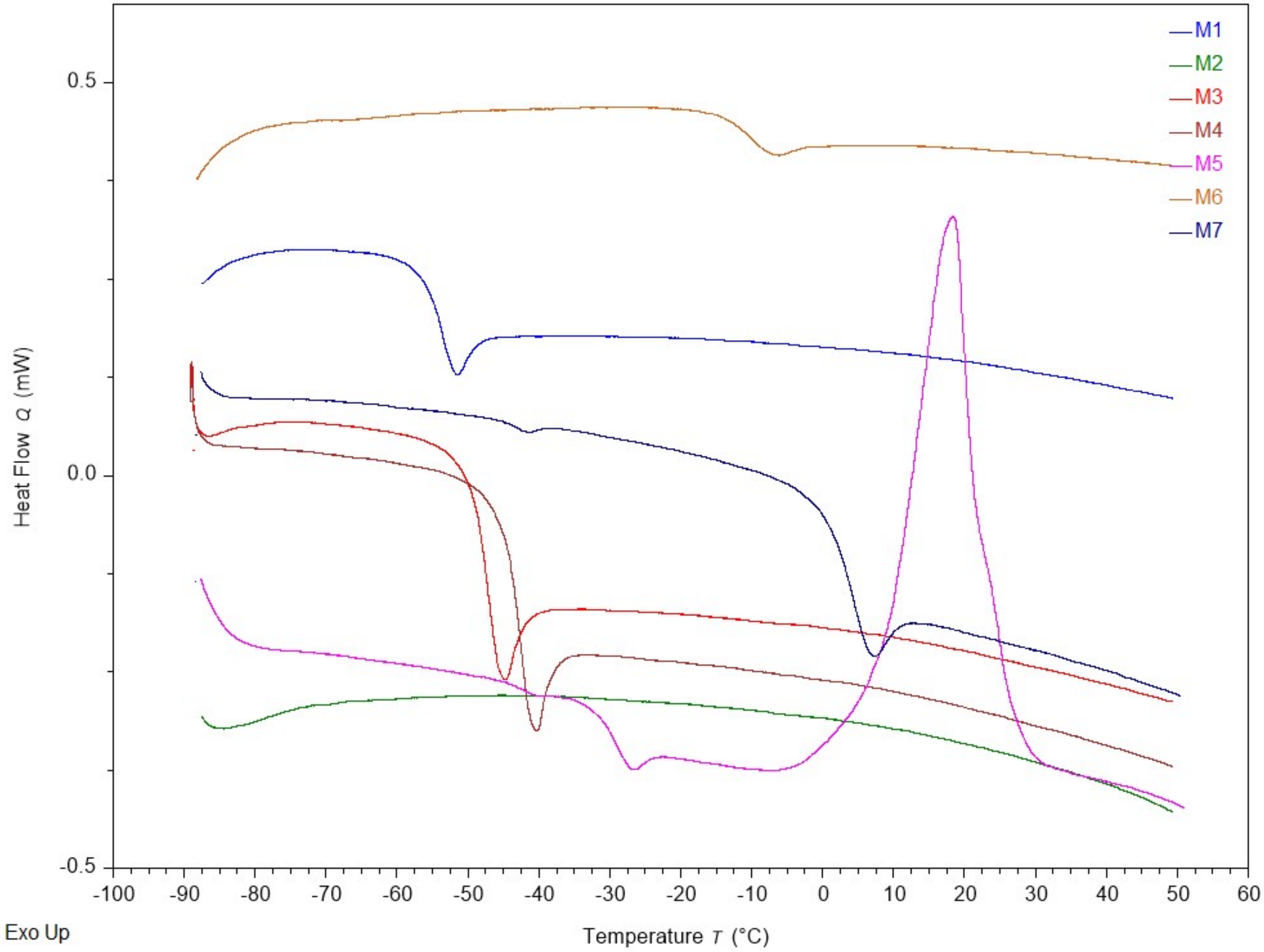

Figure S47. DSC thermograms of monomers **M1−M7**.

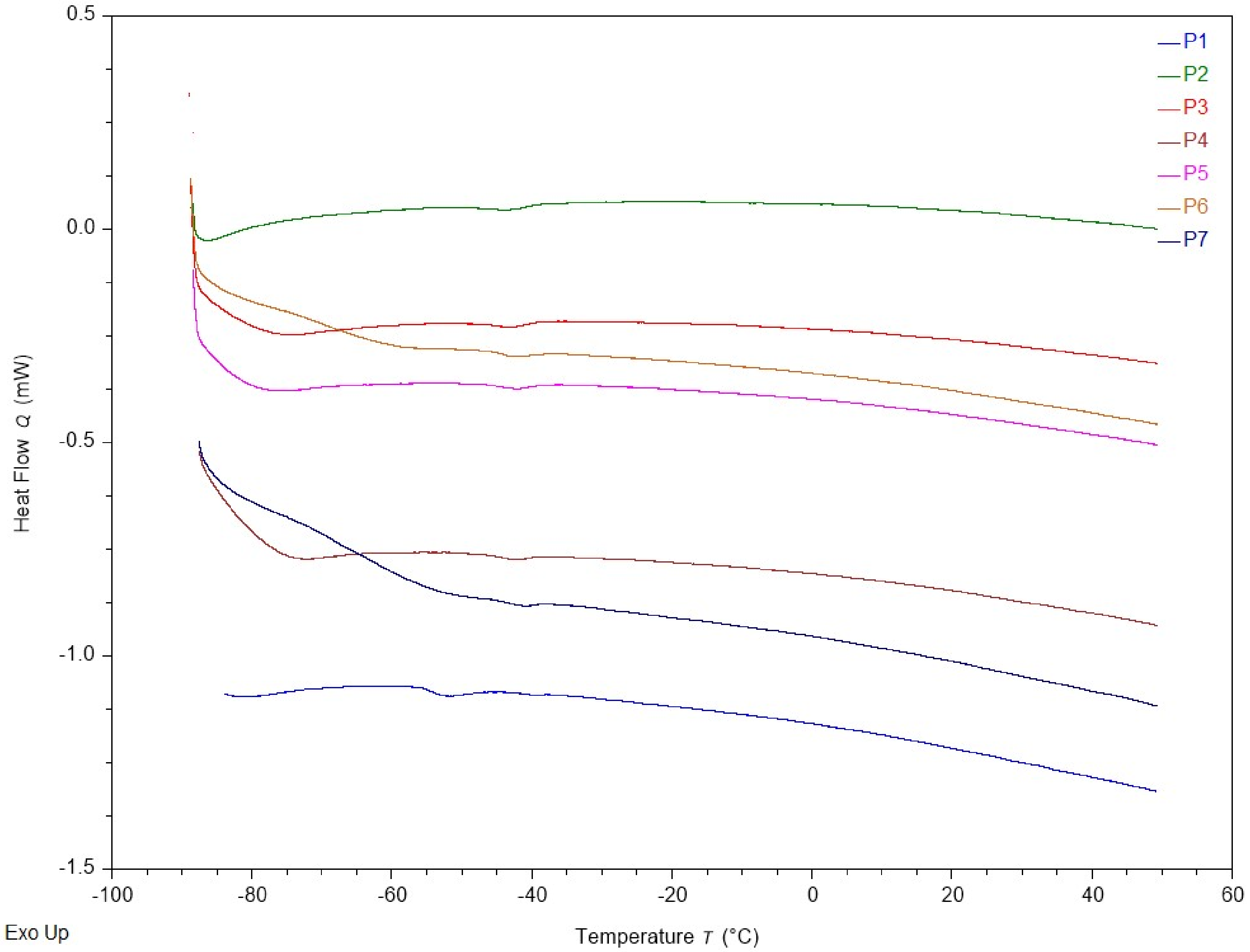

Figure S48. DSC thermograms of copolymers **P1−P7**.

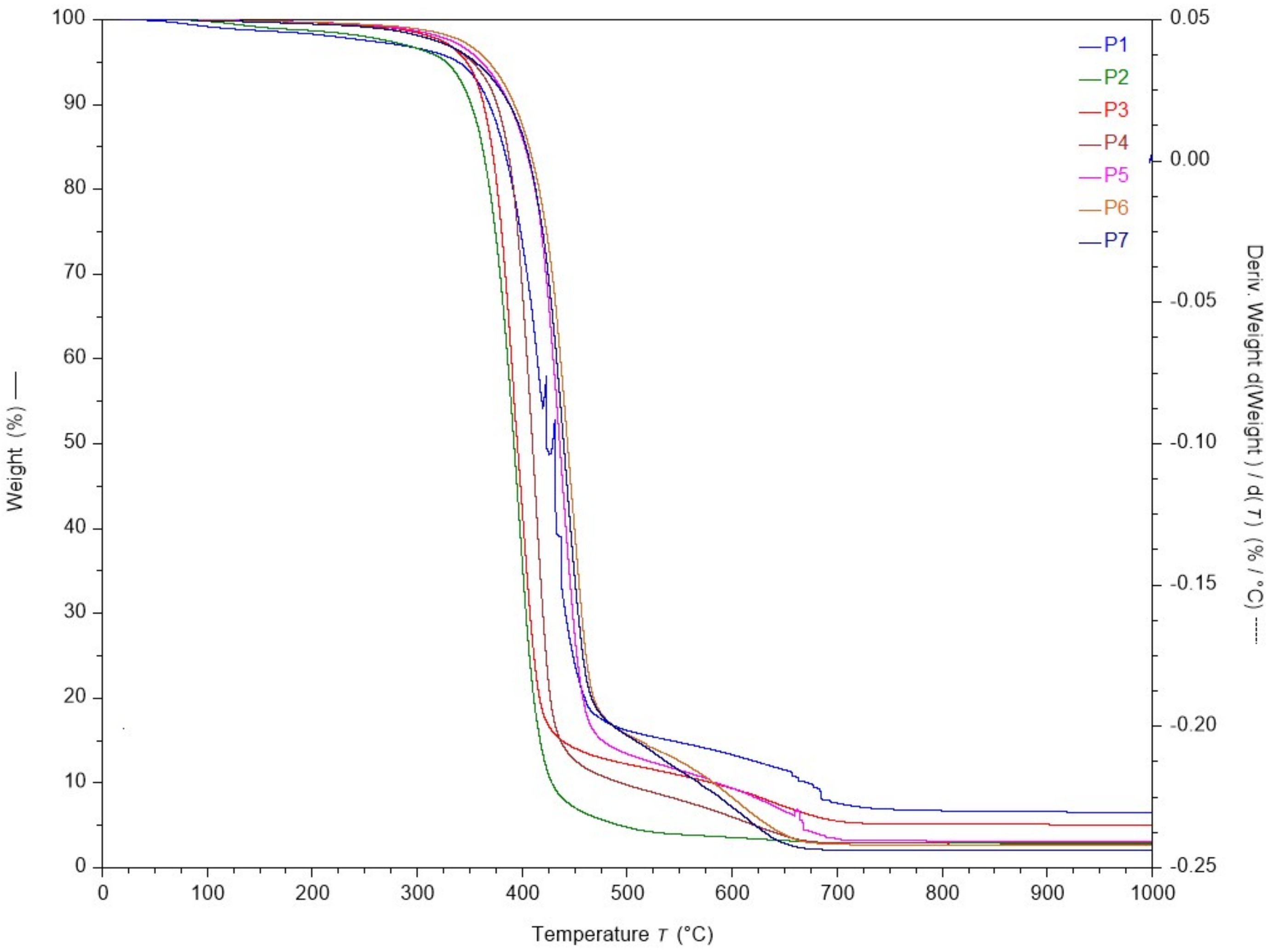

Figure S49. TGA analysis of copolymers **P1**–**P7**.

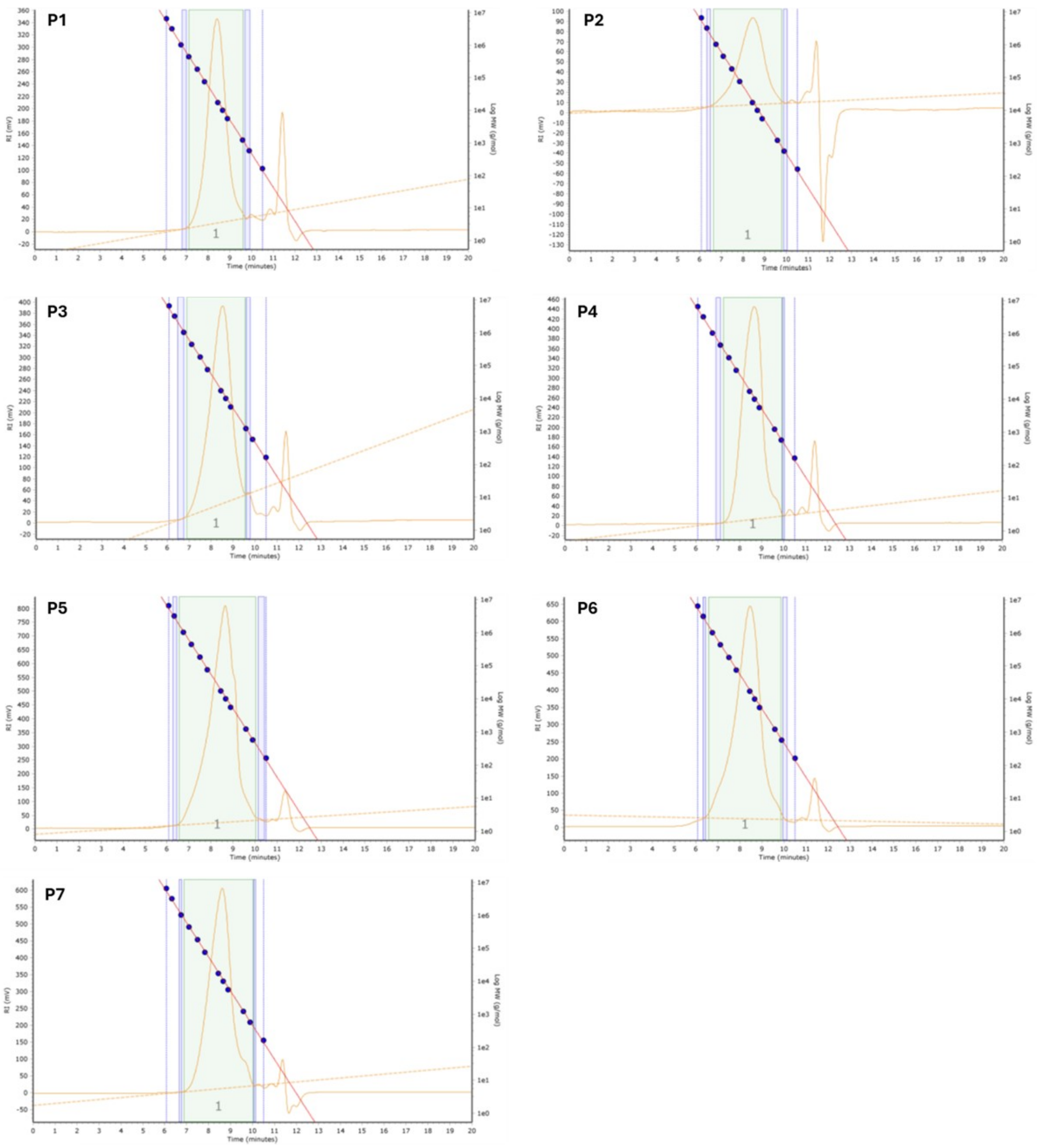

Figure S50. GPC analysis of segmented copolymers **P1**−**P7**.
